# Messages that increase COVID-19 vaccine acceptance: Evidence from online experiments in six Latin American countries

**DOI:** 10.1371/journal.pone.0259059

**Published:** 2021-10-28

**Authors:** Pablo Argote Tironi, Elena Barham, Sarah Zuckerman Daly, Julian E. Gerez, John Marshall, Oscar Pocasangre

**Affiliations:** Department of Political Science, Columbia University, New York, NY, United States of America; Bucharest University of Economic Studies, ROMANIA

## Abstract

As safe and effective vaccines become widely available, attaining herd immunity and limiting the spread of COVID-19 will depend on individuals choosing to vaccinate—and doing so quickly enough to outpace mutations. Using online surveys conducted across six Latin American countries in January 2021, we experimentally assess messages designed to counteract informational deficiencies and collective action problems that may drive hesitancy. We first find that basic vaccine information persuades around 8% of hesitant individuals to become willing to vaccinate, reduces intended wait to vaccinate by 0.4 months, and increases willingness to encourage others to vaccinate. Rather than facilitating free riding, learning, or social conformity, additional information about others’ behavior increases vaccine acceptance when respondents expect herd immunity will be achieved. Finally, priming the social approval benefits of vaccinating also increases vaccine acceptance. These results suggest that providing information and shaping social expectations and incentives could both significantly increase vaccine uptake.

## Introduction

The COVID-19 pandemic has inflicted significant suffering across the globe, but the rapid development and production of safe and effective vaccines provides the basis for emergent mass vaccination campaigns to contain the pandemic. Though vaccines have become widely available in the Global North, the success of mass vaccination campaigns will depend on sufficiently large numbers of people in every part of the world choosing to get vaccinated to prevent the spread of the virus and facilitate the return of normal life. Since it is also essential for vaccination to outpace virus mutations, it matters both *if and when* populations are willing to vaccinate.

However, quickly reaching the 60%-90% uptake rates that experts believe are required to achieve herd immunity within a given community will be challenging [1, 2]. Polls conducted between mid 2020 and early 2021 generally suggest that fewer than 75% of individuals are willing to get vaccinated in many countries [3–8]. However, in some countries like the United States, where vaccines are now available to all adults, vaccination uptake subsided once about half the adult population was vaccinated. It is likely that the initial increase in enthusiasm resulting from the rollout of vaccination campaigns [4] reflected the uptake in vaccination by the most willing “always-takers.” Fewer studies ask how quickly individuals would vaccinate once a vaccine is available to them. Those that do examine such timing find that around half the population intends to wait more than 3 months [4]. If vaccine uptake is insufficient to attain herd immunity, or is too slow to prevent vaccine-resistant mutations, the pandemic is likely to last significantly longer.

These challenges are both pertinent and of immediate importance in Latin America, where the mortality and socioeconomic impacts of COVID-19 have been substantial and vaccination campaigns that began only recently are expected to continue into 2022. In line with high levels of skepticism of science [9], prior studies suggest that vaccine willingness generally lies between 50% and 60% in Argentina, Chile, and Perú and 70% and 80% in Brazil and México. In comparison with the Global North, relatively little is yet known about whether and how Latin Americans can be encouraged to take vaccines against COVID-19. These questions may be especially significant in the Global South, where more limited distribution channels than in the Global North may increase the costs of accessing vaccines, but encouraging vaccination is no less important for both mitigating human suffering and restricting the emergence and spread of new variants of the virus.

To understand how mass vaccination campaigns can overcome individuals’ hesitancy, we leverage socialscientific theoretical frameworks that highlight how information and collective action problems can inhibit individually and socially optimal behaviors. The information transmission problem, whereby individuals lack exposure to credible information about the private costs and benefits of vaccination, may decrease vaccine willingness among risk-averse and uninformed individuals. Indeed, emerging COVID-19 research predominantly in the Global North has suggested that vaccine willingness is responsive to both expert information [10] and misinformation [6], although corrective messaging regarding vaccines for other diseases has produced less sanguine effects [11–13]. It is thus important to establish whether and what type of information about COVID-19 vaccines can increase vaccine acceptance.

Beyond an individual’s isolated health calculations, theories of collective action emphasize that information about the (expected) behavior of others could influence individual willingness to vaccinate—especially among hesitant individuals that perceive limited private benefits of vaccinating—in various ways [14]. Among vaccine hesitant individuals, learning that many others will vaccinate could reduce their vaccine willingness by causing them to “free ride” on the safety provided by others being vaccinated [15–17]. In contrast, learning that many others will vaccinate could instead increase vaccine acceptance to the extent that individuals draw inferences about the costs and benefits of vaccinations from the aggregated decisions of others [18, 19] or update their perceptions of what is required to conform with community norms [8, 10, 20]. However, any motivation to coordinate behaviors may also depend on participating in a *successful* collective effort [21–24], such that vaccination becomes more desirable when an individual expects to participate in a campaign that successfully achieves herd immunity. Since information about others’ behaviors could both increase or decrease vaccine acceptance, understanding the potential social drivers of vaccination also has important implications for public messaging.

Another encouragement highlighted by collective action research is “selective incentives”—private benefits that accrue indirectly only by taking the pro-social action [17]. Prior studies in economic, public health, and political domains suggest that getting vaccinated could generate selective incentives through social approval within an individual’s community network [25–30], an altruistic “warm glow” from helping others in the community [31, 32], or improving individual or communal income or employment prospects [33, 34]. Priming these motivations could encourage vaccination by creating reasons to vaccinate beyond the direct health benefits accruing to individuals and those immediately around them.

To evaluate which types of messages can overcome COVID-19 vaccine hesitancy, we embed a randomized experiment in a large online survey fielded in six Latin American countries before vaccines had become generally available to citizens. At the time of the survey in January 2021, uncertainty about vaccines and public health misinformation were prevalent—and remain so today. The treatments seek to establish the degree to which vaccine acceptance—in terms of both willingness to ever get vaccinated and how long an individual would wait to get vaccinated—can be increased by; (i) addressing the information transmission problem, by providing basic information about the safety and efficacy of COVID-19 vaccines; (ii) updating beliefs about the behavior of others, by further informing respondents of expert opinion regarding the share of the population that will need to vaccinate to achieve herd immunity and the share of the population that is currently willing to do so; and (iii) priming selective incentives relating to social approval, altruism, and economic recovery. We focus on the subpopulation that is hesitant about taking a COVID-19 vaccine—those who are either unwilling or uncertain about getting vaccinated quickly. Beyond illuminating the informational and social bases for vaccine hesitancy, our experimental analyses seek to assess how vaccine attitudes can be shaped by public messaging, which could inform the mass campaigns designed to increase vaccine willingness across Latin America and elsewhere in the Global South.

## Materials and methods

**Fig 1:**
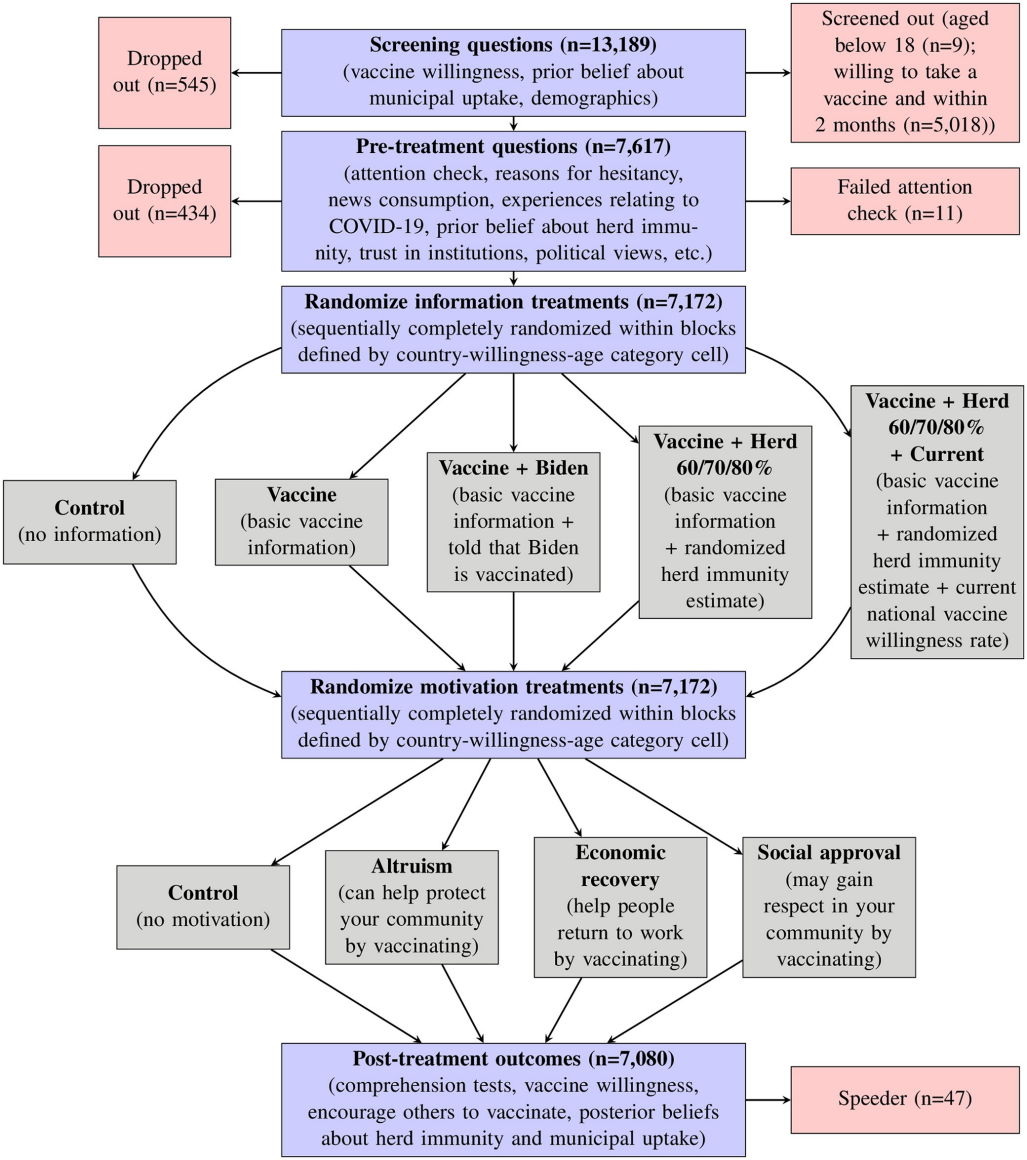
Overview of survey flow and treatment assignments. The n refers to the number of respondents that reach a given box. The full survey questionnaire is included in S18 Appendix.

For our single-wave between-subjects study, we recruited around 2,000 adults from large online panels maintained by Netquest in each of Argentina, Brazil, Chile, Colombia, México, and Perú. These six countries rank among the most populous and worst hit by the pandemic in Latin America [35, 36]. Given that Netquest’s opt-in panels include at least 125,000 individuals in each country, we obtained samples within each country that are broadly nationally representative by age, gender, socioeconomic level, and region, according to recent national censuses; we also reweigh our data to ensure representativeness along these dimensions. The online surveys were conducted between January 11 and January 29, 2021. Fig 1 depicts the flow of the survey, which took the median respondent 26 minutes to complete in the Qualtrics survey platform. S1 Appendix describes the sample of individual respondents in detail.

### Descriptive data on vaccine willingness in Latin America and screening

**Fig 2:**
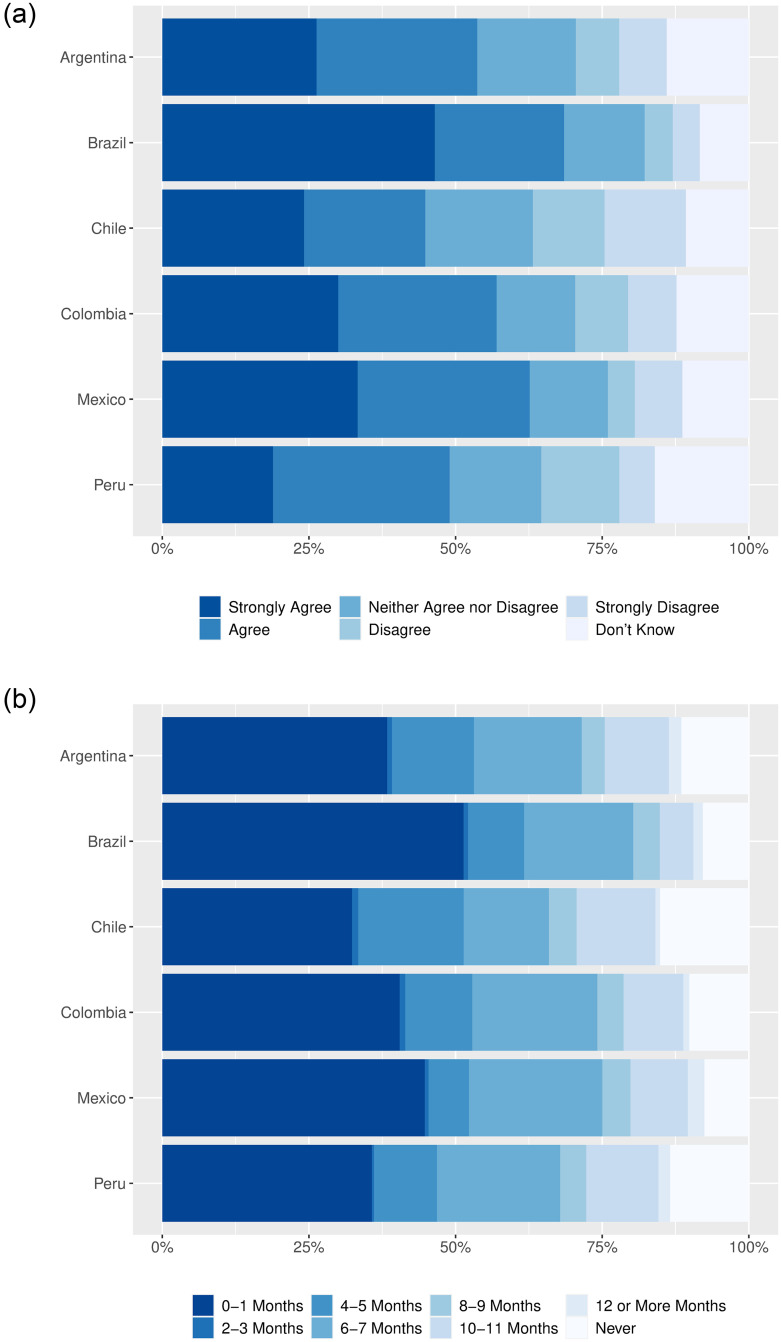
Distribution of vaccine willingness across countries (January 11–29, 2021). The questions for each figure were asked at the beginning of the survey of all participants. Observations are weighted to match the joint distribution over education, sex, region, and age category from the most recent census in each country (a) “If a vaccine were available to me now, I would get vaccinated.” (b) “If a vaccine were available to you now, how many months would you wait before getting vaccinated?”.

We first elicited respondents’ willingness to accept a vaccine once available to them and how soon they would take it (top-coded at 12 months). The results in Fig 2 suggest that, absent messaging interventions or additional incentives, herd immunity may be difficult to achieve: only 59% of our sample agreed or strongly agreed that they would take a vaccine once it were available to them, while the average respondent would wait 4.3 months before getting vaccinated. Such hesitancy varies across countries, with willingness ranging from 50% in Chile to 68% in Brazil and from 5.1 months in Chile to 3.5 months in Brazil. Given high levels of mobility within Latin America, all countries could be reduced to the lowest common denominator as borders reopen, further risking the ability of the current generation of vaccines to limit the impact of the pandemic [37].

**Fig 3:**
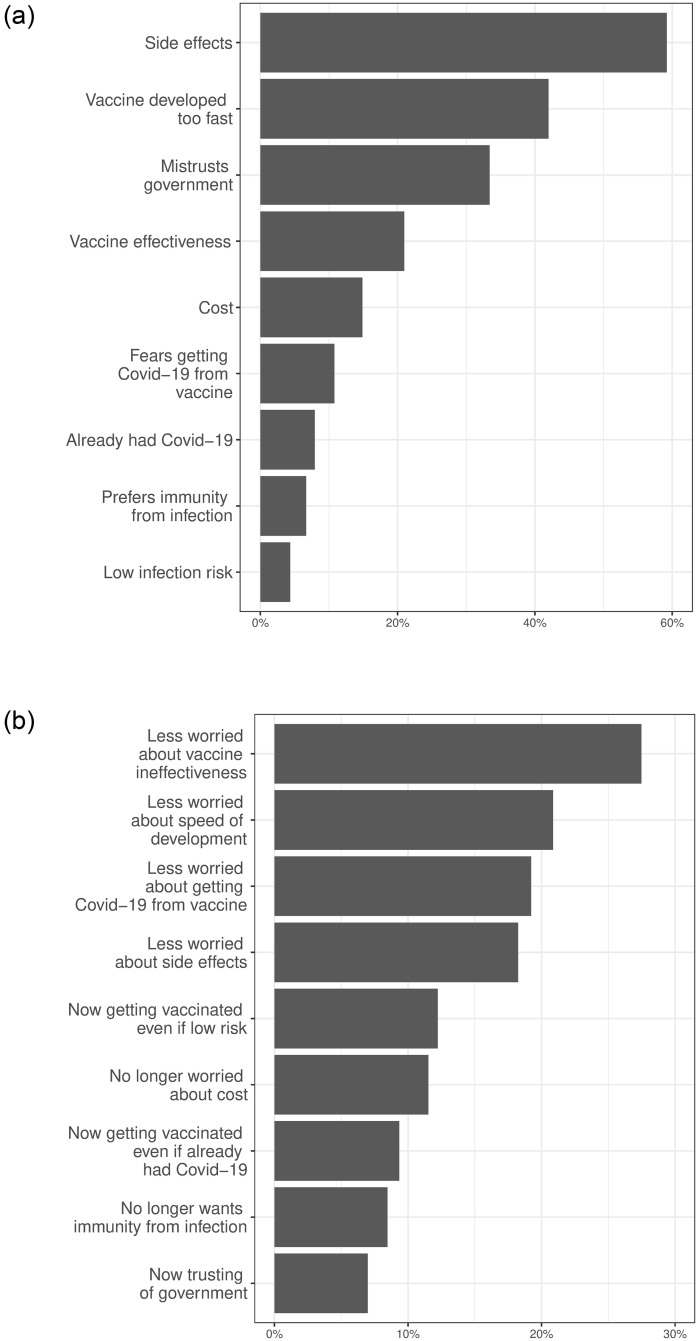
Reasons for initial vaccine hesitancy and response to vaccine information treatments. Panel (a) reports the percentage of hesitant respondents that chose each reason for hesitancy from a multi-response list. Panel Panel **(b)** reports the percentage of respondents that received a vaccine information treatment that chose each reason when asked how the vaccine information affected their concerns about COVID-19 vaccines. The exact questions and responses are shown in S18 Appendix.

To focus attention on how hesitant individuals respond to our informational and motivational interventions, we screened out respondents who agreed or strongly agreed that they would take a vaccine once available to them *and* would take it within two months of becoming eligible. The survey proceeded for around 1,200 vaccine-hesitant individuals in each country. As Fig 3a shows, the primary concerns of these hesitant respondents regarded the vaccines’ potential side effects (59%), the speed with which the vaccines were developed (42%), mistrust in government (33%), and skepticism of the vaccines’ effectiveness (21%). We next describe the treatments that we designed to overcome such concerns about the private health net benefits of COVID-19 vaccination, as well as to capture social and selective incentives that may encourage vaccine uptake among those that remain skeptical.

### Treatment conditions

#### Informational treatment conditions

The common component of our vaccine information treatments provided basic facts about COVID-19 vaccines with the goal of informing respondents’ private health cost-benefit calculations. The specific informational deficiencies we sought to redress included: that approval of COVID-19 vaccines was based on rigorous medical trials; that these trials show the vaccines are safe and effective at preventing mild and severe forms of COVID-19; and that the side effects are generally minor and the vaccines cannot give you COVID-19. These facts have been the crux of most vaccine messaging campaigns in Latin America and beyond. The full script for each treatment condition, in English and Spanish, is reported in S2 Appendix.

We further investigate what additional types of information could complement the provision of basic vaccine information. As Fig 1 illustrates, we supplemented the basic information treatment in two ways designed to capture potentially important features of citizen behavior emphasized by social scientific theories—the roles of information credibility and collective action dynamics. Our first additional treatment further informed respondents that U.S. President Joseph Biden had already been vaccinated. This supplementary information aimed to reinforce the credibility of the basic vaccine information by documenting the behavior of someone who would be unlikely to act that way if the information were untrue [38, 39] or—as a vaccine endorsement by Dr. Anthony Fauci appears to achieve in the U.S. [40]—by documenting the behavior of someone with access to medical expertise. When we fielded the survey in January 2021, very few people in Latin America—including none of the Presidents in our sample of countries—had yet been vaccinated. President Biden then represented a reasonable choice for a public figure who might be viewed as unlikely to get vaccinated if the vaccines were not safe.

Second, while basic health information may shift perceptions of the individual health benefits of vaccination, collective factors could be just as important in influencing vaccine uptake. To understand how expectations of others’ behavior shapes individual decisions, six further treatment conditions provided information about the national population’s need and willingness to vaccinate, in addition to the basic vaccine facts just described.

The first three treatment conditions varied whether respondents were informed that 60%, 70%, or 80% of the population would need to be vaccinated to achieve herd immunity. These numbers, which include low and high expert opinions, were chosen to reflect the differences in opinion among experts at the time the survey was fielded [1]. By varying expectations of the level of vaccination needed to achieve herd immunity, we seek to assess whether a greater difficulty of achieving herd immunity reduces willingness to vaccinate or increases willingness to vaccinate. A negative effect could result from increased incentives to free ride by reducing an individual’s marginal effect on achieving herd immunity. A positive effect could arise by coordinating expectations around the need for mass vaccine uptake. In addition to comparing individuals exposed to expert opinions of higher or lower herd immunity levels, we can test these hypotheses by comparing respondents for whom the expert opinion that they received exceeded or fell below the respondent’s prior belief about the rate of vaccination needed to achieve herd immunity.

The second three conditions relating to collective factors more directly test how vaccination decisions depend on expectations of whether other individuals will actually get vaccinated. Following the approach of researchers studying protest participation [14], these conditions reported the share of the population willing to be vaccinated in the respondent’s country, based on recent studies (for early respondents) or on initial data from our survey (for the majority of respondents) in addition to the basic vaccine information and one of the three herd immunity expert opinions previously described. By updating respondents’ expectations about the intended behavior of others, the additional information about intended uptake rates could shape incentives to “free ride” on the safety provided by others getting vaccinated [17], induce social learning about the health benefits of vaccination [18], alter perceptions of how to conform with societal norms [20], or update respondent beliefs about the likelihood that getting vaccinated will make them part of a successful collective effort [24]. We test the different implications of these hypotheses by examining whether respondents for whom the current level of willingness exceeded or fell below a respondent’s prior belief about vaccine uptake rates—or, in the case of wanting to be part of a collective effort, the expert opinion on the level of vaccination required to achieve herd immunity—became more or less willing to vaccinate.

After each element of the treatment was delivered, (non-incentivized) comprehension questions helped respondents absorb the facts provided; the respondent’s correct and incorrect answers to these questions were shown after each question. Manipulation checks later in the survey confirm that respondents internalized non-tested information as well (see S4 Appendix). In addition to the eight treatment conditions, we also included a pure control group that received no health information. The design ultimately enables us to compare basic vaccine information or its combination with supplemental information against a control group receiving no such information, as well as to compare the different supplemental information treatments against each other and against the receipt of only the basic vaccine information.

#### Motivational treatment conditions

After potential exposure to information about the vaccines and population behaviors, we further examined messages seeking to prime selective incentives to get vaccinated. Motivated by theories of pro-social behavior when the direct private benefits—here, the individual’s personal health benefits of vaccinating—of action are regarded as limited, we consider three types of selective incentives that may increase the return to getting vaccinated. First, a social approval message highlighted that, by vaccinating, individuals can show others that they care about their community, and may then gain respect and approval from others in their community. This message seeks to prime respondents that care about others’ perceptions of them to consider how getting vaccinated can signal their public-mindedness to others [41]. Second, an altruistic message aimed to activate a “warm glow”—the satisfaction that individuals receive from helping others, whether due to the benefits that others experience or the joy derived from the act of helping [31]—which emphasizes that, by vaccinating, respondents would be contributing to healthier communities and protecting vulnerable populations. Finally, an economic message explained that stopping the spread of COVID-19 is required to help people return to work and therefore, by vaccinating, respondents would be helping the economy recover. This condition seeks to test whether priming the link between vaccination and individual or communal economic prospects generates additional incentives to vaccinate. We compare the impact of these messages against one another, as well as relative to a control group that received no motivational message.

The informational and motivational treatment conditions were cross-randomized, such that respondents could receive one condition from each category. Treatment assignment followed a block randomization procedure that randomly assigned each treatment condition within 144 blocks of respondents defined by their country, initial vaccine willingness, age category, and the time they took the survey. We estimate treatments effects using OLS regressions that adjust for block fixed effects and pre-treatment measures of the outcome, while weighting respondent observations by the inverse probability of treatment assignment; inference is based on robust standard errors and two-tailed *t* tests. S3 Appendix explains in detail the experimental design and core estimation strategies, which we pre-registered in the Social Science Registry (www.socialscienceregistry.org/trials/7080) before the end of data collection.

### Measurement of vaccine willingness outcomes

Repeating the screening questions shown in Fig 2 several questions after treatments were delivered, our three primary outcomes are: (i) the five-point agree-disagree scale of vaccine willingness, (ii) an indicator for whether a respondent agrees or strongly agrees that they would get vaccinated if a vaccine were available, and (iii) the number of months that a respondent would wait to get vaccinated (which we reverse so positive coefficients always imply greater willingness). In addition to capturing the speed with which vaccine uptake may occur, the intended wait also provides a more fine-grained alternative measure of general vaccine willingness. Furthermore, we investigate social influence—which could play a key role in diffusing messages and consolidating beliefs within communities where engagement with government and media messaging is low or such institutions are not trusted—by asking whether respondents would encourage others to get vaccinated. We focus on an indicator for respondents that are somewhat or very likely to encourage others to get vaccinated, although similar results hold using a four-point scale (see S15 Appendix).

Since our experiment was designed to help inform vaccination communication campaigns as the general public becomes eligible, our analyses pertain to vaccination *intentions* because hardly any individuals were eligible to vaccinate at the time. Previous studies suggest that messaging campaigns can scale up to influence mass health behaviors in other domains [42]. Moreover, the risk that respondents answer to please the researcher are likely to be limited by the impersonal online nature of the survey [43]; in line with this, we find no evidence of differential effects among more educated respondents that may be more likely to understand the structure of the study or demonstrate pro-social intentions when primed (see S10 and S14 Appendices, respectively). Nevertheless, future studies will be required to validate whether encouragements that affect intentions ultimately influence actual decisions to vaccinate.

### Ethics statement

The full set of experimental protocols was approved by Columbia University’s Institutional Review Board (protocol number IRB-AAAT5273). Consent to participate in the study was obtained online after details of the study were described to potential participants.

## Results

### Basic vaccine information increases vaccine willingness

**Fig 4:**
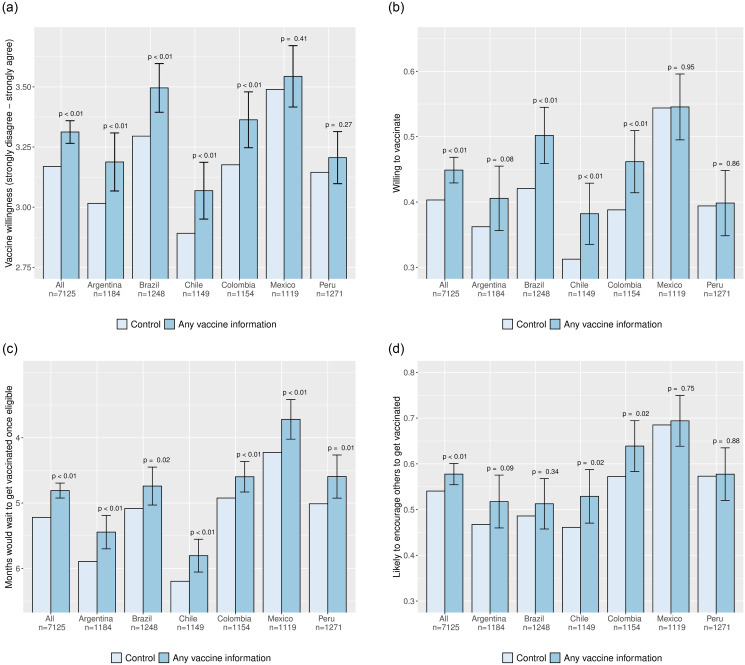
Average effects of any vaccine information treatment on vaccine willingness, by country. Each bar depicts a group outcome mean. The outcome in panel **(a)** is a five-point vaccine willingness scale ranging from “strongly disagree” (1) to “strongly agree” (5); the outcome in panel **(b)** is an indicator for “agree” or “strongly agree”; the outcome in panel **(c)** is the (reversed) number of months that a respondent would wait to get vaccinated once eligible for a vaccine; and the outcome in panel **(d)** is an indicator for a respondent being “somewhat likely” or “very likely” to encourage others to get vaccinated. Error bars denote 95% confidence intervals for treatment effects relative to the control group; the associated *p* values are from two-sided *t* tests. The underlying regression specifications for each outcome are described in S3 Appendix and the underlying regression table is reported in S6 Appendix.

**Fig 5:**
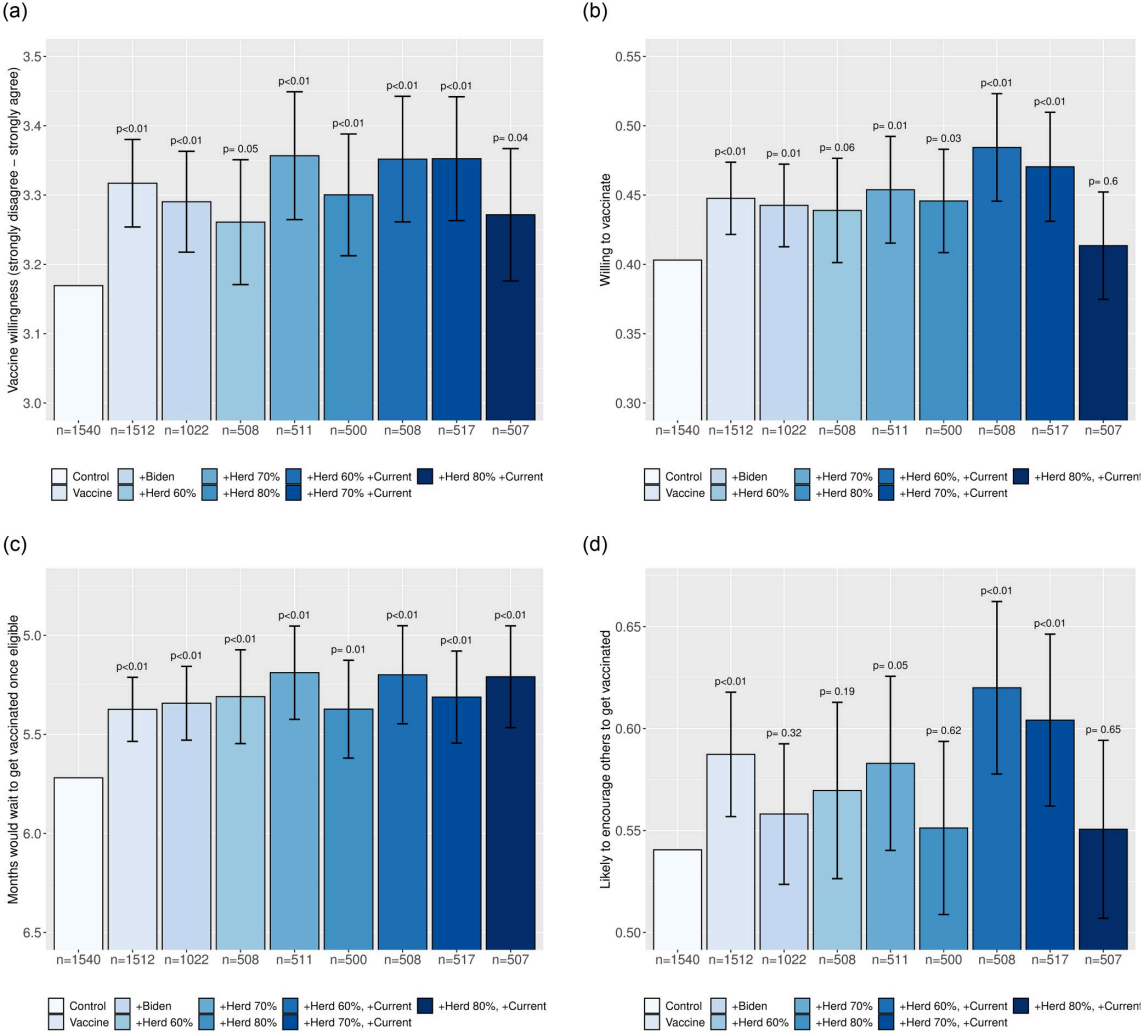
Average effects of vaccine information variants on vaccine willingness. Each bar depicts a group outcome mean, with the sample size in each group reported below. The outcome in panel **(a)** is a five-point vaccine willingness scale ranging from “strongly disagree” (1) to “strongly agree” (5); the outcome in panel **(b)** is an indicator for “agree” or “strongly agree”; the outcome in panel **(c)** is the (reversed) number of months that a respondent would wait to get vaccinated once eligible for a vaccine; and the outcome in panel **(d)** is an indicator for a respondent being “somewhat likely” or “very likely” to encourage others to get vaccinated. Error bars denote 95% confidence intervals for treatment effects relative to the control group; the associated *p* values are from two-sided *t* tests. The underlying regression specifications for each outcome are described in S3 Appendix and the underlying regression table is reported in S6 Appendix.

We start by pooling all eight treatment conditions that provide basic facts about the COVID-19 vaccines—what they do, how they were developed, that they are efficacious, and do not cause major side effects. As Fig 4 illustrates, the receipt of any information about vaccines significantly increased vaccine acceptance among the hesitant in Latin America. Panel B shows that receiving this information increased the probability of respondents agreeing or strongly agreeing that they would get vaccinated by 0.046 probability points (95% CI: 0.026 to 0.065). Since 40% of control respondents agreed with this statement, the treatment effect implies that 7.7% of the hesitant were persuaded to take a vaccine. In addition to increasing willingness to vaccinate, panel C shows that vaccine information also reduced the average time that a respondent would wait to vaccinate by 0.41 months (95% CI: 0.30 to 0.52), or about 0.1 standard deviations of the control group distribution. Panel D further shows that vaccine information also increased the probability that respondents would encourage others to get vaccinated by 0.037 probability points (95% CI: 0.014–0.060). Fig 5 reports similar results when comparing the control group with the treatment that only provided basic vaccine information. As Fig 3a illustrates, these effects appear to be driven by reducing concern that the vaccines will be ineffective, were developed too fast, would give people COVID-19, and would produce serious side effects. These results collectively suggest that vaccine hesitancy is, in part, driven by limited information about the safety and efficacy of the COVID-19 vaccines; at the same time, our results show that vaccine acceptance can be increased by providing information to quell the main concerns of vaccine hesitant Latin Americans.

The effect of receiving any vaccine information is remarkably similar on hesitant individuals that vary in terms of observable characteristics that could be used as the basis for targeting mass information campaigns. First, Fig 4 shows that the information statistically significantly increased the speed with which individuals reported that they would get vaccinated in each of the six countries under study, their willingness to vaccinate in all countries but México and Perú, and the likelihood of encouraging others to vaccinate in Argentina, Chile, and Colombia. These results suggest that simple factual information about vaccines can overcome the concerns of hesitant individuals in various contexts across Latin America.

Second, as we show in S9 Appendix, we do not observe substantial differences in persuasion across different types of respondent, except by gender. Although the treatment also increased the willingness of men to vaccinate, it was roughly twice as effective among women. This descriptive finding chimes with prior research arguing that women are more risk averse and likely to seek out information pertaining to the health benefits of vaccination [44–46], and suggests that mass campaigns emphasizing basic vaccine information may be more effectively targeted at women. However, we could not detect statistically significant differences in response to treatment by age group, educational attainment, socioeconomic group, or intention to vote for the incumbent President.

We next compare levels of vaccine acceptance between the group that received only basic vaccine information and the groups that also received additional information. The results, as shown in Fig 5 show that neither informing respondents about the levels of vaccination required to reach herd immunity or current vaccination intentions nor informing respondents that President Biden was vaccinated systematically produced additional effects on vaccine willingness on average. With the exception of the current willingness treatment combined with 80% being required for herd immunity (discussed below), we cannot reject the null hypothesis that the average effect of the other seven vaccine information treatments on the three individual willingness outcomes is identical. Further analyses detect no statistically significant differences in the reasons given for becoming less hesitant between the different information treatments (see S8 Appendix). The results thus suggest that respondents found the basic vaccine information credible without the “do as I do” endorsement of a prominent public figure in the United States and do not respond to herd immunity information *on its own*.

### Vaccine willingness is not shaped by free riding, social learning, or social conformity

Theories of peer effects predict that the response to information about the expected behavior of others will vary across individuals, depending on how the information relates to their prior beliefs. This focus on heterogeneous effects differs from the previous section, which focused on *average effects*.

However, we find no evidence to suggest that vaccine willingness is driven by free riding, social learning, or social conformity. Indeed, receiving information about the current level of vaccine willingness in their country *did* cause respondents to substantially update their beliefs about vaccine uptake in line with whether reported willingness was above/below a respondent’s prior belief. However, these changes in beliefs *did not* translate into intended behaviors: being informed that current levels of willingness is above (below) prior expectations did not decrease (increase) an individual’s vaccine acceptance, as free riding would predict. Similarly, being informed that willingness is above (below) expectations did not increase (decrease) an individual’s vaccine acceptance, as social learning or a desire to conform with the behavior of others in the population would predict. S11 Appendix reports these null findings in detail.

### Expecting a vaccination campaign to achieve herd immunity increases vaccine willingness

**Fig 6:**
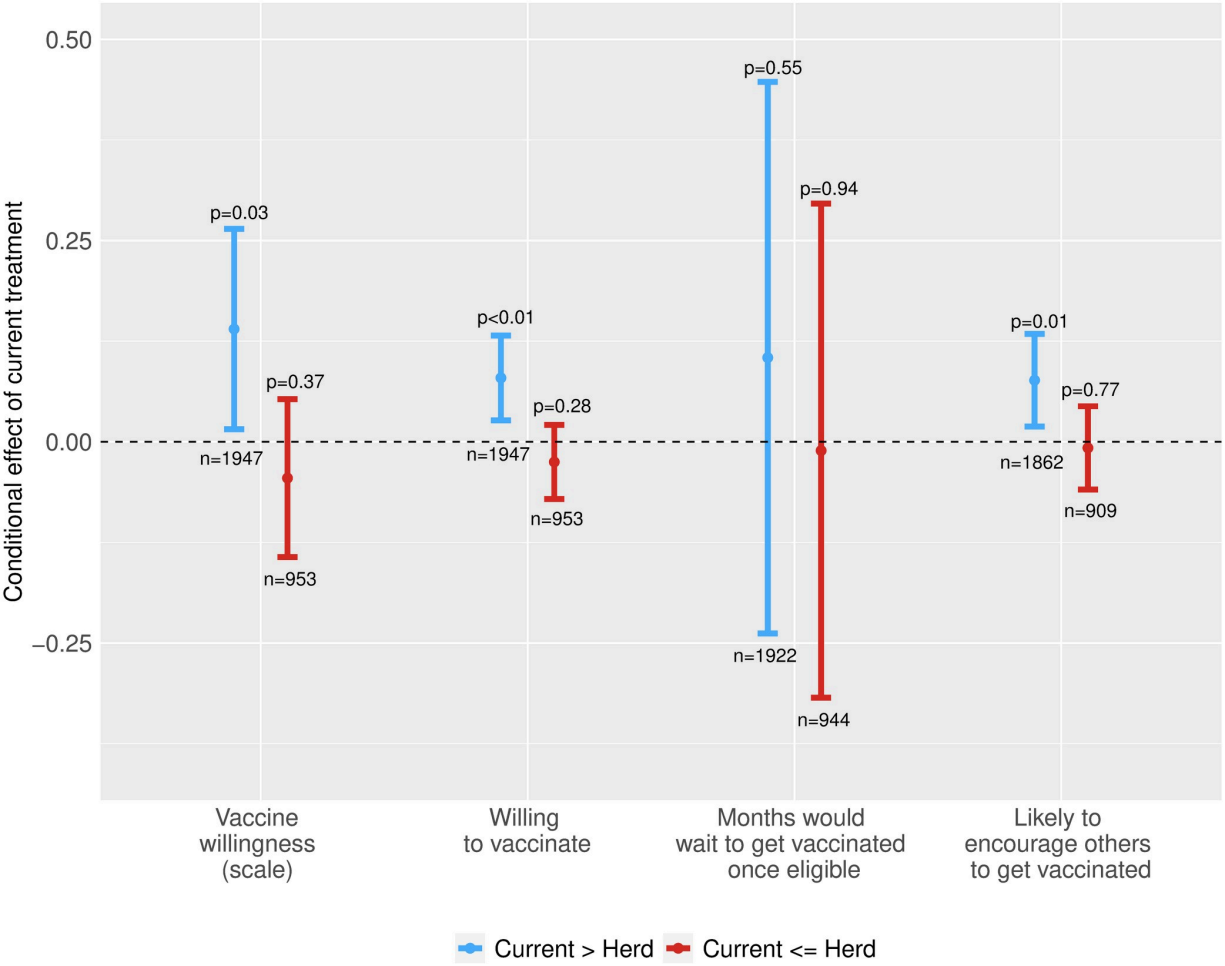
Effects of currently expected willingness information on vaccine willingness, by whether current willingness is above or below the expert herd immunity opinion a respondent was exposed to. (a) Vaccine willingness scale. (b) Willing to take a vaccine. (c) Months would wait to get vaccinated. (d) Encourage others to get vaccinated. Each bar depicts a 95% confidence interval for the conditional average treatment effect of receiving the currently expected national willingness rate treatment, relative to just receiving an expert herd immunity opinion; the associated *p* values are from two-sided *t* tests and n captures the number of respondents in each subgroup. The outcome variables arrayed along the x axis are: a five-point vaccine willingness scale ranging from “strongly disagree” (1) to “strongly agree” (5); an indicator for “agree” or “strongly agree”; the (reversed) number of months that a respondent would wait to get vaccinated once eligible for a vaccine; and an indicator for a respondent being “somewhat likely” or “very likely” to encourage others to get vaccinated. The underlying regression specifications for each outcome are described in S3 Appendix and the underlying regression table is reported in S6 Appendix.

The role of social interactions may instead depend on expectations of whether herd immunity will be achieved in the respondent’s country. Exploring this further, Fig 6 examines how the effect of receiving current population vaccine willingness information alongside an expert herd immunity opinion, relative to only receiving an expert herd immunity opinion, varies with whether the currently expected national willingness rate that the respondent saw—which never exceeded 80% in any country, and averaged 67% across countries—was above or below the expert herd immunity opinion that respondents received.

The results indicate that expecting to be part of a successful vaccination effort increased vaccine acceptance by more than receiving basic vaccine information. Being informed that the currently expected national willingness rate exceeds the expert herd immunity requirement increased vaccine willingness by 0.079 probability points (95% CI: 0.027 to 0.131), whereas being informed that the currently expected national willingness rate is below the expert judgement may even have reduced vaccine willingness (95% CI: -0.061 to 0.011). The same dynamic is evident for the vaccine willingness scale, the speed with which individuals are willing to get vaccinated, and encouraging others to get vaccinated, although the effects are not statistically significant for the number of months that an individual would wait to get vaccinated. As explained in S3 Appendix, by conditioning on the level of willingness reported, these estimates are identified by the experimentally-induced variation in whether the expert herd immunity opinion exceeded or fell below the current level of national willingness reported to the respondent.

Consistent with these findings, our pre-treatment observational data further show that vaccine acceptance was greatest among respondents that expected both a high community uptake rate and high shares of vaccination to attain herd immunity (see S12 Appendix). Taken together, these results suggest that participating in a collective campaign that is expected to achieve herd immunity may inspire vaccine uptake. This could reflect intrinsic motivations to be part of a “winning team” or—as our next set of findings suggest—social incentives to be seen to be part of such a successful collective effort.

### Social approval increases vaccine willingness

**Fig 7:**
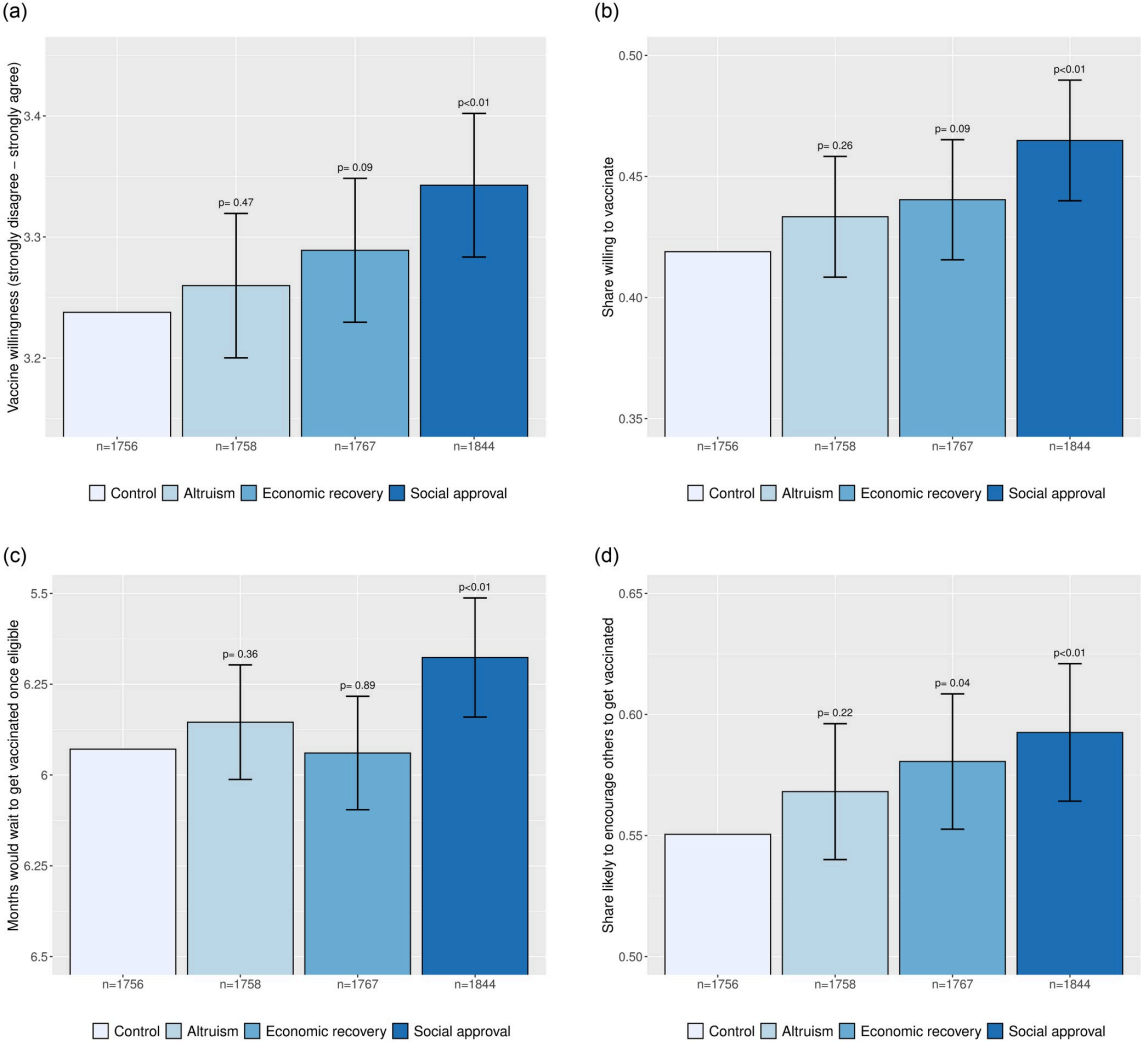
Average effects of motivational messages on vaccine willingness. Each bar depicts a group outcome mean. The outcome in panel **(a)** is a five-point vaccine willingness scale ranging from “strongly disagree” (1) to “strongly agree” (5); the outcome in panel **(b)** is an indicator for “agree” or “strongly agree”; the outcome in panel **(c)** is the (reversed) number of months that a respondent would wait to get vaccinated once eligible for a vaccine; and the outcome in panel **(d)** is an indicator for a respondent being “somewhat likely” or “very likely” to encourage others to get vaccinated. Error bars denote 95% confidence intervals for treatment effects relative to the control group; the associated *p* values are from two-sided *t* tests. The underlying regression specifications for each outcome are described in S3 Appendix and the underlying regression table is reported in S6 Appendix.

The desire to participate in a successful coordinated vaccination effort chimes with individuals’ responses to our motivation treatments. Comparing the social approval, altruistic, and economic recovery motivational messages, Fig 7 shows that priming the respect that individuals may receive in their community by getting vaccinated plays an important role in overcoming vaccine hesitancy. Specifically, the social approval treatment increased vaccine willingness by 0.046 probability points (95% CI: 0.021 to 0.071), which translates into persuading 7.9% of hesitant respondents—a level comparable to exposure to basic vaccine information. The 0.25 month reduction in how long respondents would wait to get vaccinated (95% CI: 0.09 to 0.42) is a little lower than for basic vaccine information, but non-trivial in magnitude when extrapolated to a national level. Priming the social incentives to get vaccinated also caused individuals to become more likely to encourage others to get vaccinated.

Comparing these effects across different types of individuals suggests that social approval motivates most subgroups. As we show in S13 Appendix, we fail to detect significant differences in the effect of treatment across gender, socioeconomic status, educational attainment, or intention to vote for the President. Although most age groups also responded similarly, there is some evidence to suggest that priming social approval is less effective at motivating respondents aged above 65 to vaccinate. Moreover, social approval neither substantially crowds out nor complements the effects of basic vaccine information; see S14 Appendix. Due to the small size of some subgroups, some caution should be exercised in interpreting these effects because the analyses could only detect substantial differences in treatment effect across subgroups.

In contrast, we find no evidence to suggest that priming altruistic motives encourages vaccine acceptance among hesitant Latin Americans. The economic recovery motivation, which could be interpreted either in selfish or pro-social terms, increased willingness to get vaccinated by 0.022 probability points (95% CI: -0.003 to 0.046), but was not statistically significant at the 95% level using a two-tailed test and had little effect on the number of months that a respondent would get vaccinated (95% CI: -0.17 to 0.15).

## Discussion

Across six major Latin American countries, we document moderate—albeit cross-nationally varying—levels of vaccine hesitancy. In January 2021, little more than half the adult population was willing to take a vaccine, while similar numbers would take a vaccine within 3 months of becoming eligible. Even if willingness increases as vaccine programs roll out, uptake may be too low or too slow to achieve herd immunity and prevent the spread of new COVID-19 variants that could overcome the immunity conferred by vaccines and recent exposure to the virus. By showing that hesitancy reflects informational and coordination problems, our results suggest that intended behaviors are malleable and effective public messaging could significantly increase both vaccine uptake and the speed of uptake among the hesitant. Our online experiment shows that providing basic information about vaccines, encouraging individuals to believe that they could be part of a successful collective effort, and harnessing the reputational benefits of vaccination that people expect to receive can all reduce vaccine hesitancy.

By illuminating the theoretical mechanisms that drive hesitancy of the COVID-19 vaccines and the types of messages that can overcome them, our findings can inform the design of public health communication strategies and vaccine distribution. In terms of communication strategies, we show that information about safety and efficacy counteracts skepticism about the new vaccines among hesitant individuals. Saturating public discourse or microtargeting more hesitant demographic groups with such information may then increase uptake in the population both by directly persuading individuals but also through social amplification mechanisms—given the apparent desire both for social approval and, once informed, to encourage others to vaccinate. Although the message may not convince ardent anti-vaxxers, it appears to resonate with many types of respondent that have concerns about the COVID-19 vaccines. It also remains possible that observing domestic figures—such as politicians or local health care providers, who had not yet generally been vaccinated when our study was fielded—could more effectively signal the credibility of basic vaccine information than foreign leaders like President Biden.

Our finding that vaccine willingness is not simply a private cost-benefit calculation further suggests that, in tandem with emphasizing the safety and efficacy of COVID-19 vaccines, policymakers may increase vaccine uptake by making vaccine uptake observable in at least two different ways. First, organic social approval mechanisms could be amplified by interventions through which individuals can show peers that they have been vaccinated. This could involve “I got vaccinated” stickers or wristbands, the use of vaccine passports, or ways of sharing vaccination status on social media. Second, rather than worry about free-riding or encouraging individuals to feel a warm glow from helping others, our findings suggest that policymakers should make aggregate uptake rates visible—whether in the news, through official briefings, or more direct messaging (in person or through ads)—as vaccination levels approach herd immunity. As our results indicate, the belief that vaccination rates will reach the level required to achieve herd immunity will encourage the hesitant to join a successful herd immunity drive. Such upbeat communication—which has been rare, relative to news coverage of low-probability risks associated with certain vaccines and concerns about fake news—might be enhanced by emphasizing winning together as a “team”, perhaps by including groups that inspire camaraderie like sports teams in campaign programming. Since the value of social approval could decline as vaccination rates increase [41], at the same time that the likelihood of attaining herd immunity increases, efforts to activate social dynamics may be most effectively sequenced to initially emphasize social approval mechanisms, before later shifting toward the positive messaging about reaching herd immunity.

The implications of our online experiment for the design of mass vaccinations campaigns are also limited in several ways. First, as our study was conducted before mass vaccination campaigns begin, we could not behaviorally measure vaccine uptake in the general population because vaccines were hardly available. Even though initial intentions translate into actual vaccination cannot be assessed until the general population becomes eligible, our results demonstrate that vaccine concerns can—at least temporarily—be overcome by suitable messages. Second, our messages were delivered once in a controlled survey context, rather than in a more complex environment where many messages compete and are repeated. While the effect of a single message is unlikely to endure until vaccines become generally accessible, communication campaigns may be able to achieve similar results by intensively delivering effective messages. Indeed, given that most government and civil society programs involve repeated exposure to information, further testing should identify the number of exposures required to consolidate vaccine willingness. Third, by focusing on encouraging hesitant respondents to vaccinate, we did not study whether the messages could discourage individuals that were already willing to vaccinate. Beyond weakening social approval incentives, backfiring of this form appears unlikely if individuals are more willing to vaccinate when others are vaccinating (and thus herd immunity is more likely to be achieved).

Despite these limitations, our evidence ultimately highlights the *types* of messaging and programming that may combat COVID-19 vaccine hesitancy in Latin America—and perhaps beyond, given related findings in the Global North [6, 8]. Although careful design is needed to generate policies that cultivate similar responses to the treatments in our controlled study environment, we show that campaigns to redress informational deficiencies and harness social dynamics could persuade hesitant individuals to vaccinate and thereby help countries more quickly vaccinate significant shares of their populations.

## Supporting information

S1 Appendix Survey registration, recruitment, and screening. (PDF)

S2 Appendix Vaccine information and motivational message treatment conditions. (PDF)

S3 Appendix Experimental design and estimation strategies. (PDF)

S4 Appendix Manipulation checks. (PDF)

S5 Appendix Measurement of outcome variables. (PDF)

S6 Appendix The main results in regression table form. (PDF)

S7 Appendix Identification checks. (PDF)

S8 Appendix Differential effects of vaccine information treatments on reasons given for reducing hesitancy. (PDF)

S9 Appendix Heterogeneity in the effect of basic vaccine information. (PDF)

S10 Appendix Heterogeneity in the effect of herd immunity information. (PDF)

S11 Appendix Heterogeneity in the effect of current willingness information. (PDF)

S12 Appendix Pre-treatment vaccine hesitancy and prior beliefs. (PDF)

S13 Appendix Heterogeneity in the effect of motivational messages. (PDF)

S14 Appendix Interaction between informational and motivational messages. (PDF)

S15 Appendix Effects on encouraging others to vaccine measured as a scale. (PDF)

S16 Appendix Demand for further information. (PDF)

S17 Appendix Population-weighed treatment effects. (PDF)

S18 Appendix Full survey questionnaire. (PDF)
