# Correction: Messages that increase COVID-19 vaccine acceptance: Evidence from online experiments in six Latin American countries

**DOI:** 10.1371/journal.pone.0335673

**Published:** 2025-10-29

**Authors:** The PLOS One Staff

The third author’s name is spelled incorrectly. The correct name is: Sarah Zukerman Daly. The correct citation is: Argote Tironi P, Barham E, Daly SZ, Gerez JE, Marshall J, Pocasangre O (2021) Messages that increase COVID-19 vaccine acceptance: Evidence from online experiments in six Latin American countries. PLoS ONE 16(10): e0259059. https://doi.org/10.1371/journal.pone.0259059

The publisher apologizes for the errors.
