# Supplementary material for: Messages that increase COVID-19 vaccine acceptance: Evidence from online experiments in six Latin American countries
Source: PLoS One. 2021 Oct 28;16(10):e0259059. doi: 10.1371/journal.pone.0259059 (PMC8553119; doi:10.1371/journal.pone.0259059)
Supplement: S1 Appendix — (PDF) [file pone.0259059.s001.pdf]

S1 Survey registration, recruitment, and screening

Recruitment

Respondents in each country were recruited via Netquest's online panels between January 11 and January 29, 2021. Netquest maintains large panels of survey respondents in most Latin American countries, including at least 125,000 panelists in all six countries in this study. Panelists are regularly invited to take surveys, although this is not their primary vocation. Netquest's dynamic enrollment protocols updated invitations to ensure that the sample frame was nationally representative in terms of sex, age category, socioeconomic status, and region. Upon clicking a link to participate, respondents reached a Qualtrics landing page, where information about the academic study was provided and consent to participate in the study was obtained. As the summary statistics in Table S1 verify, the marginal distribution of respondents that started the survey (i.e. reached our screening juncture) closely approximated the Census distribution for these variables. Unsurprisingly for an online survey, respondents are less representative in terms of education, which Netquest did not seek to balance with population averages.

Screening

In addition to screening out respondents who were already willing to take a vaccine within less than 2 months of it becoming available, we also screened out respondents aged below 18 (n=9) or who failed our attention check eleven questions into the main survey (by failing to correctly identify the capital city of their country; n=11). Given these few screen outs, our sample of hesitant respondents is also likely to be broadly nationally representative of this subgroup. The median completed survey lasted 26 minutes; respondents who completed the survey were compensated with approximately 3 US dollars. Respondents who took less than 10 minutes to complete the survey (n=47) were excluded from the experimental analyses.

	Argentina		Brazil		Chile		Colombia		Mexico		Peru	
	Survey	Census	Survey	Census	Survey	Census	Survey	Census	Survey	Census	Survey	Census
**Age**	44.34	47.33	40.69	41.34	44.54	44.18	39.82	42.54	39.17	42.44	39.7	41.99
**Male**	0.49	0.53	0.50	0.49	0.51	0.48	0.48	0.48	0.49	0.48	0.47	0.48
**Socioeconomic status**												
Low	0.17	0.13	0.28	0.26	0.33	0.42	0.49	0.43	0.33	0.33	0.49	0.42
Middle	0.77	0.8	0.64	0.66	0.58	0.48	0.40	0.45	0.45	0.46	0.45	0.50
High	0.06	0.07	0.07	0.08	0.10	0.10	0.11	0.12	0.21	0.21	0.06	0.08
**Education**												
None	0.00	0.13	0.06	0.11	0.01	0.00	0.01	0.05	0.00	0.14	0.01	0.05
Primary	0.13	0.43	0.11	0.49	0.07	0.23	0.02	0.38	0.03	0.16	0.01	0.20
Secondary	0.48	0.32	0.53	0.27	0.47	0.46	0.32	0.29	0.44	0.54	0.36	0.51
Higher	0.23	0.07	0.21	0.13	0.28	0.22	0.51	0.16	0.31	0.14	0.34	0.14
Other higher	0.15	0.06	0.09		0.18	0.10	0.14	0.11	0.22	0.01	0.28	0.10

Table S1: Summary statistics among pre-screened respondents by country. The survey data pertain to our pre-screened sample. The Census data is drawn from the most recent available Census data, with the exception of the data for socioeconomic level, which was provided by Netquest.
