# Supplementary material for: Messages that increase COVID-19 vaccine acceptance: Evidence from online experiments in six Latin American countries
Source: PLoS One. 2021 Oct 28;16(10):e0259059. doi: 10.1371/journal.pone.0259059 (PMC8553119; doi:10.1371/journal.pone.0259059)
Supplement: S2 Appendix — (PDF) [file pone.0259059.s002.pdf]

# S2 Vaccine information and motivational message treatment conditions

The following script shows the full information script received by different vaccine information treatment groups, in both English and then Spanish (the Portuguese translations are available upon request):

> *[Control and all treatment groups]* Latin American countries are beginning to distribute their first doses of vaccines.
> Los países de Latinoamérica están comenzando a distribuir sus primeras dosis de vacunas.
>
> *[All treated groups]* The next screen will provide **important information about these COVID-19 vaccines**.
> Vaccines are designed to **prevent disease**.
> After **extensive testing by medical experts**, different countries have approved the use of various vaccines against COVID-19.
> Clinical tests have shown that the vaccines are **safe and highly effective** in preventing mild and severe COVID-19 infections. The **side effects are generally minor** and you cannot get COVID-19 from the vaccine.
> La siguiente pantalla proporcionará **información importante sobre estas vacunas** contra el COVID-19.
> Las vacunas están diseñadas para **prevenir enfermedades**.
> Después de **pruebas exhaustivas realizadas por expertos médicos**, se ha aprobado el uso de varias vacunas contra el COVID-19 en diferentes países.
> Las pruebas clínicas han demonstrado que las vacunas son **seguras y altamente eficaces** en prevenir infecciones leves y graves de COVID-19. Los **efectos secundarios son generalmente menores** y no se puede contraer COVID-19 de una vacuna.
>
> *[Biden treatment group only]* A few weeks ago, **President Joe Biden safely received a vaccine** against COVID-19 in the United States.
> Hace algunas semanas, **el presidente Joe Biden recibió, de manera segura, una vacuna** contra el COVID-19 en los Estados Unidos.
>
> *[Herd and Current treatments conditions only]* If enough people get vaccinated against COVID-19, the coronavirus will stop spreading.
> Some experts say that **at least [60/70/80]% of people need to be vaccinated to prevent the spread of the coronavirus**.
> Si suficientes personas se vacunan contra el COVID-19, el Coronavirus dejará de propagarse.
> Algunos expertos dicen que **al menos [60/70/80]% de las personas necesitan vacunarse para evitar la propagación del Coronavirus**.

| | | Information about vaccines? | | | | | | | | | |
|---|---|---|---|---|---|---|---|---|---|---|---|
| | | | | | | | | *Vaccine + Herd + Current* | | | |
| | | | | *Vaccine + Biden* | *Vaccine + Herd* | | | | | | |
| | | *None* | *Vaccine* | | *60%* | *70%* | *80%* | *60%* | *70%* | *80%* | Pooled |
| **Motivational message?** | *None* | 3/56 | 3/56 | 1/28 | 1/56 | 1/56 | 1/56 | 1/56 | 1/56 | 1/56 | 1/4 |
| | *Altruism* | 3/56 | 3/56 | 1/28 | 1/56 | 1/56 | 1/56 | 1/56 | 1/56 | 1/56 | 1/4 |
| | *Economic recovery* | 3/56 | 3/56 | 1/28 | 1/56 | 1/56 | 1/56 | 1/56 | 1/56 | 1/56 | 1/4 |
| | *Social approval* | 3/56 | 3/56 | 1/28 | 1/56 | 1/56 | 1/56 | 1/56 | 1/56 | 1/56 | 1/4 |
| | *Pooled* | 3/14 | 3/14 | 1/7 | 1/14 | 1/14 | 1/14 | 1/14 | 1/14 | 1/14 | |

**Table S2: Informational treatments factorial design.** The numbers in each cell indicate the share of the sample randomized into each condition within each country.

> *[Current treatments conditions]* Recent data indicates that **X% of people in [COUNTRY] currently say they would get vaccinated** against COVID-19.
> Datos de esta encuesta indican que **X% de las personas en [COUNTRY] actualmente dicen que se vacunarían** contra el COVID-19.

The control group only received the basic text in black, while the Vaccine, Herd, and Current components of the information treatments were successively shown on further screens (the Current information was shown together with the Herd expert opinion). The expert opinion of the vaccination rate required to achieve herd immunity randomly varies across treatment variants reporting 60%, 70%, or 80%—the most frequently cited numbers cited by experts around the time the survey was designed. Respondents that received the Current component of the information treatment were informed of the rate of vaccine willingness in their country according to recent surveys (for the first around 200 respondents per country) or the early respondents to this survey (all subsequent respondents). The text was all shown in black, but the emboldened sections were emboldened within Qualtrics. In each treatment condition, respondents were given a quick quiz to ensure that they internalized key information on each screen.

The eight different treatment groups are described in Table S2. The probability distribution used for the randomization assignment of conditions is arrayed along the $x$ axis.

After receiving the information treatments described in the previous section, respondents were independently randomly assigned to receive a motivational message. A control group received no message, while the altruism, economic recovery, and social approval messages are shown below in English and then Spanish (the Portuguese translations are available upon request):

> *[Altruism]* Getting vaccinated against COVID-19 helps stop the spread of COVID-19 and thus prevents the most vulnerable from getting sick.
> By getting vaccinated against COVID-19, **you will help keep others in your community healthy**.
> Vacunarse contra el COVID-19 ayuda a detener la propagación del COVID-19 y

así evita que los más vulnerables se enfermen.
Si usted se vacuna contra el COVID-19, **ayudará a mantener saludables a otros en su comunidad**.

*[Economic recovery]* The faster [COUNTRY] can stop the spread of COVID-19, the faster people will get back to work.
If you get vaccinated against COVID-19, **you will help the economy recover**.
Cuanto más rápido [COUNTRY] pueda detener la propagación de COVID-19, más rápido las personas volverán a trabajar.
Si usted se vacuna contra el COVID-19, **ayudará a que la economía se recupere**.

*[Social approval]* Getting vaccinated against COVID-19 shows that you care about others in your community.
If you get vaccinated against COVID-19, **you will be respected by the people in your community**.
Vacunarse contra el COVID-19 demuestra que usted se preocupa por los demás en su comunidad.
Si usted se vacuna contra el COVID-19, **será respetado por las personas en su comunidad**.

As shown in Table S2, these motivational treatments were cross-randomized with respect to the vaccine information with equal probability.
