# Supplementary material for: Messages that increase COVID-19 vaccine acceptance: Evidence from online experiments in six Latin American countries
Source: PLoS One. 2021 Oct 28;16(10):e0259059. doi: 10.1371/journal.pone.0259059 (PMC8553119; doi:10.1371/journal.pone.0259059)
Supplement: S3 Appendix — (PDF) [file pone.0259059.s003.pdf]

S3 Experimental design and estimation strategies

Treatments and randomization

The full text for each treatment condition is provided in S2 Appendix. Both the information and motivational treatments were assigned within 144 blocks defined by country (6 possible values), pre-treatment vaccine willingness (6 possible values), and age category (4 possible values). Within each block, sequential complete randomization was used to assign treatments within Qualtrics. Table S3 reports the realized distribution of treatment assignments. The corresponding treatment assignment probabilities are reported in S2 Appendix.

		**Information about vaccines?**									
								Vaccine + Herd + Current			
				Vaccine + Biden	*Vaccine + Herd*						
		None	*Vaccine*		*60%*	*70%*	*80%*	*60%*	*70%*	*80%*	Pooled
**Motivational message?**	*None*	378	406	274	143	127	124	128	121	143	1,844
	Altruism	401	365	254	121	127	111	128	130	119	1,756
	Economic recovery	386	351	245	124	128	139	128	133	124	1,758
	Social approval	375	390	249	120	129	126	124	133	121	1,767
	Pooled	1,540	1,512	1,022	508	511	500	508	517	507	7,125

Table S3: Distribution of treatments assignments. The numbers in each cell indicate the number of respondents randomized into each condition (pooling across countries).

Measurement of outcome variables

The full question and set of answers for each outcome variable is described in S5 Appendix.

Weighting of data

To maximize the representativeness of the descriptive data in Fig 2 in the main article, we apply population weights based on the most recent census. In particular, we weight respondents to match the population distribution at the education (none, primary, secondary, university, other higher) $\times$ sex (male, female) $\times$ region (multiple regions that differ by country) $\times$ age category (multiple categories that differ by country) cell level within each country. To maximize statistical power, we estimate treatment effects without applying population weights; however, we report qualitatively similar, if slightly larger and less precise, effects when such weights are applied in S17 Appendix. We also demonstrate robustness to using rake weights that achieve national representativeness over the marginal distribution of each covariate in S17 Appendix.

Estimating average treatment effects of vaccine information

We estimate the effect of each of the eight vaccine information treatments separately using the following pre-specified OLS regression:

$$\begin{aligned} Y_{ic} = \alpha_{bc} + \beta Y_{ic}^{pre} &+ \tau_1 Vaccine_{ic} + \tau_2 Vaccine\ and\ Biden_{ic} \\ &+ \sum_{k=60,70,80} \tau_3^k Vaccine\ and\ Herd\ k\%_{ic} \\ &+ \sum_{k=60,70,80} \tau_4^k Vaccine\ and\ Herd\ k\%\ and\ Current_{ic} + \varepsilon_{ic}, \end{aligned} \tag{1}$$

where Y_{ic} is an outcome for respondent i in country c, α_{bc} are block $\times$ country fixed effects, Y_{ic}^{pre} is a standardized version of the pre-treatment number of months that respondent i would wait to get vaccinated once eligible, $Vaccine_{ic}$ is an indicator for the basic vaccine information provided about COVID-19 vaccines, $Vaccine\ and\ Biden_{ic}$ is an indicator for additionally being informed that Biden was vaccinated, $Vaccine\ and\ Herd\ k\%$ is an indicator for receiving the basic vaccine information and being informed that experts believe that at least $k \in \{60, 70, 80\}$ percent of individuals will need to get vaccinated to prevent the spread of COVID-19, and $Vaccine\ and\ Herd\ k\%\ and\ Current_{ic}$ indicates respondents are further informed of their country's current rate of vaccine willingness. Between the fixed effects and the lagged outcome, we adjust for baseline pre-treatment hesitancy responses and increase statistical power. All observations are weighted by the inverse probability of treatment assignment and heteroskedasticity-robust standard errors are used in all regression analyses. Each τ coefficient estimates an average treatment effect of the corresponding treatment.

When pooling across information treatments, we estimate the following pre-specified OLS regression:

$$Y_{ic} = \alpha_{bc} + \beta Y_{ic}^{pre} + \tau Any\ vaccine\ information_{ic} + \varepsilon_{ic}, \tag{2}$$

where $Any\ vaccine\ information_{ic}$ indicates that respondent i received any information treatment and τ is the associated average treatment effect. All regression specifications were pre-specified in equivalent form or noted in the text of our pre-analysis plan, which is publicly available at the Social Science Registry (www.socialscienceregistry.org/trials/7080), unless noted otherwise.

Estimating treatment effects of belief updating about herd immunity and current aggregate willingness to vaccinate

To estimate the effect of beliefs about the level of vaccination required to achieve herd immunity, conditional on having receiving basic vaccine information, we leverage experimental variation in whether a respondent was informed that experts believe 60%, 70%, or 80% of the

population is required to achieve herd immunity. The direction of updating is not random, because this depends on a respondent's prior belief. However, conditional on a given prior belief, the direction of induced belief updating randomly varies with the expert opinion regarding the vaccination rate required to achieve herd immunity. We exploit such variation by estimating the following OLS regression among the subset of respondents that received a treatment containing information about herd immunity levels:

$$Y_{ic} = \alpha_{bc} + \beta Y_{ic}^{pre} + \tau \mathbb{1}[Herd\, prior_{ic} < k_{ic}] + \sum_{p} \eta_p \mathbb{1}[Herd\, prior_{ic} = p] + \varepsilon_{ic}, \tag{3}$$

where the treatment $\mathbb{1}[Herd\ prior_{ic} < k_{ic}]$ is an indicator for respondent i's prior belief $Herd\ prior_{ic}$ (the percentage $p \in [0, 100]$ of the population that needs to get vaccinated to stop the propagation of COVID-19, which was elicited pre-treatment) being below the reported expert opinion on the herd immunity rate k_{ic}, and τ is the associated average treatment effect. As robustness checks, we examine more fine-grained updating treatments in S10 Appendix. This approach to estimating the effect of the herd immunity level reported was not prespecified, but complements our prespecified approach comparing the effects of the conditions providing expert opinions of 60%, 70%, and 80% herd immunity requirements.

To estimate heterogeneous effects of being informed of the current level of national willingness to vaccinate with respect to a respondent's prior belief, conditional on having receiving basic vaccine information, we estimate the following OLS regression:

$$\begin{aligned} Y_{ic} = {} & \alpha_{bc} + \beta Y_{ic}^{pre} + \tau_1 Current_{ic} + \tau_2 (Current_{ic} \times \mathbb{1}[Willing\, prior_{ic} < r_{ic}]) \\ & + \eta \mathbb{1}[Willing\, prior_{ic} < r_{ic}] + \varepsilon_{ic}, \end{aligned} \tag{4}$$

where $Current_{ic}$ is an indicator for i receiving information about the current rate of vaccine willingness (where the comparison group contains control respondents and respondents that received other treatment conditions that did not report current willingness), and $\mathbb{1}[Willing\, prior_{ic} < r_{ic}]$ is an indicator for a respondent's prior belief about the willingness rate in their community being below the national willingness rate $r_{ic} \in \{56, 57, 58, 61, 64, 66, 67, 73, 75, 79\}$ reported (or that would have been reported if treated). τ_1 then estimates the effect of being informed about the current level of national vaccine willingness among respondents encouraged to update upwards about the current national rate of vaccine willingness, while $\tau_1 + \tau_2$ captures the effect of treatment among respondents encouraged to update downwards about the current national rate of vaccine willingness.

We further estimate the effect of providing information relating expert opinions on herd immunity requirements to current rates of vaccine willingness, conditional on having receiving basic vaccine information. Following our approach to estimating the effect of exposure to different expert opinions about herd immunity, whether the expert herd immunity rate opinion that a respondent received is above or below the current rate of vaccine willingness was randomly assigned, conditional on the country's current rate of willingness. Interacting this variation in potential belief updating with whether a respondent received information about the current

rate then captures the effect of learning that the current rate is above or below the expert herd immunity rate, beyond exposure to a given expert herd immunity opinion. We estimate this effect using the following OLS regression among the subset of respondents that received a herd immunity treatment:

$$Y_{ic} = \alpha_{bc} + \beta Y_{ic}^{pre} + \tau_1 Current_{ic} + \tau_2 \mathbb{1}[r_{ic} < k_{ic}] + \tau_3 (Current_{ic} \times \mathbb{1}[r_{ic} < k_{ic}]) \\ + \sum_p \eta_p \mathbb{1}[r_{ic} = p] + \sum_p \xi_p (Current_{ic} \times (\mathbb{1}[r_{ic} = p] - \mu_p)) + \varepsilon_{ic}, \quad (5)$$

where $\mathbb{1}[r_{ic} < k_{ic}]$ is an indicator for respondents for whom the expert opinion for the level of vaccination required to achieve herd immunity exceeded the current level of vaccine willingness, r_{ic}, in the respondent's country, and thus τ_1 and $\tau_1 + \tau_3$ estimate the effect of being informed that the current rate is above and below, respectively, what experts believe is required to attain herd immunity. The interactions between the (demeaned) fixed effects for the current rate at the time of the survey, $(\mathbb{1}[r_{ic} = p] - \mu_p)$ for each level of current willingness, and $Current_{ic}$ are included to identify the effect of $Current_{ic} \times \mathbb{1}[r_{ic} < k_{ic}]$; the fixed effects in the estimation sample are demeaned to ensure that τ_1 captures the conditional average treatment effect when $r_{ic} < k_{ic}$. This subtle strategy for estimating the effect of how the current willingness rate relates to the expert opinion was only recognized by the research team after conducting the experiment, and was thus not prespecified.

Estimating treatment effects of motivation messages

We estimate the effect of the three motivation messages by comparing each message to the control group receiving no message using the following pre-specified OLS regression:

$$Y_{ic} = \alpha_{bc} + \beta Y_{ic}^{pre} + \tau_1 Altruism_{ic} + \tau_2 Economic\ recovery_{ic} + \tau_3 Social\ approval_{ic} + \varepsilon_{ic}, \quad (6)$$

where $Altruism_{ic}$, $Economic\ recovery_{ic}$, and $Social\ approval_{ic}$ indicate whether respondent i received the respective treatment. Observations are unweighted due to the equal probabilities of treatment assignment. Each τ coefficient estimates an average treatment effect of the corresponding treatment.

Estimating heterogeneous treatment effects

To examine heterogeneity in the effect of the basic vaccine information treatment, we estimate OLS regressions of the following form:

$$Y_{ic} = \alpha_{bc} + \beta Y_{ic}^{pre} + \tau_0 Any\ vaccine\ information_{ic} \\ + \boldsymbol{\tau}_1 (Any\ vaccine\ information_{ic} \times \mathbf{X}_{ic}) + \boldsymbol{\gamma} \mathbf{X}_{ic} + \varepsilon_{ic}, \quad (7)$$

where $\mathbf{X}_{ic}$ is a vector of predetermined respondent-level characteristics. To estimate heterogeneity in the effect of the motivational treatments, we estimate analogous equations where we replace $Any\ vaccine\ information_{ic}$ with indicators for the three motivational messages.

Statistical inference

All statistical inferences are derived from two-tailed t tests and 95% confidence intervals based on the regressions previously described. The two-tailed tests are more conservative than the one-tailed tests for positive average treatment effects than we pre-specified.

Computing persuasion rates

Following standard practice in the information and persuasion literature [1], we compute the persuasion rate as: $100 \times \frac{ATE}{1-Y_0}$, where ATE is a given average treatment effect of interest and Y_0 is the (post-treatment) control group mean outcome. The persuasion rate captures the share of the non-willing that become willing due to treatment. Since all treated respondents were directly exposed to treatment, we do not adjust for the share of respondents that engaged with treatment.

Support for the identifying assumptions

The average treatment effects are identified under two assumptions: (i) the stable unit treatment value assumption (SUTVA); and (ii) unconfounded treatment assignment. SUTVA almost certainly holds because interference between respondents between start and end of the survey is implausible in the large countries under study and because versions of treatment were controlled by the research team. Although treatments were randomly assigned, identification of causal effects could be confounded by chance imbalances or differential attrition across treatment groups. As S7 Appendix shows, neither potential concern drives the results and the results are robust to bounding our estimates to address differences in attrition [2]. The identification conditions for conditional average treatment effects are analogous within subgroups.

Implementation of statistical analyses

All statistical analyses were implemented in R, with the exception of initial data cleaning and implementation of the bounding exercises that were conducted in Stata.
