# Supplementary material for: Messages that increase COVID-19 vaccine acceptance: Evidence from online experiments in six Latin American countries
Source: PLoS One. 2021 Oct 28;16(10):e0259059. doi: 10.1371/journal.pone.0259059 (PMC8553119; doi:10.1371/journal.pone.0259059)
Supplement: S4 Appendix — (PDF) [file pone.0259059.s004.pdf]

S4 Manipulation checks

To test whether the vaccine information was internalized by treated respondents, we asked two questions later in the survey about the basic vaccine information received by all treated respondents. This information was not included in the comprehension quiz that appeared with each component of the treatment information. In particular, respondents were asked whether vaccines had yet been approved in some countries and whether there were only minimal side effects of the vaccines. The results in Table S4 show that respondents receiving any vaccine information were almost 0.05 probability points more likely to answer the first question correctly, relative to an already high share of respondents in the control group that answered correctly (0.78), and 0.11 probability points more likely to answer the second more difficult question correctly. Although there was some heterogeneity by specific information treatment (even though all treated respondents received the information relating to the questions), all conditions significantly increased vaccine knowledge. The smaller effects associated with the treatments including information about the current level of willingness in a respondent's country suggests a possibility for information overload.

	Outcome variables:	
	Know that vaccines were approved	Know that there are minimal side effects
**Panel A: Pooled across vaccine information treatments**		
Any vaccine information	0.044***	0.110***
	(0.011)	(0.014)
Outcome range	{0,1}	{0,1}
Control outcome mean	0.78	0.45
Control outcome std. dev.	0.42	0.50
Observations	7,033	7,019
R^2	0.078	0.095
**Panel B: By vaccine information treatment condition**		
Vaccine	0.035**	0.091***
	(0.015)	(0.018)
Vaccine + Biden	0.034**	0.084***
	(0.016)	(0.020)
Vaccine + Herd 60%	0.085***	0.137***
	(0.019)	(0.025)
Vaccine + Herd 70%	0.078***	0.143***
	(0.019)	(0.025)
Vaccine + Herd 80%	0.084***	0.153***
	(0.019)	(0.025)
Vaccine + Herd 60% + Current	0.016	0.151***
	(0.021)	(0.025)
Vaccine + Herd 70% + Current	0.030	0.094***
	(0.020)	(0.025)
Vaccine + Herd 80% + Current	0.033	0.095***
	(0.020)	(0.025)
Outcome range	{0,1}	{0,1}
Control outcome mean	0.78	0.45
Control outcome std. dev.	0.42	0.50
Observations	7,033	7,019
R^2	0.074	0.103

Table S4: Vaccine information comprehension tests. All specifications include country $\times$ block fixed effects and (standardized) pre-treatment wait until vaccination as covariates (omitted to save space), weight observations by the inverse probability of treatment assignment, and are estimated using OLS. Robust standard errors are in parentheses. * denotes $p < 0.1$, ** denotes $p < 0.05$, *** denotes $p < 0.01$ from two-sided t tests.
