# Supplementary material for: Messages that increase COVID-19 vaccine acceptance: Evidence from online experiments in six Latin American countries
Source: PLoS One. 2021 Oct 28;16(10):e0259059. doi: 10.1371/journal.pone.0259059 (PMC8553119; doi:10.1371/journal.pone.0259059)
Supplement: S5 Appendix — (PDF) [file pone.0259059.s005.pdf]

S5 Measurement of outcome variables

We focus on four primary outcome variables capturing intention to vaccinate:

1. *Vaccine willingness scale*: a five-point scale ranging from "strongly disagree" (1) to "strongly agree" (5) capturing a respondent's willingness to get vaccinated if a vaccine were available. The specific question was: "To what extent do you agree or disagree? If a vaccine against COVID-19 were available, I would get vaccinated. Strongly disagree? Disagree? Neither agree nor disagree? Agree? Strongly agree? Not sure?" In Spanish, this read as: "¿Hasta qué punto está usted de acuerdo o en desacuerdo? Si una vacuna contra el COVID-19 estuviera disponible, yo me vacunaría. Muy en desacuerdo? En desacuerdo? Ni de acuerdo ni en disacuerdo? De acuerdo? Muy de acuerdo? No estoy seguro?"

2. *Willing to take a vaccine*: an indicator coded one for respondents that answered "agree" or "strongly agree" to the previous question.

3. *Months would wait to get vaccinated*: number of months, top-coded at 12, that a respondent would wait to get vaccinated if a COVID-19 vaccine were available to you now. The specific question was: "If a vaccine against COVID-19 were available to you now, how many months would you wait before get vaccinated? Number of month: ...? I would not take a vaccine?" In Spanish, this read as: "Si una vacuna contra el COVID-19 estuviera disponible para usted ahora, ¿cuántos meses esperaría antes de vacunarse? Numero de meses: ...? Nunca tomaría una vacuna?"

4. *Encourage others to get vaccinated*: an indicate coded 1 for respondents that responded "somewhat likely" or "very likely" to the following question: "How likely are you to encourage family or friends to get vaccinated? Never? Unlikely? Somewhat likely? Very likely?" In Spanish, this read as: "¿Qué tan probable es que motive a familiares o amigos a que se vacunen? Nada probable? Poco probable? Algo probable? Muy probable?"

(Portuguese translations are available upon request.) These questions appeared a few screens after the motivation treatments were delivered. Identical versions of the first three variables were elicited at the beginning of the survey to determine whether a respondent would be screened based on already being willing to take a vaccine (see S1 Appendix for more information about screening). While the first three outcomes were pre-specified as primary outcomes, we included the encourage others outcome in light of the positive effect and the high salience of social dynamics in our findings.

Since we could not measure actual vaccination because vaccine rollouts in Latin America were limited at the time of the study, we tried to measure vaccine willingness behaviorally by assessing whether respondents choose to receive additional information about COVID-19 vaccines from the Pan American Health Organization (PAHO) and ultimately clicked through to their website. To measure the latter, we wrote code to verify whether the link on the Qualtrics

page was clicked. These variables provide behavioral measures of interest in obtaining further information about COVID-19 vaccines. However, this may only imperfectly correlate with vaccine willingness intentions because further information may not be required to convince individuals after treatment. (At the time of the study, government websites did not have online sign-up portals that would have represented a more direct behavioral measure of interest in taking a vaccine.) Accordingly, we do not focus on this outcome in our main analysis; since it was pre-specified, we report the results for whether a respondent requested to receive the link and actually clicked through below in S16 Appendix.
