# Supplementary material for: Messages that increase COVID-19 vaccine acceptance: Evidence from online experiments in six Latin American countries
Source: PLoS One. 2021 Oct 28;16(10):e0259059. doi: 10.1371/journal.pone.0259059 (PMC8553119; doi:10.1371/journal.pone.0259059)
Supplement: S6 Appendix — (PDF) [file pone.0259059.s006.pdf]

S6 The main results in regression table form

Tables S5-S8 report the regression estimates that underlie Figs 4 - 7.

	Outcome variable:			
	Vaccine willingness scale	Willing to take a vaccine	Months would wait to get vaccinated (reversed)	Encourage others to get vaccinated
	(1)	(2)	(3)	(4)
**Panel A: All countries pooled**				
Any vaccine information	0.143***	0.046***	0.410***	0.037***
	(0.024)	(0.010)	(0.058)	(0.012)
Outcome range	[1,5]	{0,1}	[0,12]	{0,1}
Control outcome mean	3.17	0.40	5.78	0.54
Control outcome std. dev.	1.18	0.49	4.38	0.50
Observations	6,951	6,951	6,876	6,659
R^2	0.483	0.492	0.766	0.356
**Panel B: Argentina**				
Any vaccine information	0.172***	0.043*	0.449***	0.050*
	(0.062)	(0.025)	(0.131)	(0.029)
Outcome range	[1,5]	{0,1}	[0,12]	{0,1}
Control outcome mean	3.02	0.36	5.11	0.47
Control outcome std. dev.	1.18	0.48	4.45	0.50
Observations	1,160	1,160	1,150	1,109
R^2	0.442	0.462	0.801	0.351
**Panel C: Brazil**				
Any vaccine information	0.200***	0.081***	0.344**	0.027
	(0.052)	(0.022)	(0.148)	(0.028)
Outcome range	[1,5]	{0,1}	[0,12]	{0,1}
Control outcome mean	3.30	0.42	5.92	0.49
Control outcome std. dev.	1.18	0.49	4.42	0.50
Observations	1,213	1,213	1,187	1,134
R^2	0.603	0.576	0.730	0.400
**Panel D: Chile**				
Any vaccine information	0.177***	0.070***	0.392***	0.068**
	(0.060)	(0.024)	(0.128)	(0.030)
Outcome range	[1,5]	{0,1}	[0,12]	{0,1}
Control outcome mean	2.89	0.31	4.80	0.46
Control outcome std. dev.	1.23	0.46	4.39	0.50
Observations	1,114	1,114	1,106	1,080
R^2	0.511	0.501	0.810	0.351
**Panel E: Colombia**				
Any vaccine information	0.187***	0.074***	0.326***	0.067**
	(0.059)	(0.024)	(0.119)	(0.028)
Outcome range	[1,5]	{0,1}	[0,12]	{0,1}
Control outcome mean	3.18	0.39	6.08	0.57
Control outcome std. dev.	1.16	0.49	4.18	0.50
Observations	1,131	1,131	1,120	1,085
R^2	0.460	0.484	0.819	0.378
**Panel F: México**				
Any vaccine information	0.054	0.002	0.507***	0.009
	(0.065)	(0.026)	(0.155)	(0.028)
Outcome range	[1,5]	{0,1}	[0,12]	{0,1}
Control outcome mean	3.49	0.54	6.77	0.69
Control outcome std. dev.	1.21	0.50	4.26	0.46
Observations	1,102	1,102	1,098	1,075
R^2	0.415	0.477	0.717	0.311
**Panel G: Perú**				
Any vaccine information	0.061	0.004	0.417**	0.004
	(0.055)	(0.026)	(0.169)	(0.029)
Outcome range	[1,5]	{0,1}	[0,12]	{0,1}
Control outcome mean	3.14	0.39	5.99	0.57
Control outcome std. dev.	1.04	0.49	4.31	0.49
Observations	1,231	1,231	1,215	1,176
R^2	0.404	0.411	0.702	0.296

Table S5: Effect of any vaccine information on vaccine willingness. All specifications include country $\times$ block fixed effects and (standardized) pre-treatment wait until vaccination as covariates (omitted to save space), weight observations by the inverse probability of treatment assignment, and are estimated using OLS. Robust standard errors are in parentheses. * denotes $p < 0.1$, ** denotes $p < 0.05$, *** denotes $p < 0.01$ from two-sided t tests.

	Outcome variable:			
	Vaccine willingness scale	Willing to take a vaccine	Months would wait to get vaccinated (reversed)	Encourage others to get vaccinated
	(1)	(2)	(3)	(4)
Vaccine	0.148***	0.045***	0.346***	0.047***
	(0.032)	(0.013)	(0.083)	(0.016)
Vaccine + Biden	0.121***	0.039***	0.377***	0.018
	(0.037)	(0.015)	(0.095)	(0.018)
Vaccine + Herd 60%	0.092**	0.036*	0.410***	0.029
	(0.046)	(0.019)	(0.121)	(0.022)
Vaccine + Herd 70%	0.187***	0.051***	0.531***	0.042*
	(0.047)	(0.020)	(0.120)	(0.022)
Vaccine + Herd 80%	0.131***	0.043**	0.347***	0.011
	(0.045)	(0.019)	(0.126)	(0.022)
Vaccine + Herd 60% + Current	0.183***	0.081***	0.520***	0.079***
	(0.046)	(0.020)	(0.126)	(0.022)
Vaccine + Herd 70% + Current	0.183***	0.067***	0.408***	0.064***
	(0.046)	(0.020)	(0.119)	(0.022)
Vaccine + Herd 80% + Current	0.102**	0.010	0.510***	0.010
	(0.049)	(0.020)	(0.131)	(0.022)
Outcome range	[1,5]	{0,1}	[0,12]	{0,1}
Control outcome mean	3.17	0.40	5.78	0.54
Control outcome std. dev.	1.18	0.49	4.38	0.50
Observations	6,951	6,951	6,876	6,659
R^2	0.433	0.442	0.716	0.339

Table S6: Effect of different types of vaccine information on vaccine willingness. All specifications include country $\times$ block fixed effects and (standardized) pre-treatment wait until vaccination as covariates (omitted to save space), weight observations by the inverse probability of treatment assignment, and are estimated using OLS. Robust standard errors are in parentheses. * denotes $p < 0.1$, ** denotes $p < 0.05$, *** denotes $p < 0.01$ from two-sided t tests.

	Outcome variable:			
	Vaccine willingness scale	Willing to take a vaccine	Months would wait to get vaccinated (reversed)	Encourage others to get vaccinated
	(1)	(2)	(3)	(4)
Current	0.140^{**}	0.079^{***}	0.105	0.076^{***}
	(0.064)	(0.027)	(0.166)	(0.029)
Current rate below herd opinion	0.088	0.027	0.047	0.024
	(0.057)	(0.023)	(0.146)	(0.027)
Current × Current rate below herd opinion	-0.185^{**}	-0.104^{***}	-0.115	-0.084^{**}
	(0.083)	(0.034)	(0.214)	(0.037)
Outcome range	[1,5]	{0,1}	[0,12]	{0,1}
Control outcome mean	3.30	0.45	6.04	0.53
Control outcome std. dev.	1.20	0.50	4.49	0.50
Observations	2,955	2,955	2,919	2,821
R^2	0.441	0.444	0.712	0.364

Table S7: The effect of being informed that the current rate of vaccination willingness in the population is above/below the rate required for herd immunity. All specifications include country × block fixed effects and (standardized) pre-treatment wait until vaccination as covariates (omitted to save space) and are estimated using OLS. Robust standard errors are in parentheses. * denotes $p < 0.1$, ** denotes $p < 0.05$, *** denotes $p < 0.01$ from two-sided t tests.

	Outcome variable: Vaccine willingness scale (1)	Willing to take a vaccine (2)	Months would wait to get vaccinated (reversed) (3)	Encourage others to get vaccinated (4)
**Panel A: All countries pooled**				
Altruism	0.022	0.014	0.074	0.018
	(0.030)	(0.013)	(0.080)	(0.014)
Economic recovery	0.051*	0.021*	−0.011	0.030**
	(0.030)	(0.013)	(0.080)	(0.014)
Social approval	0.105***	0.046***	0.252***	0.042***
	(0.030)	(0.013)	(0.084)	(0.014)
Outcome range	[1,5]	{0,1}	[0,12]	{0,1}
Control outcome mean	3.24	0.42	6.07	0.55
Control outcome std. dev.	1.17	0.49	4.41	0.50
Observations	6,951	6,951	6,876	6,659
R^2	0.442	0.456	0.728	0.337
**Panel B: Argentina**				
Altruism	0.004	−0.016	0.251	0.017
	(0.073)	(0.031)	(0.185)	(0.036)
Economic recovery	0.115*	0.034	0.004	0.005
	(0.069)	(0.031)	(0.180)	(0.035)
Social approval	0.076	0.038	0.244	0.013
	(0.076)	(0.033)	(0.178)	(0.037)
Outcome range	[1,5]	{0,1}	[0,12]	{0,1}
Control outcome mean	3.14	0.40	5.76	0.52
Control outcome std. dev.	1.11	0.49	4.40	0.50
Observations	1,160	1,160	1,150	1,109
R^2	0.417	0.441	0.773	0.330
**Panel C: Brazil**				
Altruism	−0.052	−0.004	0.112	−0.017
	(0.063)	(0.027)	(0.214)	(0.033)
Economic recovery	0.024	0.019	0.435**	0.035
	(0.063)	(0.028)	(0.196)	(0.033)
Social approval	0.110*	0.051*	0.633***	0.028
	(0.060)	(0.027)	(0.208)	(0.034)
Outcome range	[1,5]	{0,1}	[0,12]	{0,1}
Control outcome mean	3.41	0.46	5.97	0.49
Control outcome std. dev.	1.19	0.50	4.45	0.50
Observations	1,213	1,213	1,187	1,134
R^2	0.580	0.546	0.683	0.387
**Panel D: Chile**				
Altruism	0.164**	0.086***	0.061	0.042
	(0.080)	(0.030)	(0.172)	(0.036)
Economic recovery	0.145*	0.072**	0.153	0.069*
	(0.079)	(0.030)	(0.191)	(0.035)
Social approval	0.263***	0.126***	0.408**	0.076**
	(0.079)	(0.030)	(0.197)	(0.036)
Outcome range	[1,5]	{0,1}	[0,12]	{0,1}
Control outcome mean	2.92	0.31	5.17	0.49
Control outcome std. dev.	1.22	0.46	4.53	0.50
Observations	1,114	1,114	1,106	1,080
R^2	0.463	0.472	0.760	0.330
**Panel E: Colombia**				
Altruism	0.035	0.032	0.666***	0.077**
	(0.078)	(0.032)	(0.167)	(0.034)
Economic recovery	0.017	0.012	0.138	0.042
	(0.076)	(0.032)	(0.172)	(0.034)
Social approval	0.117	0.045	0.256	0.087**
	(0.075)	(0.031)	(0.179)	(0.035)
Outcome range	[1,5]	{0,1}	[0,12]	{0,1}
Control outcome mean	3.26	0.41	5.96	0.57
Control outcome std. dev.	1.16	0.49	4.47	0.50
Observations	1,131	1,131	1,120	1,085
R^2	0.424	0.449	0.784	0.343
**Panel F: México**				
Altruism	−0.007	−0.001	−0.121	0.045
	(0.082)	(0.033)	(0.212)	(0.035)
Economic recovery	−0.004	−0.002	−0.245	0.045
	(0.090)	(0.034)	(0.225)	(0.035)
Social approval	0.035	0.006	−0.015	0.047
	(0.083)	(0.033)	(0.226)	(0.036)
Outcome range	[1,5]	{0,1}	[0,12]	{0,1}
Control outcome mean	3.54	0.55	7.23	0.66
Control outcome std. dev.	1.16	0.50	4.02	0.48
Observations	1,102	1,102	1,098	1,075
R^2	0.349	0.422	0.673	0.293
**Panel G: Perú**				
Altruism	−0.001	−0.006	−0.486**	−0.050
	(0.071)	(0.033)	(0.214)	(0.035)
Economic recovery	0.023	−0.004	−0.494**	−0.012
	(0.069)	(0.032)	(0.199)	(0.036)
Social approval	0.043	0.014	0.006	0.009
	(0.072)	(0.033)	(0.227)	(0.035)
Outcome range	[1,5]	{0,1}	[0,12]	{0,1}
Control outcome mean	3.16	0.39	6.35	0.58
Control outcome std. dev.	1.08	0.49	4.34	0.49
Observations	1,231	1,231	1,215	1,176
R^2	0.360	0.369	0.679	0.291

Table S8: Effect of different types of motivational message on vaccine willingness. All specifications include country $\times$ block fixed effects and (standardized) pre-treatment wait until vaccination as covariates (omitted to save space) and are estimated using OLS. Robust standard errors are in parentheses. * denotes $p < 0.1$, ** denotes $p < 0.05$, *** denotes $p < 0.01$ from two-sided t tests.
