# Supplementary material for: Messages that increase COVID-19 vaccine acceptance: Evidence from online experiments in six Latin American countries
Source: PLoS One. 2021 Oct 28;16(10):e0259059. doi: 10.1371/journal.pone.0259059 (PMC8553119; doi:10.1371/journal.pone.0259059)
Supplement: S7 Appendix — (PDF) [file pone.0259059.s007.pdf]

S7 Identification checks

As noted in S3 Appendix, our estimation of treatment effects relies on two assumptions: SUTVA and unconfounded treatment assignment. While SUTVA almost certainly holds in our context of online surveys where around 1,000 individuals were randomly assigned treatments in each country, there remains a risk that the random assignment of treatments could be broken by differences in attrition—that is to say in the likelihood of continuing the survey to answer post-treatment outcomes across—across experimental groups. We examine differences in attrition between treatment groups and the control group by using our main regression specifications to examine whether treatments differentially affected the probability of answering post-treatment outcome questions.

Table S9 reports the results for receiving any vaccine information. Panel A pools across countries and indicates that respondents that received any vaccine information were around 2 percentage points less likely to answer our main outcome questions. Panels B-G indicate that this difference is driven primarily by respondents in Colombia and Perú. Within the pooled sample, the difference in answering our three main outcome questions between treated and control respondents is statistically significant in each case, although the difference is relatively small in magnitude. Among the treated respondents, we find no evidence of differential attrition between treatment arms: for each of our three main outcomes, we fail to reject the null hypothesis that the response rate is identical across the eight different treatment groups ($p = 0.47$, $p = 0.40$, and $p = 0.64$, respectively).

We next turn to attrition for the motivational message treatments reported in Table S10. Focusing again on the estimates that pool across countries in panel A, we observe more substantial differences in attrition between the message and control groups: for each message, the probability of answering the post-treatment questions is around 5 percentage points higher. Again, we fail to reject the null hypothesis that there is no difference in attrition between each type of message treatment ($p = 0.42$).

These differences raise the concern that the estimates could be biased if certain types of respondent are more likely to attrite when they receive certain treatment conditions. To gauge whether such differential attrition is likely to bias our estimates, we first examine balance across pre-treatment covariates before and after respondents had the opportunity to attrite. Column (1) of Tables S11 and S12 examines balance at the point of assignment—before attrition could kick in. Consistent with the integrity of the randomized assignment of treatment, differences between treatment and control groups are consistent with chance: of 81 pre-treatment covariates, we reject at the 10% level the null hypothesis that the mean in each experimental (treatment or control) group is equal in only 4 cases for the vaccine information treatments and in 14 cases for the motivation treatments. Columns (2)-(4) next examine how differences in pre-treatment covariates change once attrition by the time that different outcome variables are reached is accounted for. If differences in attrition across experimental groups break the randomization because attrition did not occur at random within groups, we should expect differences to emerge at this point. However, the results indicate that significant imbalances do not arise due to attrition:

	Outcome variable:		
	Answered vaccine willingness scale	Answered wait until vaccination	Answered encourage others to get vaccinated
	(1)	(2)	(3)
**Panel A: All countries pooled**			
Any vaccine information	-0.017^{***}	-0.017^{***}	-0.023^{***}
	(0.003)	(0.004)	(0.006)
Outcome range	{0,1}	{0,1}	{0,1}
Control outcome mean	0.99	0.98	0.95
Control outcome std. dev.	0.10	0.15	0.21
Observations	7,125	7,125	7,125
R^2	0.032	0.040	0.046
**Panel B: Argentina**			
Any vaccine information	-0.002	-0.003	-0.017
	(0.010)	(0.012)	(0.016)
Outcome range	{0,1}	{0,1}	{0,1}
Control outcome mean	0.98	0.97	0.95
Control outcome std. dev.	0.14	0.16	0.22
Observations	1,184	1,184	1,184
R^2	0.025	0.021	0.029
**Panel C: Brazil**			
Any vaccine information	-0.023^{***}	-0.012	-0.011
	(0.008)	(0.014)	(0.019)
Outcome range	{0,1}	{0,1}	{0,1}
Control outcome mean	0.99	0.96	0.92
Control outcome std. dev.	0.10	0.20	0.28
Observations	1,248	1,248	1,248
R^2	0.033	0.042	0.040
**Panel D: Chile**			
Any vaccine information	-0.019^{**}	-0.013	-0.015
	(0.010)	(0.012)	(0.016)
Outcome range	{0,1}	{0,1}	{0,1}
Control outcome mean	0.98	0.97	0.95
Control outcome std. dev.	0.13	0.17	0.22
Observations	1,149	1,149	1,149
R^2	0.031	0.048	0.036
**Panel E: Colombia**			
Any vaccine information	-0.019^{***}	-0.026^{***}	-0.033^{**}
	(0.007)	(0.009)	(0.014)
Outcome range	{0,1}	{0,1}	{0,1}
Control outcome mean	1.00	0.99	0.97
Control outcome std. dev.	0.06	0.09	0.18
Observations	1,154	1,154	1,154
R^2	0.030	0.029	0.041
**Panel F: México**			
Any vaccine information	-0.008	-0.013^{**}	-0.017
	(0.006)	(0.007)	(0.011)
Outcome range	{0,1}	{0,1}	{0,1}
Control outcome mean	0.99	0.99	0.98
Control outcome std. dev.	0.09	0.09	0.16
Observations	1,119	1,119	1,119
R^2	0.053	0.047	0.055
**Panel G: Perú**			
Any vaccine information	-0.030^{***}	-0.032^{***}	-0.044^{***}
	(0.008)	(0.011)	(0.014)
Outcome range	{0,1}	{0,1}	{0,1}
Control outcome mean	0.99	0.98	0.96
Control outcome std. dev.	0.09	0.13	0.20
Observations	1,271	1,271	1,271
R^2	0.030	0.039	0.059

Table S9: Effect of receiving any vaccination information on responding to main post-treatment outcome questions. All specifications include country × block fixed effects and (standardized) pre-treatment wait until vaccination as covariates (omitted to save space), weight observations by the inverse probability of treatment assignment, and are estimated using OLS. Robust standard errors are in parentheses. * denotes $p < 0.1$, ** denotes $p < 0.05$, *** denotes $p < 0.01$ from two-sided t tests.

	Outcome variable:		
	Answered vaccine willingness scale	Answered wait until vaccination	Answered encourage others to get vaccinated
	(1)	(2)	(3)
**Panel A: All countries pooled**			
Altruism	0.050***	0.052***	0.052***
	(0.006)	(0.007)	(0.009)
Economic recovery	0.045***	0.046***	0.047***
	(0.006)	(0.007)	(0.009)
Social approval	0.049***	0.052***	0.052***
	(0.006)	(0.007)	(0.009)
Outcome range	{0,1}	{0,1}	{0,1}
Control outcome mean	0.94	0.93	0.90
Control outcome std. dev.	0.24	0.26	0.30
Observations	7,125	7,125	7,125
R^2	0.046	0.043	0.039
**Panel B: Argentina**			
Altruism	0.041***	0.043**	0.044**
	(0.015)	(0.017)	(0.022)
Economic recovery	0.044***	0.053***	0.041*
	(0.014)	(0.016)	(0.023)
Social approval	0.052***	0.058***	0.066***
	(0.014)	(0.016)	(0.021)
Outcome range	{0,1}	{0,1}	{0,1}
Control outcome mean	0.95	0.93	0.90
Control outcome std. dev.	0.22	0.25	0.30
Observations	1,184	1,184	1,184
R^2	0.043	0.037	0.039
**Panel C: Brazil**			
Altruism	0.048***	0.039**	0.023
	(0.015)	(0.018)	(0.024)
Economic recovery	0.036**	0.017	0.022
	(0.016)	(0.020)	(0.024)
Social approval	0.048***	0.044**	0.032
	(0.015)	(0.018)	(0.023)
Outcome range	{0,1}	{0,1}	{0,1}
Control outcome mean	0.94	0.93	0.89
Control outcome std. dev.	0.24	0.26	0.32
Observations	1,248	1,248	1,248
R^2	0.038	0.034	0.024
**Panel D: Chile**			
Altruism	0.056***	0.062***	0.057***
	(0.016)	(0.017)	(0.020)
Economic recovery	0.058***	0.053***	0.044**
	(0.016)	(0.018)	(0.021)
Social approval	0.044**	0.054***	0.048**
	(0.017)	(0.018)	(0.021)
Outcome range	{0,1}	{0,1}	{0,1}
Control outcome mean	0.93	0.92	0.90
Control outcome std. dev.	0.26	0.27	0.30
Observations	1,149	1,149	1,149
R^2	0.046	0.050	0.031
**Panel E: Colombia**			
Altruism	0.048***	0.067***	0.069***
	(0.013)	(0.016)	(0.021)
Economic recovery	0.038***	0.056***	0.057***
	(0.014)	(0.017)	(0.022)
Social approval	0.044***	0.055***	0.060***
	(0.014)	(0.017)	(0.022)
Outcome range	{0,1}	{0,1}	{0,1}
Control outcome mean	0.95	0.93	0.89
Control outcome std. dev.	0.23	0.26	0.31
Observations	1,154	1,154	1,154
R^2	0.050	0.050	0.035
**Panel F: México**			
Altruism	0.040***	0.043***	0.057***
	(0.013)	(0.013)	(0.017)
Economic recovery	0.038***	0.034**	0.050***
	(0.013)	(0.015)	(0.018)
Social approval	0.041***	0.040***	0.038**
	(0.013)	(0.014)	(0.019)
Outcome range	{0,1}	{0,1}	{0,1}
Control outcome mean	0.95	0.95	0.92
Control outcome std. dev.	0.21	0.22	0.27
Observations	1,119	1,119	1,119
R^2	0.058	0.050	0.062
**Panel G: Perú**			
Altruism	0.063***	0.056***	0.064***
	(0.016)	(0.019)	(0.022)
Economic recovery	0.055***	0.061***	0.068***
	(0.016)	(0.018)	(0.022)
Social approval	0.061***	0.061***	0.069***
	(0.017)	(0.019)	(0.023)
Outcome range	{0,1}	{0,1}	{0,1}
Control outcome mean	0.92	0.91	0.88
Control outcome std. dev.	0.26	0.28	0.33
Observations	1,271	1,271	1,271
R^2	0.048	0.037	0.046

Table S10: Effect of motivational messages on responding to main post-treatment outcome questions. All specifications include country $\times$ block fixed effects and (standardized) pre-treatment wait until vaccination as covariates (omitted to save space) and are estimated using OLS. Robust standard errors are in parentheses. * denotes $p < 0.1$, ** denotes $p < 0.05$, *** denotes $p < 0.01$ from two-sided t tests.

we again observe only 4 instances where we can reject the null hypothesis of equality across experimental groups in the case of the vaccine information treatments; while there is some variation across outcome variables for the motivational messages, the overall number of imbalances is again similar in the datasets with and without attrition. In sum, this evidence suggests that the individuals that differentially attrited in certain experimental groups are not systematically different from those that did not.

Nevertheless, it remains possible that the respondents that attrited upon receiving a specific treatment condition could differ in terms of unobserved characteristics that might influence potential outcomes. To address this concerns, our second approach uses the non-parametric bounding approach proposed Lee [1] to examine how our estimates change in the case of severe forms of selection into responding to post-treatment questions. When attrition is greater in the treatment group than the comparison group, the upper (lower) bound on the treatment effect is obtained by trimming the most extreme values from the lower (upper) tail of the outcome distribution in the treatment group until the groups are of equal size (adjusting for probability of treatment assignment); the reverse holds when attrition is greater in the comparison group. This procedure, which does not rely on statistical assumptions, allows the researcher to compute a 95% confidence interval for the treatment effect that captures both uncertainty due to random assignment as well as uncertainty due to the potential selection bias induced by attrition. To implement this bounding approach, we focus on unadjusted comparisons between treatment and control groups (with inverse probability of treatment assignment weights), which exclude the fixed effects used to increase the precision of our estimates because analytic standard errors could not be obtained. Due to our randomization, the exclusion of such fixed effects does not induce bias.

Tables S13 and S14 report the 95% confidence intervals for the bounds on the effects of any vaccine treatment and the different motivational treatments in the sample that pools across countries. We do not report results for differences between information treatments (i.e. the results corresponding to Tables S6 and S7) because there is no evidence of differential between information treatments (see above). Given the limited levels of differential attrition, the confidence interval for receiving any vaccine information unsurprisingly show that the Lee bounds are relatively tight: for each estimate, the 95% confidence interval is only slightly larger than for our main estimates, and the lower bound remains statistically significantly different from zero in each case. Consequently, differences in attrition cannot account for the positive effects of basic vaccine information on vaccine willingness.

Turning to the motivational messages in Table S14, the 95% confidence intervals for the treatment effects of each message are larger due to the greater differences in attrition between the control and message groups. Panels A-C examine each motivational message separately relative to the control group, given that Lee bounds cannot be computed for multiple treatments simultaneously. The results for the social approval message show that the lower bound includes effects that are statistically indistinguishable from zero, although the upper bound equally includes effects that are much larger than our main estimates suggest. While differential attrition increases uncertainty about the exact effect of the social approval message, there are two impor-

Pre-treatment covariate	**Sample for which balance is tested**: Received treatment (1)	Answered vaccine willingness scale (2)	Answered wait until vaccination (3)	Answered encourage others to get vaccinated (4)
Education - None	0.603	0.529	0.649	0.662
Education - Primary	0.683	0.783	0.754	0.77
Education - Secondary	0.366	0.387	0.515	0.543
Education - Other Higher	0.378	0.33	0.416	0.397
Education - University	0.124	0.21	0.239	0.272
Gender	0.386	0.42	0.358	0.437
Running Water in Home	0.72	0.837	0.923	0.839
Sewage in Home	0.544	0.507	0.505	0.631
Electricity in Home	0.202	0.261	0.359	0.214
No Running Water, Sewage, or Electricity in Home	0.824	0.741	0.772	0.345
COVID News Consumption - TV	0.462	0.357	0.409	0.35
COVID News Consumption - Radio	0.736	0.683	0.733	0.532
COVID News Consumption - Print	0.529	0.493	0.556	0.691
COVID News Consumption - Word of Mouth	0.942	0.912	0.905	0.885
COVID News Consumption - WhatsApp	0.525	0.761	0.771	0.762
COVID News Consumption - Social Media	0.812	0.829	0.806	0.846
COVID News Consumption - News Websites	0.627	0.494	0.437	0.284
COVID Severity in Country	0.468	0.533	0.599	0.601
Herd Immunity Prior	0.237	0.289	0.275	0.291
General Vaccine Hesitancy - Protect from Disease	0.704	0.808	0.83	0.814
General Vaccine Hesitancy - Good for Community	0.994	0.998	0.996	0.996
General Vaccine Hesitancy - Trust in Government	0.143	0.247	0.313	0.339
General Vaccine Hesitancy - Follow Doctor Instructions	0.725	0.713	0.665	0.593
General Vaccine Hesitancy - Trust in International Medical Experts	0.793	0.738	0.744	0.6
General Vaccine Hesitancy - Refused Vaccine	0.567	0.529	0.542	0.622
COVID Hesitancy Reasons - Side Effects	0.421	0.276	0.275	0.207
COVID Hesitancy Reasons - Vaccine Gives COVID	0.223	0.224	0.311	0.344
COVID Hesitancy Reasons - Produced Too Quickly	0.366	0.256	0.213	0.23
COVID Hesitancy Reasons - Not Effective	0.334	0.261	0.201	0.182
COVID Hesitancy Reasons - Not At Risk of Getting COVID	0.362	0.429	0.343	0.268
COVID Hesitancy Reasons - Against Vaccines Generally	0.786	0.833	0.848	0.9
COVID Hesitancy Reasons - Prefer ’Natural’ Immunity	0.197	0.243	0.305	0.232
COVID Hesitancy Reasons - Already Had COVID	0.568	0.558	0.633	0.597
COVID Hesitancy Reasons - Don’t Trust Government	0.106	0.137	0.118	0.199
COVID Hesitancy Reasons - Financial Concerns	0.484	0.528	0.587	0.658
COVID Hesitancy Reasons - Other	0.594	0.602	0.642	0.517
Comorbidities - None	0.47	0.453	0.413	0.443
Comorbidities - Diabetes	0.265	0.233	0.318	0.298
Comorbidities - Cardiovascular Diseases	0.47	0.374	0.385	0.449
Comorbidities - Obesity	0.691	0.717	0.584	0.72
Comorbidities - Autoimmune Diseases	0.795	0.779	0.8	0.803
Comorbidities - Chronic Obstructive Pulmonary Disease	0.128	0.186	0.197	0.22
Comorbidities - Prefer Not To Share	0.48	0.582	0.513	0.705
Had COVID	0.952	0.987	0.976	0.979
Know Someone Seriously Ill or Passed Away COVID	0.325	0.342	0.414	0.567
COVID Economic Situation	0.337	0.425	0.446	0.228
Government Vaccine Priority	0.791	0.793	0.834	0.824
Left/Right Political Scale	0.262	0.188	0.145	0.102
Satisfied with President COVID Management	0.305	0.334	0.466	0.546
Satisfied with Mayor COVID Management	0.017**	0.022**	0.014**	0.011**
Satisfied with Health Ministry COVID Management	0.432	0.515	0.569	0.664
Would Vote for Current President	0.416	0.325	0.331	0.297
Would Vote for Current Mayor	0.772	0.697	0.581	0.538
Trust in Current President	0.332	0.459	0.534	0.539
Trust in Current Mayor	0.048**	0.097*	0.083*	0.081*
Trust in National Health Ministry	0.492	0.603	0.63	0.763
Trust in National Medical Association	0.95	0.931	0.902	0.94
Trust in Left-Wing Newspaper	0.661	0.697	0.69	0.75
Trust in Right-Wing Newspaper	0.66	0.814	0.793	0.827
Trust in Religious Leader	0.718	0.763	0.738	0.696
Trust in Local Healthcare	0.578	0.459	0.503	0.649
Trust in Armed Forces	0.423	0.439	0.476	0.578
Trust in Civil Society Organizations	0.77	0.8	0.739	0.72
Trust in Government of China	0.331	0.433	0.478	0.502
Trust in Government of U.S. Under Trump	0.031**	0.024**	0.03**	0.032**
Trust in Government of U.S. Under Biden	0.26	0.261	0.316	0.327
Trust in Government of U.K.	0.418	0.394	0.405	0.59
Trust in Government of Russia	0.242	0.26	0.232	0.231
Meeting Indoor With Non-Family Contributes to COVID	0.165	0.221	0.257	0.297
Risk Aversion 1	0.373	0.458	0.419	0.37
Risk Aversion 2	0.09*	0.159	0.179	0.116
Risk Aversion 3	0.459	0.631	0.662	0.625
Risk Aversion 4	0.479	0.6	0.52	0.345
Risk Aversion 5	0.873	0.894	0.897	0.855
Discount Rate 1	0.925	0.941	0.958	0.975
Discount Rate 2	0.842	0.892	0.848	0.848
Discount Rate 3	0.737	0.79	0.799	0.878
Discount Rate 4	0.411	0.497	0.524	0.588
Donation Amount	0.241	0.296	0.3	0.36
Important to Receive Respect and Recognition	0.756	0.784	0.716	0.764
Social Influence	0.103	0.064*	0.063*	0.091*

Table S11: Balance of vaccine information treatments over pre-treatment covariates. Each number is the p value associated with the test of the null hypothesis that no treatment condition differs from the control group in terms of a given pre-treatment covariate. All specifications include country $\times$ block fixed effects and (standardized) pre-treatment wait until vaccination as covariates (omitted to save space), weight observations by the inverse probability of treatment assignment, and are estimated using OLS. Robust standard errors are in parentheses. * denotes $p < 0.1$, ** denotes $p < 0.05$, *** denotes $p < 0.01$ from two-sided t tests.

	**Sample for which balance is tested**:			
Pre-treatment covariate	Received treatment (1)	Answered vaccine willingness scale (2)	Answered wait until vaccination (3)	Answered encourage others to get vaccinated (4)
Education - None	0.799	0.458	0.455	0.467
Education - Primary	0.159	0.174	0.201	0.17
Education - Secondary	0.636	0.664	0.695	0.873
Education - Other Higher	0.828	0.856	0.823	0.961
Education - University	0.306	0.32	0.35	0.369
Gender	0.521	0.437	0.492	0.375
Running Water in Home	0.182	0.201	0.209	0.249
Sewage in Home	0.825	0.851	0.816	0.757
Electricity in Home	0.986	0.981	0.983	0.942
No Running Water, Sewage, or Electricity in Home	0.205	0.173	0.222	0.253
COVID News Consumption - TV	0.734	0.741	0.829	0.892
COVID News Consumption - Radio	0.484	0.486	0.487	0.52
COVID News Consumption - Print	0.946	0.908	0.893	0.89
COVID News Consumption - Word of Mouth	0.474	0.413	0.382	0.5
COVID News Consumption - WhatsApp	0.937	0.938	0.91	0.693
COVID News Consumption - Social Media	0.834	0.807	0.819	0.86
COVID News Consumption - News Websites	0.728	0.692	0.705	0.609
COVID Severity in Country	0.241	0.19	0.216	0.205
Herd Immunity Prior	0.211	0.308	0.387	0.275
General Vaccine Hesitancy - Protect from Disease	0.601	0.657	0.657	0.612
General Vaccine Hesitancy - Good for Community	0.209	0.301	0.263	0.272
General Vaccine Hesitancy - Trust in Government	0.385	0.462	0.399	0.516
General Vaccine Hesitancy - Follow Doctor Instructions	0.59	0.605	0.605	0.64
General Vaccine Hesitancy - Trust in International Medical Experts	0.67	0.638	0.594	0.581
General Vaccine Hesitancy - Refused Vaccine	0.988	0.965	0.978	0.932
COVID Hesitancy Reasons - Side Effects	0.99	0.98	0.98	0.955
COVID Hesitancy Reasons - Vaccine Gives COVID	0.003***	0.002***	0.003***	0.006***
COVID Hesitancy Reasons - Produced Too Quickly	0.153	0.117	0.09*	0.119
COVID Hesitancy Reasons - Not Effective	0.154	0.181	0.21	0.33
COVID Hesitancy Reasons - Not At Risk of Getting COVID	0.575	0.643	0.601	0.586
COVID Hesitancy Reasons - Against Vaccines Generally	0.867	0.858	0.935	0.842
COVID Hesitancy Reasons - Prefer ’Natural’ Immunity	0.895	0.875	0.9	0.868
COVID Hesitancy Reasons - Already Had COVID	0.767	0.846	0.839	0.835
COVID Hesitancy Reasons - Don’t Trust Government	0.248	0.556	0.549	0.568
COVID Hesitancy Reasons - Financial Concerns	0.245	0.322	0.324	0.349
COVID Hesitancy Reasons - Other	0.525	0.563	0.514	0.35
Comorbidities - None	0.033**	0.027**	0.029**	0.035**
Comorbidities - Diabetes	0.633	0.546	0.609	0.618
Comorbidities - Cardiovascular Diseases	0.879	0.717	0.647	0.506
Comorbidities - Obesity	0.239	0.264	0.231	0.324
Comorbidities - Autoimmune Diseases	0.898	0.852	0.859	0.93
Comorbidities - Chronic Obstructive Pulmonary Disease	0.572	0.536	0.537	0.761
Comorbidities - Prefer Not To Share	0.036**	0.059*	0.054*	0.03**
Had COVID	0.567	0.575	0.645	0.682
Know Someone Seriously Ill or Passed Away COVID	0.132	0.119	0.119	0.159
COVID Economic Situation	0.109	0.171	0.204	0.241
Government Vaccine Priority	0.112	0.082*	0.088*	0.087*
Left/Right Political Scale	0.798	0.818	0.793	0.791
Satisfied with President COVID Management	0.291	0.259	0.269	0.338
Satisfied with Mayor COVID Management	0.236	0.231	0.243	0.239
Satisfied with Health Ministry COVID Management	0.875	0.841	0.829	0.836
Would Vote for Current President	0.011**	0.013**	0.009***	0.014**
Would Vote for Current Mayor	0.542	0.573	0.696	0.603
Trust in Current President	0.681	0.706	0.701	0.737
Trust in Current Mayor	0.621	0.709	0.737	0.669
Trust in National Health Ministry	0.885	0.849	0.886	0.831
Trust in National Medical Association	0.07*	0.11	0.171	0.213
Trust in Left-Wing Newspaper	0.546	0.53	0.507	0.777
Trust in Right-Wing Newspaper	0.089*	0.106	0.099*	0.134
Trust in Religious Leader	0.832	0.818	0.8	0.751
Trust in Local Healthcare	0.028**	0.038**	0.058*	0.071*
Trust in Armed Forces	0.208	0.181	0.177	0.363
Trust in Civil Society Organizations	0.069*	0.09*	0.099*	0.141
Trust in Government of China	0.133	0.082*	0.057*	0.191
Trust in Government of U.S. Under Trump	0.579	0.578	0.555	0.742
Trust in Government of U.S. Under Biden	0.026**	0.005***	0.007***	0.018**
Trust in Government of U.K.	0.458	0.437	0.434	0.664
Trust in Government of Russia	0.642	0.884	0.879	0.791
Meeting Indoor With Non-Family Contributes to COVID	0.449	0.433	0.437	0.337
Risk Aversion 1	0.413	0.341	0.285	0.226
Risk Aversion 2	0.676	0.785	0.808	0.784
Risk Aversion 3	0.354	0.535	0.566	0.644
Risk Aversion 4	0.75	0.922	0.92	0.989
Risk Aversion 5	0.148	0.441	0.525	0.516
Discount Rate 1	0.058*	0.04**	0.049**	0.065*
Discount Rate 2	0.011**	0.013**	0.022**	0.022**
Discount Rate 3	0.006***	0.015**	0.022**	0.032**
Discount Rate 4	0.021**	0.065*	0.087*	0.106
Donation Amount	0.545	0.513	0.51	0.62
Important to Receive Respect and Recognition	0.042**	0.06*	0.083*	0.148
Social Influence	0.246	0.195	0.156	0.208

Table S12: Balance of motivational messages over pre-treatment covariates. Each number is the p value associated with the test of the null hypothesis that no treatment condition differs from the control group in terms of a given pre-treatment covariate. All specifications include country $\times$ block fixed effects and (standardized) pre-treatment wait until vaccination as covariates (omitted to save space) and are estimated using OLS. Robust standard errors are in parentheses. * denotes $p < 0.1$, ** denotes $p < 0.05$, *** denotes $p < 0.01$ from two-sided t tests.

	Outcome variable:			
	Vaccine willingness scale	Willing to take a vaccine	Months would wait to get vaccinated (reversed)	Encourage others to get vaccinated
	(1)	(2)	(3)	(4)
Any vaccine information effect 95% confidence interval	[0.051, 0.217]	[0.017, 0.074]	[0.117, 0.686]	[0.003, 0.070]
Outcome range	[1,5]	{0,1}	[0,12]	{0,1}
Control outcome mean	3.24	0.42	5.98	0.56
Control outcome std. dev.	1.18	0.49	4.43	0.50
Number of selected observations	6,986	6,986	6,910	6,706
Share of control observations trimmed	0.017	0.017	0.017	0.024

Table S13: Lee bounds on the effect of any vaccine information on vaccine willingness. All 95% confidence intervals for the treatment effect are based on Lee bound estimates, where observations are weighted by the inverse probability of treatment assignment. Confidence intervals are based on robust standard errors.

tant reasons to be confident that social approval produces positive effects on vaccine willingness. First, as Table S12 shows, attrition does not induce observable differences between the social approval and control groups. This suggests that attrition plausibly occurs somewhat randomly within treatment groups, implying that it is not the most hesitant respondents that differentially attrited from the control group—the case that corresponds to the lower Lee bound. Second, because there are no differences in attrition between motivational message groups, we can estimate the effect of the the social approval treatment relative to the altruistic treatment, which seems to have had limited impact on respondents. The results in Table S15, which compares these two groups, indicates that the social approval treatment produced a significantly larger effect than the altruistic treatment. This adds further weight to the conclusion that social approval messaging could produce substantial positive effects on vaccine uptake.

	Outcome variable:			
	Vaccine willingness scale (1)	Willing to take a vaccine (2)	Months would wait to get vaccinated (reversed) (3)	Encourage others to get vaccinated (4)
**Panel A: Altruism message**				
Altruism effect 95% confidence interval	[-0.146, 0.209]	[-0.047, 0.064]	[-0.596, 0.598]	[-0.042, 0.079]
Outcome range	[1,5]	{0,1}	[0,12]	{0,1}
Control outcome mean	3.25	0.42	6.07	0.56
Control outcome std. dev.	1.18	0.49	4.43	0.50
Number of selected observations	3,471	3,471	3,431	3,321
Share of control observations trimmed	0.050	0.050	0.053	0.056
**Panel B: Economic recovery message**				
Economic recovery effect 95% confidence interval	[-0.107, 0.231]	[-0.037, 0.070]	[-0.606, 0.520]	[-0.027, 0.087]
Outcome range	[1,5]	{0,1}	[0,12]	{0,1}
Control outcome mean	3.26	0.43	6.05	0.56
Control outcome std. dev.	1.18	0.49	4.45	0.50
Number of selected observations	3,466	3,466	3,424	3,313
Share of control observations trimmed	0.047	0.047	0.048	0.051
**Panel C: Social approval message**				
Social approval effect 95% confidence interval	[-0.066, 0.283]	[-0.015, 0.095]	[-0.457, 0.753]	[-0.018, 0.102]
Outcome range	[1,5]	{0,1}	[0,12]	{0,1}
Control outcome mean	3.28	0.44	6.14	0.57
Control outcome std. dev.	1.16	0.50	4.44	0.50
Number of selected observations	3,480	3,480	3,443	3,331
Share of control observations trimmed	0.049	0.049	0.053	0.056

Table S14: Lee bounds on the effect of different types of motivational message on vaccine willingness. All 95% confidence intervals for the treatment effect are based on Lee bound estimates. Confidence intervals are based on robust standard errors.

	Outcome variable:			
	Vaccine willingness scale	Willing to take a vaccine	Months would wait to get vaccinated (reversed)	Encourage others to get vaccinated
	(1)	(2)	(3)	(4)
Social approval	0.077**	0.031**	0.188**	0.024*
	(0.031)	(0.013)	(0.083)	(0.014)
Outcome range	[1,5]	{0,1}	[0,12]	{0,1}
Control outcome mean	3.29	0.45	6.14	0.58
Control outcome std. dev.	1.17	0.50	4.45	0.49
Observations	3,485	3,485	3,452	3,346
R^2	0.446	0.466	0.724	0.348

Table S15: The effect of social approval versus altruistic motivational messages on vaccine willingness. All specifications include country $\times$ block fixed effects and (standardized) pre-treatment wait until vaccination as covariates (omitted to save space) and are estimated using OLS. The baseline category is the altruism message treatment. Robust standard errors are in parentheses. * denotes $p < 0.1$, ** denotes $p < 0.05$, *** denotes $p < 0.01$ from two-sided t tests.
