# Supplementary material for: Messages that increase COVID-19 vaccine acceptance: Evidence from online experiments in six Latin American countries
Source: PLoS One. 2021 Oct 28;16(10):e0259059. doi: 10.1371/journal.pone.0259059 (PMC8553119; doi:10.1371/journal.pone.0259059)
Supplement: S8 Appendix — (PDF) [file pone.0259059.s008.pdf]

S8 Differential effects of vaccine information treatments on reasons given for reducing hesitancy

Among the subset of respondents that received an information treatment, we later asked how the treatment affected their reasons for being hesitant. Since this question was only asked of treated respondents, we examine the effect of the more detailed information treatments relative to the effect of the basic vaccine information. The basic vaccine information group means at the foot of Table S16 show that respondents became less hesitant along a number of dimensions, while the treatment effect estimates indicate that no additional information treatment condition systematically affected the reasons that individuals stated for becoming less hesitant. In line with the lack of differential effect of the information treatments on our vaccine willingness outcomes, the results suggest that basic vaccine information was sufficient to significantly reduce vaccine hesitancy and that further information did not make individuals less hesitant.

	**Outcome variable:**								
	Less worried about side effects (1)	Less worried about getting COVID-19 from vaccine (2)	Less worried about speed of development (3)	Less worried about vaccine ineffectiveness (4)	No getting vaccinated even if low risk (5)	No longer wants immunity from infection (6)	Now getting vaccinated even if already had COVID-19 (7)	Now more trusting of government (8)	Less worried about cost (9)
Vaccine + Biden	−0.000	0.004	0.018	0.002	0.001	0.003	0.012	−0.004	−0.000
	(0.015)	(0.016)	(0.016)	(0.017)	(0.013)	(0.011)	(0.012)	(0.010)	(0.013)
Vaccine + Herd 60%	0.018	−0.022	−0.024	0.017	0.027	0.017	−0.000	0.001	−0.003
	(0.019)	(0.019)	(0.019)	(0.022)	(0.018)	(0.015)	(0.014)	(0.013)	(0.017)
Vaccine + Herd 70%	0.040**	0.022	−0.001	0.036*	−0.005	0.016	0.009	0.004	−0.020
	(0.020)	(0.020)	(0.020)	(0.022)	(0.017)	(0.015)	(0.015)	(0.013)	(0.016)
Vaccine + Herd 80%	0.023	−0.001	−0.010	0.023	0.019	0.014	0.006	0.021	0.026
	(0.019)	(0.020)	(0.019)	(0.022)	(0.017)	(0.015)	(0.015)	(0.014)	(0.018)
Vaccine + Herd 60% + Current	0.034*	−0.004	0.014	0.027	0.028	−0.012	0.003	−0.003	0.005
	(0.020)	(0.020)	(0.020)	(0.022)	(0.018)	(0.014)	(0.015)	(0.012)	(0.017)
Vaccine + Herd 70% + Current	0.035*	0.007	0.001	0.006	0.003	−0.006	−0.030**	−0.006	0.017
	(0.020)	(0.020)	(0.020)	(0.022)	(0.017)	(0.014)	(0.013)	(0.012)	(0.017)
Vaccine + Herd 80% + Current	0.005	−0.019	0.006	−0.037*	0.016	−0.003	0.004	−0.005	−0.036**
	(0.019)	(0.020)	(0.020)	(0.020)	(0.017)	(0.014)	(0.015)	(0.012)	(0.015)
Outcome range	{0,1}	{0,1}	{0,1}	{0,1}	{0,1}	{0,1}	{0,1}	{0,1}	{0,1}
Control outcome mean	0.17	0.19	0.19	0.26	0.12	0.08	0.09	0.06	0.12
Control outcome std. dev.	0.38	0.39	0.39	0.44	0.32	0.27	0.28	0.25	0.32
Observations	5,619	5,619	5,619	5,619	5,619	5,619	5,619	5,619	5,619
R^2	0.103	0.081	0.069	0.151	0.057	0.047	0.095	0.070	0.062

Table S16: Effect of different types of vaccine information on reasons for becoming less hesitant, among treated respondents. All specifications include country $\times$ block fixed effects and (standardized) pre-treatment wait until vaccination as covariates (omitted to save space), weight observations by the inverse probability of treatment assignment, and are estimated using OLS. Because control respondents did not answer this question, the baseline category is the Vaccine only information treatment. Robust standard errors are in parentheses. * denotes $p < 0.1$, ** denotes $p < 0.05$, *** denotes $p < 0.01$ from two-sided t tests.
