# Supplementary material for: Messages that increase COVID-19 vaccine acceptance: Evidence from online experiments in six Latin American countries
Source: PLoS One. 2021 Oct 28;16(10):e0259059. doi: 10.1371/journal.pone.0259059 (PMC8553119; doi:10.1371/journal.pone.0259059)
Supplement: S9 Appendix — (PDF) [file pone.0259059.s009.pdf]

S9 Heterogeneity in the effect of basic vaccine information

To understand which types of individuals may be most responsive to exposure to basic vaccine information, we examine heterogeneity in treatment effects across demographic subgroups about which policymakers can conceivably obtain data at scale—and could thus microtarget campaign messaging towards. Specifically, we consider a respondent's sex, age category, highest level of completed education, socioeconomic class, and intention to vote for the President. Using the specifications described in S3 Appendix, Table S17 shows that the treatments produced similar effects on different types of hesitant respondent. The only systematic difference is that basic vaccine information is slightly more effective at persuading women to vaccinate than men.

	Outcome variable:			
	Vaccine willingness scale (1)	Willing to take a vaccine (2)	Months would wait to get vaccinated (reversed) (3)	Encourage others to get vaccinated (4)
Any vaccine information	0.343	0.136	0.109	−0.002
	(0.253)	(0.109)	(0.645)	(0.120)
Any vaccine information × Woman	0.028	0.039*	0.239**	0.014
	(0.050)	(0.021)	(0.120)	(0.025)
Any vaccine information × Aged 25-34	0.088	−0.010	−0.136	−0.021
	(0.074)	(0.032)	(0.169)	(0.038)
Any vaccine information × Aged 35-44	0.078	−0.007	−0.062	−0.056
	(0.076)	(0.033)	(0.184)	(0.041)
Any vaccine information × Aged 45-54	0.039	−0.032	−0.142	−0.022
	(0.083)	(0.034)	(0.189)	(0.040)
Any vaccine information × Aged 55-64	−0.057	−0.029	−0.590**	−0.029
	(0.094)	(0.039)	(0.254)	(0.044)
Any vaccine information × Aged 65+	0.097	−0.002	−0.217	0.015
	(0.092)	(0.039)	(0.201)	(0.044)
Any vaccine information × Middle SES	−0.120	−0.035	0.133	−0.010
	(0.097)	(0.037)	(0.213)	(0.044)
Any vaccine information × High SES	−0.083	−0.034	0.255	−0.016
	(0.091)	(0.034)	(0.206)	(0.042)
Any vaccine information × Would vote for President	0.081	0.008	0.010	0.004
	(0.065)	(0.025)	(0.141)	(0.028)
Any vaccine information × Primary education	0.053	−0.061	0.192	0.133
	(0.242)	(0.105)	(0.597)	(0.112)
Any vaccine information × Secondary education	−0.230	−0.111	−0.186	0.041
	(0.222)	(0.098)	(0.580)	(0.104)
Any vaccine information × University education	−0.266	−0.121	−0.169	0.060
	(0.225)	(0.099)	(0.586)	(0.105)
Any vaccine information × Other higher education	−0.197	−0.107	0.131	0.043
	(0.229)	(0.100)	(0.591)	(0.106)
Outcome range	[1,5]	{0,1}	[0,12]	{0,1}
Control outcome mean	3.17	0.40	5.78	0.54
Control outcome std. dev	1.18	0.49	4.38	0.50
Observations	6,947	6,947	6,872	6,655
R^2	0.487	0.494	0.767	0.361

Table S17: Effect of any vaccine information on vaccine willingness, by pre-treatment covariate. All specifications include country × block fixed effects and (standardized) pre-treatment wait until vaccination as covariates (omitted to save space), weight observations by the inverse probability of treatment assignment, and are estimated using OLS. Lower-order interaction terms are omitted to save space; the omitted categories are aged 18-24, would not vote for the President, and university education. Robust standard errors are in parentheses. * denotes $p < 0.1$, ** denotes $p < 0.05$, *** denotes $p < 0.01$ from two-sided t tests.
