# Supplementary material for: Messages that increase COVID-19 vaccine acceptance: Evidence from online experiments in six Latin American countries
Source: PLoS One. 2021 Oct 28;16(10):e0259059. doi: 10.1371/journal.pone.0259059 (PMC8553119; doi:10.1371/journal.pone.0259059)
Supplement: S10 Appendix — (PDF) [file pone.0259059.s010.pdf]

S10 Heterogeneity in the effect of herd immunity information

We next examine the effect of herd immunity treatments that induced respondents to update their beliefs to different degrees and in different directions, relative to their prior beliefs. Using the specification described in S3 Appendix, column (1) first seeks to validate whether the herd immunity information altered respondents' posterior beliefs about the level of vaccination required to achieve herd immunity. Indeed, respondents whose prior beliefs were below (above) the expert opinion that they were exposed to updated their posterior beliefs upwards (downwards). Columns (2)-(5) then examine the effect of such updating on vaccine willingness, finding little evidence to suggest that respondents that updated their posterior beliefs in different ways adopted different stances toward vaccination. This finding is robust to considering herd immunity treatments that induced respondents to update positively or negatively (panel A) or more subtle forms degrees of updating relative to a control group that received information within 5 percentage points either side of their prior belief (panel B). The results ultimately suggest that learning about herd immunity rates on their own did not play a key role in explaining vaccine willingness.

	Outcome variable:				
	Posterior belief about rate required for herd immunity	Vaccine willingness scale	Willing to take a vaccine	Months would wait to get vaccinated (reversed)	Encourage others to get vaccinated
	(1)	(2)	(3)	(4)	(5)
**Panel A: Prior beliefs above/below reported expert herd rate**					
Prior belief below reported herd rate	3.624***	0.060	−0.012	−0.007	−0.028
	(1.151)	(0.074)	(0.032)	(0.213)	(0.039)
Outcome range	[0-100]	[1-5]	{0,1}	[0,12]	{0,1}
Control outcome mean	84.02	3.57	0.55	7.22	0.69
Control outcome std. dev.	14.89	1.08	0.50	4.06	0.46
Observations	2,801	2,955	2,955	2,919	2,821
R^2	0.637	0.496	0.476	0.729	0.415
**Panel B: Prior beliefs relative to reported expert herd rate**					
Prior belief 5-15pp below reported herd rate	4.282**	0.020	−0.051	−0.220	0.012
	(1.881)	(0.115)	(0.046)	(0.295)	(0.059)
Prior belief 15pp below reported herd rate	6.933***	0.036	0.012	−0.078	−0.028
	(2.393)	(0.137)	(0.055)	(0.387)	(0.070)
Prior belief 5-15pp above reported herd rate	−1.192	−0.063	−0.005	−0.256	0.011
	(1.044)	(0.069)	(0.031)	(0.184)	(0.037)
Prior belief 15pp above reported herd rate	−3.871***	−0.049	0.045	−0.038	0.071*
	(1.289)	(0.082)	(0.039)	(0.230)	(0.043)
Outcome range	[0,100]	[1,5]	{0,1}	[0,12]	{0,1}
Control outcome mean	74.46	3.36	0.44	6.29	0.56
Control outcome std. dev.	12.66	1.10	0.50	4.28	0.50
Observations	2,801	2,955	2,955	2,919	2,821
R^2	0.638	0.496	0.477	0.729	0.416

Table S18: Effect of different types of different expert opinion herd immunity opinion on vaccine willingness, by how the information relates to individual prior beliefs. All specifications include country × block fixed effects, prior belief level fixed effects, and (standardized) pre-treatment wait until vaccination as covariates (omitted to save space) and are estimated using OLS. The sample is restricted to respondents that received a treatment that reported an expert herd immunity rate. Robust standard errors are in parentheses. * denotes $p < 0.1$, ** denotes $p < 0.05$, *** denotes $p < 0.01$ from two-sided t tests.
