# Supplementary material for: Messages that increase COVID-19 vaccine acceptance: Evidence from online experiments in six Latin American countries
Source: PLoS One. 2021 Oct 28;16(10):e0259059. doi: 10.1371/journal.pone.0259059 (PMC8553119; doi:10.1371/journal.pone.0259059)
Supplement: S11 Appendix — (PDF) [file pone.0259059.s011.pdf]

S11 Heterogeneity in the effect of current willingness information

While providing information about the current willingness of the population to get vaccinated does not affect vaccine willingness on average, this null finding may mask variation in responses that depends on the direction in which the information encouraged respondents to update their posterior beliefs about communal uptake rates. Indeed, the free riding logic suggests that individuals that come to believe that more (less) people will get vaccinated than they previously expected, will become less (more) willing to vaccinate themselves. In contrast, if individuals regard the intentions of others as informative about their own costs and benefits or seek to coordinate their behavior with that of others, then we should expect to observe the reverse relationship. Using the specification described in S3 Appendix, Table S19 detects no evidence to support either logic: respondents that were informed of a current willing that exceed their prior belief became no more or less willing to get vaccinated. As the main paper notes, this suggests that simple forms of free riding, social learning, or coordination are unlikely to be important drivers vaccine willingness.

	Outcome variable:				
	Posterior belief about rate municipal willingness	Vaccine willingness scale	Willing to take a vaccine	Months would wait to get vaccinated (reversed)	Encourage others to get vaccinated
	(1)	(2)	(3)	(4)	(5)
**Panel A: Prior beliefs above/below current willingness**					
Current	-1.491^{**}	0.043	0.024	0.253^{**}	0.026
	(0.623)	(0.042)	(0.017)	(0.108)	(0.017)
Prior below current willingness	-25.857^{***}	-0.180^{***}	-0.067^{***}	-0.291^{***}	-0.123^{***}
	(0.551)	(0.027)	(0.011)	(0.071)	(0.013)
Current $\times$ Prior below current willingness	3.296^{***}	0.021	-0.005	-0.091	-0.003
	(1.067)	(0.054)	(0.023)	(0.145)	(0.024)
Outcome range	[0,100]	[1,5]	{0,1}	[0,12]	{0,1}
Control outcome mean	61.81	3.17	0.40	5.78	0.54
Control outcome std. dev.	24.37	1.18	0.49	4.38	0.50
Observations	6,747	6,951	6,951	6,876	6,659
R^2	0.402	0.438	0.444	0.719	0.354
**Panel B: Prior beliefs relative to current willingness**					
Current	-0.184	0.029	0.047	0.078	0.065^{**}
	(1.002)	(0.064)	(0.030)	(0.150)	(0.031)
Prior 5-15pp below current willingness	5.917^{***}	0.079^{*}	0.041^{**}	0.190^{*}	0.044^{**}
	(0.748)	(0.044)	(0.019)	(0.106)	(0.021)
Prior 15pp below current willingness	14.594^{***}	0.059	0.040^{**}	0.100	0.075^{***}
	(0.788)	(0.047)	(0.019)	(0.115)	(0.021)
Prior 5-15pp above current willingness	-8.501^{***}	-0.060	-0.009	-0.050	-0.041^{*}
	(0.826)	(0.043)	(0.019)	(0.114)	(0.022)
Prior 15pp above current willingness	-26.520^{***}	-0.201^{***}	-0.060^{***}	-0.304^{***}	-0.110^{***}
	(0.782)	(0.038)	(0.017)	(0.099)	(0.020)
Current $\times$ Prior 5-15pp below current willingness	-0.687	-0.063	-0.046	0.263	-0.056
	(1.347)	(0.088)	(0.040)	(0.216)	(0.041)
Current $\times$ Prior 15pp below current willingness	-1.124	0.112	0.002	0.140	-0.028
	(1.383)	(0.099)	(0.042)	(0.245)	(0.041)
Current $\times$ Prior 5-15pp above current willingness	2.681^{*}	0.113	-0.025	0.148	-0.039
	(1.590)	(0.087)	(0.042)	(0.242)	(0.046)
Current $\times$ Prior 15pp above current willingness	2.622^{*}	0.013	-0.036	0.083	-0.050
	(1.534)	(0.077)	(0.035)	(0.198)	(0.038)
Outcome range	[0,100]	[1,5]	{0,1}	[0,12]	{0,1}
Control outcome mean	61.810	3.170	0.400	5.780	0.540
Control outcome std. dev.	24.370	1.180	0.490	4.380	0.500
Observations	6,747	6,951	6,951	6,876	6,659
R^2	0.510	0.442	0.447	0.720	0.360

Table S19: Effect of vaccine information on vaccine willingness, by how current willingness relates to individual prior beliefs. All specifications include country $\times$ block fixed effects and (standardized) pre-treatment wait until vaccination as covariates (omitted to save space), weight observations by the inverse probability of treatment assignment, and are estimated using OLS. All treatments and associated interactions are included in panel B, but omitted to save space. Robust standard errors are in parentheses. * denotes $p < 0.1$, ** denotes $p < 0.05$, *** denotes $p < 0.01$ from two-sided t tests.
