# Supplementary material for: Messages that increase COVID-19 vaccine acceptance: Evidence from online experiments in six Latin American countries
Source: PLoS One. 2021 Oct 28;16(10):e0259059. doi: 10.1371/journal.pone.0259059 (PMC8553119; doi:10.1371/journal.pone.0259059)
Supplement: S12 Appendix — (PDF) [file pone.0259059.s012.pdf]

S12 Pre-treatment vaccine hesitancy and prior beliefs

As shown in the main paper, beliefs about the vaccination rates required to achieve herd immunity and the current level of willingness in the population appear to coordinate individuals in a more subtle way: respondents became more willing to get vaccinated when they learned that the population was on track to achieve herd immunity. While the results in the main paper demonstrate this experimentally, we conduct a further analysis based on respondents' prior beliefs to assess this logic correlationally before treatments were delivered. To do so, we examine the interaction between the two prior beliefs using the following OLS regression within our full sample (not just among hesitant respondents):

$$Y_{ic} = \beta_0 + \beta_1 Herd\,prior_{ic} + \beta_2 Willing\,prior_{ic} + \beta_3 (Herd\,prior_{ic} \times Willing\,prior_{ic}) + \varepsilon_{ic}. \quad (8)$$

The results, which are reported in Table S20 for the three outcomes measured before treatment, find a statistically significant positive interaction effect in each case. As with the experimental evidence, this suggests that individuals who believed—before treatment—that a given level of mass vaccination is required to achieve herd immunity were more willing to get vaccinated if they believe that many others are also likely to get vaccinated.

	Outcome variable:		
	Vaccine willingness scale (1)	Willing to take a vaccine (2)	Months would wait to get vaccinated (reversed) (3)
Constant	1.920537^{***}	-0.006324	0.379705^{*}
	(0.067262)	(0.016529)	(0.229238)
Pre-treatment uptake rate	0.003240^{**}	0.000581	0.012471^{**}
	(0.001468)	(0.000414)	(0.004998)
Pre-treatment herd immunity	0.010004^{***}	0.001264^{***}	0.037189^{***}
	(0.000938)	(0.000289)	(0.003279)
Pre-treatment uptake $\times$ herd immunity	0.000032^{*}	0.000042^{***}	0.000288^{***}
	(0.000018)	(0.000006)	(0.000061)
Outcome range	[1,5]	{0,1}	[0,12]
Observations	7,521	7,521	7,521
R^2	0.105	0.099	0.177

Table S20: Correlation between prior beliefs and prior vaccine willingness. All specifications are estimated using OLS. Robust standard errors are in parentheses. * denotes $p < 0.1$, ** denotes $p < 0.05$, *** denotes $p < 0.01$ from two-sided t tests.
