# Supplementary material for: Messages that increase COVID-19 vaccine acceptance: Evidence from online experiments in six Latin American countries
Source: PLoS One. 2021 Oct 28;16(10):e0259059. doi: 10.1371/journal.pone.0259059 (PMC8553119; doi:10.1371/journal.pone.0259059)
Supplement: S13 Appendix — (PDF) [file pone.0259059.s013.pdf]

S13 Heterogeneity in the effect of motivational messages

To understand which types of individuals may be most responsive to different types of motivational message, we again examine heterogeneity in treatment effects across demographic subgroups about which policymakers could conceivably obtain data at scale—and could thus microtarget campaign messaging towards. Specifically, we consider a respondent's sex, age category, highest level of completed education, socioeconomic class, and intention to vote for the President. Estimating the specifications described in S3 Appendix, Table S21 shows that the treatments produced similar effects on different types of hesitant respondent. While there is some evidence to suggest that older respondents were less responsive to the social approval message, the effects are largely similar across types of respondent.

	Outcome variable:			
	Vaccine willingness scale	Willing to take a vaccine	Months would wait to get vaccinated (reversed)	Encourage others to get vaccinated
	(1)	(2)	(3)	(4)
Altruism	−0.307	0.008	−0.138	−0.136
	(0.264)	(0.112)	(0.797)	(0.121)
Economic recovery	−0.179	−0.073	0.703	−0.343***
	(0.259)	(0.113)	(0.639)	(0.128)
Social Approval	0.067	0.219**	1.645**	−0.008
	(0.256)	(0.108)	(0.834)	(0.131)
Altruism × Woman	0.003	−0.010	0.054	−0.012
	(0.062)	(0.026)	(0.166)	(0.029)
Economic recovery × Woman	0.075	0.005	−0.035	−0.010
	(0.062)	(0.026)	(0.165)	(0.029)
Social status × Woman	0.057	−0.014	0.047	0.004
	(0.062)	(0.026)	(0.174)	(0.030)
Altruism × Aged 25-34	0.015	−0.037	0.041	−0.024
	(0.095)	(0.041)	(0.244)	(0.046)
Economic recovery × Aged 25-34	0.061	−0.038	−0.232	0.086*
	(0.095)	(0.041)	(0.237)	(0.046)
Social status × Aged 25-34	0.039	−0.038	0.525**	0.012
	(0.093)	(0.041)	(0.249)	(0.047)
Altruism × Aged 35-44	−0.030	−0.028	−0.009	0.005
	(0.093)	(0.041)	(0.273)	(0.048)
Economic recovery × Aged 35-44	−0.064	−0.056	−0.265	0.046
	(0.095)	(0.041)	(0.265)	(0.048)
Social status × Aged 35-44	−0.095	−0.052	0.358	−0.024
	(0.096)	(0.042)	(0.271)	(0.048)
Altruism × Aged 45-54	0.142	0.047	0.047	0.041
	(0.102)	(0.043)	(0.284)	(0.049)
Economic recovery × Aged 45-54	−0.053	−0.039	−0.346	0.028
	(0.103)	(0.042)	(0.282)	(0.048)
Social status × Aged 45-54	−0.038	−0.035	−0.020	0.003
	(0.103)	(0.042)	(0.295)	(0.049)
Altruism × Aged 55-64	0.127	0.016	0.180	−0.027
	(0.114)	(0.047)	(0.294)	(0.051)
Economic recovery × Aged 55-64	0.092	0.019	0.087	0.025
	(0.124)	(0.052)	(0.303)	(0.053)
Social status × Aged 55-64	0.082	0.019	0.374	−0.054
	(0.116)	(0.049)	(0.309)	(0.053)
Altruism × Aged 65+	−0.153	−0.053	−0.046	−0.024
	(0.118)	(0.050)	(0.294)	(0.053)
Economic recovery × Aged 65+	−0.079	−0.061	−0.575*	0.030
	(0.112)	(0.049)	(0.310)	(0.054)
Social status × Aged 65+	−0.106	−0.117**	−0.232	−0.028
	(0.114)	(0.048)	(0.303)	(0.054)
Altruism × Middle SES	0.105	0.010	0.413	0.012
	(0.124)	(0.049)	(0.293)	(0.052)
Economic recovery × Middle SES	0.101	0.033	0.255	0.035
	(0.128)	(0.049)	(0.286)	(0.052)
Social status × Middle SES	0.108	0.003	−0.076	0.087*
	(0.123)	(0.047)	(0.320)	(0.052)
Altruism × High SES	0.037	−0.016	0.304	0.033
	(0.119)	(0.046)	(0.271)	(0.049)
Economic recovery × High SES	0.107	0.035	0.191	0.038
	(0.122)	(0.046)	(0.264)	(0.049)
Social status × High SES	0.118	0.014	0.080	0.104**
	(0.119)	(0.045)	(0.304)	(0.049)
Altruism × Would vote for President	−0.024	−0.029	−0.008	−0.081**
	(0.082)	(0.033)	(0.214)	(0.035)
Economic recovery × Would vote for President	0.102	0.030	0.445**	0.026
	(0.085)	(0.034)	(0.222)	(0.036)
Social status × Would vote for President	0.014	−0.022	−0.225	−0.047
	(0.086)	(0.034)	(0.220)	(0.036)
Altruism × Primary education	0.078	0.011	−0.493	0.138
	(0.235)	(0.104)	(0.727)	(0.113)
Economic recovery × Primary education	−0.024	0.096	−0.790	0.352***
	(0.224)	(0.101)	(0.583)	(0.115)
Social status × Primary education	0.044	−0.081	−1.412*	0.023
	(0.224)	(0.099)	(0.755)	(0.121)
Altruism × Secondary education	0.273	0.041	−0.200	0.168*
	(0.210)	(0.093)	(0.684)	(0.099)
Economic recovery × Secondary education	−0.023	0.066	−0.793	0.328***
	(0.199)	(0.091)	(0.485)	(0.104)
Social status × Secondary education	−0.170	−0.144	−1.716**	−0.042
	(0.198)	(0.088)	(0.706)	(0.110)
Altruism × University education	0.295	0.059	−0.113	0.193*
	(0.212)	(0.095)	(0.685)	(0.100)
Economic recovery × University education	0.041	0.107	−0.702	0.273***
	(0.203)	(0.092)	(0.487)	(0.105)
Social status × University education	−0.086	−0.104	−1.695**	−0.011
	(0.202)	(0.089)	(0.712)	(0.111)
Altruism × Other higher education	0.219	0.030	−0.367	0.140
	(0.215)	(0.096)	(0.697)	(0.102)
Economic recovery × Other higher education	0.020	0.066	−0.670	0.330***
	(0.207)	(0.094)	(0.511)	(0.107)
Social status × Other higher education	−0.208	−0.125	−1.658**	−0.035
	(0.207)	(0.092)	(0.727)	(0.113)
Outcome range	[1,5]	{0,1}	[0,12]	{0,1}
Control outcome mean	3.24	0.42	6.07	0.55
Control outcome std. dev	1.17	0.49	4.41	0.50
Observations	6,947	6,947	6,872	6,655
R^2	0.447	0.460	0.730	0.346

Table S21: Effect of any motivational messages on vaccine willingness, by pre-treatment covariate. All specifications include country × block fixed effects and (standardized) pre-treatment wait until vaccination as covariates (omitted to save space) and are estimated using OLS. Lower-order interaction terms are omitted to save space. Robust standard errors are in parentheses. * denotes $p < 0.1$, ** denotes $p < 0.05$, *** denotes $p < 0.01$ from two-sided t tests.
