# Supplementary material for: Messages that increase COVID-19 vaccine acceptance: Evidence from online experiments in six Latin American countries
Source: PLoS One. 2021 Oct 28;16(10):e0259059. doi: 10.1371/journal.pone.0259059 (PMC8553119; doi:10.1371/journal.pone.0259059)
Supplement: S14 Appendix — (PDF) [file pone.0259059.s014.pdf]

S14 Interaction between informational and motivational messages

While both basic vaccine information and social approval messages proved effective at increasing vaccine willingness among hesitant respondents, it may be important from a policy perspective to understand whether these messages serve as substitutes or complements. To do so, we examine the interaction between the two treatment conditions, which were assigned independently. The results in Table S22 find no systematic evidence of a positive or negative interaction between any of the motivational messages and receiving basic vaccine information. This suggests that the two types of messaging campaigns may be largely additive.

	Outcome variable:			
	Vaccine willingness scale	Willing to take a vaccine	Months would wait to get vaccinated (reversed)	Encourage others to get vaccinated
	(1)	(2)	(3)	(4)
Altruism	0.023	0.000	0.149	0.030
	(0.057)	(0.023)	(0.123)	(0.030)
Economic recovery	0.019	0.006	0.108	0.062**
	(0.055)	(0.023)	(0.119)	(0.029)
Social status	0.142**	0.056**	0.394***	0.075**
	(0.057)	(0.025)	(0.150)	(0.031)
Any vaccine information	0.143***	0.038**	0.511***	0.061**
	(0.045)	(0.019)	(0.115)	(0.025)
Altruism × Any vaccine information	0.001	0.019	−0.091	−0.016
	(0.068)	(0.028)	(0.157)	(0.035)
Economic recovery × Any vaccine information	0.045	0.021	−0.147	−0.040
	(0.066)	(0.028)	(0.154)	(0.034)
Social status × Any vaccine information	−0.048	−0.013	−0.171	−0.040
	(0.068)	(0.029)	(0.181)	(0.036)
Outcome range	[1,5]	{0,1}	[0,12]	{0,1}
Control outcome mean	3.16	0.40	5.71	0.51
Control outcome std. dev.	1.15	0.49	4.28	0.50
Observations	6,951	6,951	6,876	6,659
R^2	0.485	0.493	0.767	0.358

Table S22: Effect of any vaccine information on vaccine willingness, by motivational message. All specifications include country × block fixed effects and (standardized) pre-treatment wait until vaccination as covariates (omitted to save space), weight observations by the inverse probability of treatment assignment, and are estimated using OLS. Robust standard errors are in parentheses. * denotes $p < 0.1$, ** denotes $p < 0.05$, *** denotes $p < 0.01$ from two-sided t tests.
