# Supplementary material for: Messages that increase COVID-19 vaccine acceptance: Evidence from online experiments in six Latin American countries
Source: PLoS One. 2021 Oct 28;16(10):e0259059. doi: 10.1371/journal.pone.0259059 (PMC8553119; doi:10.1371/journal.pone.0259059)
Supplement: S15 Appendix — (PDF) [file pone.0259059.s015.pdf]

S15 Effects on encouraging others to vaccinate measured as a scale

In the paper we examine willingness to encourage others to get vaccinated using a binary variable that focuses on those that are likely or very likely do so. Tables S23-S25 show that similar results hold for the underlying four-point scale. While the ordinal measure is more fine-grained, it also assumes that each unit increase in the outcome captures a similar change.

	Outcome variable: Encourage others to get vaccinated scale
	(1)
**Panel A: All countries pooled**	
Any vaccine information	0.094***
	(0.023)
Outcome range	{1,2,3,4}
Control outcome mean	2.56
Control outcome std. dev	1.01
Observations	6,659
R^2	0.417
**Panel B: Argentina**	
Any vaccine information	0.073
	(0.056)
Outcome range	{1,2,3,4}
Control outcome mean	2.44
Control outcome std. dev	0.99
Observations	1,109
R^2	0.389
**Panel C: Brazil**	
Any vaccine information	0.079
	(0.058)
Outcome range	{1,2,3,4}
Control outcome mean	2.45
Control outcome std. dev	1.12
Observations	1,134
R^2	0.483
**Panel D: Chile**	
Any vaccine information	0.155**
	(0.060)
Outcome range	{1,2,3,4}
Control outcome mean	2.37
Control outcome std. dev	1.06
Observations	1,080
R^2	0.418
**Panel E: Colombia**	
Any vaccine information	0.129**
	(0.052)
Outcome range	{1,2,3,4}
Control outcome mean	2.64
Control outcome std. dev	0.97
Observations	1,085
R^2	0.447
**Panel F: México**	
Any vaccine information	0.105*
	(0.055)
Outcome range	{1,2,3,4}
Control outcome mean	2.80
Control outcome std. dev	0.93
Observations	1,075
R^2	0.367
**Panel G: Perú**	
Any vaccine information	0.026
	(0.053)
Outcome range	{1,2,3,4}
Control outcome mean	2.63
Control outcome std. dev	0.91
Observations	1,176
R^2	0.342

Table S23: Effect of any vaccine information on willingness to encourage others to get vaccinated scale. All specifications include country $\times$ block fixed effects and (standardized) pre-treatment wait until vaccination as covariates (omitted to save space), weight observations by the inverse probability of treatment assignment, and are estimated using OLS. Robust standard errors are in parentheses. * denotes $p < 0.1$, ** denotes $p < 0.05$, *** denotes $p < 0.01$ from two-sided t tests.

	**Outcome variable:** Encourage others to get vaccinated scale (1)
Vaccine	0.083***
	(0.030)
Vaccine + Herd 60%	0.081*
	(0.042)
Vaccine + Herd 70%	0.100**
	(0.043)
Vaccine + Herd 80%	0.080*
	(0.043)
Vaccine + Herd 60% + Current	0.160***
	(0.041)
Vaccine + Herd 70% + Current	0.116***
	(0.042)
Vaccine + Herd 80% + Current	0.062
	(0.042)
Vaccine + Biden	0.090***
	(0.035)
Outcome range	{1,2,3,4}
Control outcome mean	2.56
Control outcome std. dev	1.01
Observations	6,659
R^2	0.397

Table S24: Effect of different types of vaccine information treatment on willingness to encourage others to get vaccinated scale. All specifications include country × block fixed effects and (standardized) pre-treatment wait until vaccination as covariates (omitted to save space), weight observations by the inverse probability of treatment assignment, and are estimated using OLS. Robust standard errors are in parentheses. * denotes $p < 0.1$, ** denotes $p < 0.05$, *** denotes $p < 0.01$ from two-sided t tests.

	**Outcome variable:** Encourage others to get vaccinated scale
	(1)
**Panel A: All countries pooled**	
Altruism	0.029
	(0.028)
Economic recovery	0.043
	(0.028)
Social approval	0.080***
	(0.028)
Outcome range	{1,2,3,4}
Control outcome mean	2.60
Control outcome std. dev	1.01
Observations	6,659
R^2	0.395
**Panel B: Argentina**	
Altruism	0.012
	(0.069)
Economic recovery	0.006
	(0.067)
Social approval	0.031
	(0.071)
Outcome range	{1,2,3,4}
Control outcome mean	2.52
Control outcome std. dev	0.98
Observations	1,109
R^2	0.374
**Panel C: Brazil**	
Altruism	0.022
	(0.066)
Economic recovery	0.001
	(0.068)
Social approval	0.095
	(0.064)
Outcome range	{1,2,3,4}
Control outcome mean	2.48
Control outcome std. dev	1.08
Observations	1,134
R^2	0.475
**Panel D: Chile**	
Altruism	0.078
	(0.074)
Economic recovery	0.103
	(0.071)
Social approval	0.094
	(0.074)
Outcome range	{1,2,3,4}
Control outcome mean	2.48
Control outcome std. dev	1.06
Observations	1,080
R^2	0.390
**Panel E: Colombia**	
Altruism	0.132**
	(0.064)
Economic recovery	0.062
	(0.064)
Social approval	0.107*
	(0.063)
Outcome range	{1,2,3,4}
Control outcome mean	2.65
Control outcome std. dev	0.94
Observations	1,085
R^2	0.411
**Panel F: México**	
Altruism	0.030
	(0.069)
Economic recovery	0.064
	(0.070)
Social approval	0.075
	(0.070)
Outcome range	{1,2,3,4}
Control outcome mean	2.84
Control outcome std. dev	0.96
Observations	1,075
R^2	0.344
**Panel G: Perú**	
Altruism	−0.091
	(0.069)
Economic recovery	0.029
	(0.068)
Social approval	0.086
	(0.070)
Outcome range	{1,2,3,4}
Control outcome mean	2.63
Control outcome std. dev	0.97
Observations	1,176
R^2	0.328

Table S25: Effect of different types of motivational message on willingness to encourage others to get vaccinated scale. All specifications include country $\times$ block fixed effects and (standardized) pre-treatment wait until vaccination as covariates (omitted to save space) and are estimated using OLS. Robust standard errors are in parentheses. * denotes $p < 0.1$, ** denotes $p < 0.05$, *** denotes $p < 0.01$ from two-sided t tests.
