# Supplementary material for: Messages that increase COVID-19 vaccine acceptance: Evidence from online experiments in six Latin American countries
Source: PLoS One. 2021 Oct 28;16(10):e0259059. doi: 10.1371/journal.pone.0259059 (PMC8553119; doi:10.1371/journal.pone.0259059)
Supplement: S16 Appendix — (PDF) [file pone.0259059.s016.pdf]

S16 Demand for further information

The main paper focused on vaccine willingness in terms of intentions to act get vaccinated and encourage others to get vaccinated. To examine a less direct behavioral outcome, we also report the effects of the treatments on interest in receiving additional information COVID-19 vaccines from the Pan American Health Organization. Tables S26-S28 report the effects of the information and motivational treatments on seeking such information. In each case, we find little evidence to suggest that the treatments moved interest in receiving further information. It should be noted that this opportunity came after a 25 minute survey that already provided treated respondents with considerable vaccine information already. The null effects could then be explained by treated respondents already feeling sufficiently informed about COVID-19 vaccines that they did not need to expend additional effort to acquire further information.

	Outcome variable:	
	Requested more information	Visited PAHO website
	(1)	(2)
**Panel A: All countries pooled**		
Any vaccine information	-0.020	0.001
	(0.015)	(0.012)
Outcome range	{0,1}	{0,1}
Control outcome mean	0.57	0.22
Control outcome std. dev.	0.50	0.42
Observations	6,082	6,082
R^2	0.107	0.097
**Panel B: Argentina**		
Any vaccine information	0.008	0.041
	(0.037)	(0.028)
Outcome range	{0,1}	{0,1}
Control outcome mean	0.48	0.19
Control outcome std. dev.	0.50	0.40
Observations	1,019	1,019
R^2	0.088	0.161
**Panel C: Brazil**		
Any vaccine information	-0.006	0.012
	(0.038)	(0.029)
Outcome range	{0,1}	{0,1}
Control outcome mean	0.49	0.19
Control outcome std. dev.	0.50	0.39
Observations	1,007	1,007
R^2	0.054	0.060
**Panel D: Chile**		
Any vaccine information	-0.020	0.046
	(0.036)	(0.030)
Outcome range	{0,1}	{0,1}
Control outcome mean	0.52	0.18
Control outcome std. dev.	0.50	0.38
Observations	1,006	1,006
R^2	0.110	0.088
**Panel E: Colombia**		
Any vaccine information	-0.033	-0.029
	(0.035)	(0.032)
Outcome range	{0,1}	{0,1}
Control outcome mean	0.62	0.25
Control outcome std. dev.	0.48	0.44
Observations	1,011	1,011
R^2	0.101	0.080
**Panel F: México**		
Any vaccine information	-0.039	-0.050
	(0.036)	(0.031)
Outcome range	{0,1}	{0,1}
Control outcome mean	0.62	0.26
Control outcome std. dev.	0.49	0.44
Observations	1,005	1,005
R^2	0.085	0.100
**Panel G: Perú**		
Any vaccine information	-0.031	-0.016
	(0.033)	(0.032)
Outcome range	{0,1}	{0,1}
Control outcome mean	0.68	0.27
Control outcome std. dev.	0.46	0.45
Observations	1,034	1,034
R^2	0.114	0.093

Table S26: Effect of any vaccine information on demand for further vaccine information. All specifications include country $\times$ block fixed effects and (standardized) pre-treatment wait until vaccination as covariates (omitted to save space), weight observations by the inverse probability of treatment assignment, and are estimated using OLS. Robust standard errors are in parentheses. * denotes $p < 0.1$, ** denotes $p < 0.05$, *** denotes $p < 0.01$ from two-sided t tests.

	**Outcome variable:**	
	Requested more information	Visited PAHO website
	(1)	(2)
Vaccine	-0.009	0.012
	(0.019)	(0.017)
Vaccine + Biden	-0.027	-0.014
	(0.022)	(0.018)
Vaccine + Herd 60%	-0.021	-0.001
	(0.027)	(0.023)
Vaccine + Herd 70%	-0.003	0.020
	(0.026)	(0.023)
Vaccine + herd 80%	-0.024	-0.018
	(0.027)	(0.022)
Vaccine + Herd 60% + Current	-0.034	0.002
	(0.027)	(0.023)
Vaccine + Herd 70% + Current	-0.025	-0.012
	(0.027)	(0.022)
Vaccine + Herd 80% + Current	-0.037	-0.005
	(0.027)	(0.022)
Outcome range	{0,1}	{0,1}
Control outcome mean	0.57	0.22
Control outcome std. dev.	0.50	0.42
Observations	6,082	6,082
R^2	0.101	0.098

Table S27: Effect of different types of vaccine information treatment on demand for further vaccine information. All specifications include country $\times$ block fixed effects and (standardized) pre-treatment wait until vaccination as covariates (omitted to save space), weight observations by the inverse probability of treatment assignment, and are estimated using OLS. Robust standard errors are in parentheses. * denotes $p < 0.1$, ** denotes $p < 0.05$, *** denotes $p < 0.01$ from two-sided t tests.

	Outcome variable:	
	Requested more information	Visited PAHO website
	(1)	(2)
**Panel A: All countries pooled**		
Altruism	−0.009	−0.027*
	(0.017)	(0.015)
Economic recovery	−0.014	−0.028*
	(0.018)	(0.015)
Social approval	0.014	−0.006
	(0.018)	(0.015)
Outcome range	{0,1}	{0,1}
Control outcome mean	0.56	0.24
Control outcome std. dev.	0.50	0.43
Observations	6,082	6,082
R^2	0.097	0.090
**Panel B: Argentina**		
Altruism	0.014	−0.043
	(0.043)	(0.036)
Economic recovery	−0.000	−0.023
	(0.045)	(0.037)
Social approval	0.021	−0.026
	(0.043)	(0.036)
Outcome range	{0,1}	{0,1}
Control outcome mean	0.49	0.26
Control outcome std. dev.	0.50	0.44
Observations	1,019	1,019
R^2	0.085	0.135
**Panel C: Brazil**		
Altruism	−0.089**	−0.051
	(0.044)	(0.033)
Economic recovery	−0.057	−0.011
	(0.044)	(0.034)
Social approval	0.017	0.060
	(0.044)	(0.037)
Outcome range	{0,1}	{0,1}
Control outcome mean	0.51	0.19
Control outcome std. dev.	0.50	0.39
Observations	1,007	1,007
R^2	0.063	0.074
**Panel D: Chile**		
Altruism	−0.009	0.012
	(0.044)	(0.037)
Economic recovery	−0.018	−0.030
	(0.044)	(0.036)
Social approval	0.043	0.010
	(0.044)	(0.037)
Outcome range	{0,1}	{0,1}
Control outcome mean	0.51	0.23
Control outcome std. dev.	0.50	0.42
Observations	1,006	1,006
R^2	0.094	0.097
**Panel E: Colombia**		
Altruism	0.039	−0.010
	(0.043)	(0.038)
Economic recovery	0.081*	−0.023
	(0.043)	(0.037)
Social approval	0.087**	−0.021
	(0.043)	(0.037)
Outcome range	{0,1}	{0,1}
Control outcome mean	0.54	0.25
Control outcome std. dev.	0.50	0.43
Observations	1,011	1,011
R^2	0.087	0.068
**Panel F: México**		
Altruism	0.051	0.000
	(0.043)	(0.037)
Economic recovery	−0.033	−0.036
	(0.044)	(0.036)
Social approval	−0.014	−0.006
	(0.043)	(0.036)
Outcome range	{0,1}	{0,1}
Control outcome mean	0.58	0.22
Control outcome std. dev.	0.49	0.42
Observations	1,005	1,005
R^2	0.086	0.084
**Panel G: Perú**		
Altruism	−0.061	−0.071*
	(0.040)	(0.038)
Economic recovery	−0.058	−0.043
	(0.040)	(0.038)
Social approval	−0.068*	−0.050
	(0.040)	(0.039)
Outcome range	{0,1}	{0,1}
Control outcome mean	0.71	0.30
Control outcome std. dev.	0.46	0.46
Observations	1,034	1,034
R^2	0.105	0.092

Table S28: Effect of different types of motivational message on demand for further vaccine information. All specifications include country $\times$ block fixed effects and (standardized) pre-treatment wait until vaccination as covariates (omitted to save space) and are estimated using OLS. Robust standard errors are in parentheses. * denotes $p < 0.1$, ** denotes $p < 0.05$, *** denotes $p < 0.01$ from two-sided t tests.
