# Supplementary material for: Messages that increase COVID-19 vaccine acceptance: Evidence from online experiments in six Latin American countries
Source: PLoS One. 2021 Oct 28;16(10):e0259059. doi: 10.1371/journal.pone.0259059 (PMC8553119; doi:10.1371/journal.pone.0259059)
Supplement: S17 Appendix — (PDF) [file pone.0259059.s017.pdf]

S17 Population-weighted treatment effects

In estimating treatment effects, we did not apply population weights for each respondent to maximize the efficiency of our estimation of average treatment effects within a sample that was already nationally representative along several key dimensions. To more thoroughly examine how the results extend to the national hesitant population, we further weight our estimates in two ways (taking the product of inverse probability of treatment assignment weights and population weights, wherever relevant). First, within each country, we weight each respondent according to the relative frequency in the survey of the respondent's cell—defined by their age category, education, region, and gender—relative to the corresponding cell in the most recent available census. In other words, we reweight observations according to the joint distribution over these four variables in the population. Second, we instead apply rake weights to reweight observations according to the product of in-survey marginal distribution, relative to the national distribution, across the following variables: age category, education, region, gender, and (using data provided by Netquest) socioeconomic class. In each case, a small number of observations are dropped because weights could not be defined.

The results in Tables S29-S36 show that similar results apply. If anything, the positive effects of basic vaccine information on vaccine willingness and encouraging others are larger in magnitude once the population distribution is taken into account, although the effect on expected wait until vaccination once eligible is a little lower. The effects of the social approval treatment are also a little larger in magnitude. Unsurprisingly, the standard errors become larger once each type of weight is applied, although the core findings generally remain statistically significant for each type of population weight.

	Outcome variable:			
	Vaccine willingness scale	Willing to take a vaccine	Months would wait to get vaccinated (reversed)	Encourage others to get vaccinated
	(1)	(2)	(3)	(4)
**Panel A: All countries pooled**				
Any vaccine information	0.170^{***}	0.067^{***}	0.348^{***}	0.055^{***}
	(0.037)	(0.015)	(0.087)	(0.018)
Outcome range	[1,5]	{0,1}	[1,12]	{0,1}
Control outcome mean	3.14	0.38	5.84	0.50
Control outcome std. dev.	1.20	0.49	4.35	0.50
Observations	6,922	6,922	6,847	6,631
R^2	0.506	0.505	0.773	0.389
**Panel B: Argentina**				
Any vaccine information	0.268^{***}	0.075^{*}	0.473^{**}	0.083^{*}
	(0.094)	(0.042)	(0.216)	(0.046)
Outcome range	[1,5]	{0,1}	[1,12]	{0,1}
Control outcome mean	2.90	0.32	4.56	0.37
Control outcome std. dev.	1.12	0.47	4.42	0.48
Observations	1,156	1,156	1,146	1,105
R^2	0.489	0.511	0.824	0.424
**Panel C: Brazil**				
Any vaccine information	0.315^{***}	0.126^{***}	0.427^{**}	0.035
	(0.077)	(0.033)	(0.180)	(0.038)
Outcome range	[1,5]	{0,1}	[1,12]	{0,1}
Control outcome mean	3.15	0.35	5.87	0.43
Control outcome std. dev.	1.19	0.48	4.31	0.50
Observations	1,212	1,212	1,186	1,133
R^2	0.593	0.531	0.764	0.439
**Panel D: Chile**				
Any vaccine information	0.153	0.070^{**}	0.397^{*}	0.086^{**}
	(0.095)	(0.036)	(0.218)	(0.040)
Outcome range	[1,5]	{0,1}	[1,12]	{0,1}
Control outcome mean	2.94	0.32	4.97	0.43
Control outcome std. dev.	1.28	0.47	4.26	0.49
Observations	1,109	1,109	1,101	1,076
R^2	0.528	0.537	0.791	0.425
**Panel E: Colombia**				
Any vaccine information	0.226^{***}	0.094^{***}	0.441^{***}	0.077^{*}
	(0.082)	(0.032)	(0.143)	(0.040)
Outcome range	[1,5]	{0,1}	[1,12]	{0,1}
Control outcome mean	3.13	0.37	6.21	0.55
Control outcome std. dev.	1.24	0.48	4.28	0.50
Observations	1,130	1,130	1,119	1,084
R^2	0.506	0.526	0.834	0.408
**Panel F: México**				
Any vaccine information	-0.001	0.003	0.160	-0.002
	(0.099)	(0.043)	(0.224)	(0.050)
Outcome range	[1,5]	{0,1}	[1,12]	{0,1}
Control outcome mean	3.60	0.55	7.32	0.69
Control outcome std. dev.	1.20	0.50	4.03	0.46
Observations	1,098	1,098	1,094	1,071
R^2	0.453	0.470	0.692	0.284
**Panel G: Perú**				
Any vaccine information	0.062	0.031	0.195	0.052
	(0.085)	(0.036)	(0.263)	(0.044)
Outcome range	[1,5]	{0,1}	[1,12]	{0,1}
Control outcome mean	3.14	0.40	6.27	0.55
Control outcome std. dev.	1.06	0.49	4.22	0.50
Observations	1,217	1,217	1,201	1,162
R^2	0.422	0.440	0.686	0.299

Table S29: Effect of any vaccine information on vaccine willingness, using population cell weights. All specifications include country $\times$ block fixed effects and (standardized) pre-treatment wait until vaccination as covariates (omitted to save space), weight observations by the inverse probability of treatment assignment and population weights, and are estimated using OLS. Robust standard errors are in parentheses. * denotes $p < 0.1$, ** denotes $p < 0.05$, *** denotes $p < 0.01$ from two-sided t tests.

	Outcome variable:			
	Vaccine willingness scale	Willing to take a vaccine	Months would wait to get vaccinated (reversed)	Encourage others to get vaccinated
	(1)	(2)	(3)	(4)
Vaccine	0.122**	0.053**	0.276**	0.060**
	(0.054)	(0.022)	(0.119)	(0.025)
Vaccine + Biden	0.205***	0.090***	0.382**	0.080***
	(0.065)	(0.029)	(0.168)	(0.031)
Vaccine + Herd 60%	0.117*	0.048	0.211	0.028
	(0.067)	(0.029)	(0.168)	(0.035)
Vaccine + Herd 70%	0.202***	0.077***	0.561***	0.064**
	(0.072)	(0.029)	(0.162)	(0.032)
Vaccine + Herd 80%	0.161**	0.075**	0.313*	0.037
	(0.073)	(0.033)	(0.188)	(0.034)
Vaccine + Herd 60% + Current	0.229***	0.100***	0.441**	0.128***
	(0.067)	(0.032)	(0.218)	(0.031)
Vaccine + Herd 70% + Current	0.203***	0.081***	0.354**	0.092***
	(0.073)	(0.031)	(0.174)	(0.035)
Vaccine + Herd 80% + Current	0.150*	0.044	0.307*	−0.019
	(0.081)	(0.031)	(0.180)	(0.036)
Outcome range	[1,5]	{0,1}	[1,12]	{0,1}
Control outcome mean	3.14	0.38	5.84	0.50
Control outcome std. dev.	1.20	0.49	4.35	0.50
Observations	6,922	6,922	6,847	6,631
R^2	0.452	0.448	0.722	0.358

Table S30: Effect of different types of vaccine information on vaccine willingness, using population cell weights. All specifications include country $\times$ block fixed effects and (standardized) pre-treatment wait until vaccination as covariates (omitted to save space), weight observations by the inverse probability of treatment assignment and population weights, and are estimated using OLS. Robust standard errors are in parentheses. * denotes $p < 0.1$, ** denotes $p < 0.05$, *** denotes $p < 0.01$ from two-sided t tests.

	Outcome variable:			
	Vaccine willingness scale (1)	Willing to take a vaccine (2)	Months would wait to get vaccinated (reversed) (3)	Encourage others to get vaccinated (4)
Current	0.083	0.075*	0.256	0.110**
	(0.082)	(0.040)	(0.253)	(0.043)
Current rate below herd opinion	−0.001	0.023	0.026	0.012
	(0.073)	(0.035)	(0.211)	(0.041)
Current × Current rate below herd opinion	−0.060	−0.092*	−0.323	−0.118**
	(0.106)	(0.050)	(0.300)	(0.056)
Outcome range	[1,5]	{0,1}	[0,12]	{0,1}
Control outcome mean	3.39	0.48	6.16	0.54
Control outcome std. dev.	1.16	0.50	4.35	0.50
Observations	2,943	2,943	2,907	2,809
R^2	0.503	0.476	0.730	0.407

Table S31: The effect of being informed that the current rate of vaccination willingness in the population is above/below the rate required for herd immunity, using population cell weights. All specifications include country × block fixed effects and (standardized) pre-treatment wait until vaccination as covariates (omitted to save space), weight observations by the inverse probability of treatment assignment and population weights, and are estimated using OLS. Robust standard errors are in parentheses. * denotes $p < 0.1$, ** denotes $p < 0.05$, *** denotes $p < 0.01$ from two-sided t tests.

	Outcome variable:			
	Vaccine willingness scale (1)	Willing to take a vaccine (2)	Months would wait to get vaccinated (reversed) (3)	Encourage others to get vaccinated (4)
**Panel A: All countries pooled**				
Altruism	−0.002	0.003	0.119	−0.007
	(0.050)	(0.021)	(0.120)	(0.024)
Economic recovery	0.051	0.020	−0.020	0.026
	(0.045)	(0.020)	(0.116)	(0.022)
Social approval	0.143***	0.062***	0.339***	0.048**
	(0.045)	(0.021)	(0.130)	(0.023)
Outcome range	[1,5]	{0,1}	[1,12]	{0,1}
Control outcome mean	3.20	0.41	5.96	0.53
Control outcome std. dev.	1.16	0.49	4.42	0.50
Observations	6,922	6,922	6,847	6,631
R^2	0.453	0.457	0.734	0.349
**Panel B: Argentina**				
Altruism	−0.012	−0.011	0.247	−0.008
	(0.105)	(0.056)	(0.345)	(0.064)
Economic recovery	0.257**	0.117**	−0.047	0.054
	(0.116)	(0.059)	(0.309)	(0.062)
Social approval	0.189*	0.053	0.059	0.030
	(0.104)	(0.056)	(0.282)	(0.065)
Outcome range	[1,5]	{0,1}	[1,12]	{0,1}
Control outcome mean	3.07	0.37	5.45	0.47
Control outcome std. dev.	1.07	0.48	4.37	0.50
Observations	1,156	1,156	1,146	1,105
R^2	0.452	0.451	0.797	0.365
**Panel C: Brazil**				
Altruism	−0.083	−0.038	0.564**	0.021
	(0.120)	(0.054)	(0.277)	(0.053)
Economic recovery	0.101	−0.004	0.921***	0.039
	(0.088)	(0.042)	(0.264)	(0.048)
Social approval	0.144*	0.068*	1.044***	0.055
	(0.084)	(0.040)	(0.267)	(0.048)
Outcome range	[1,5]	{0,1}	[1,12]	{0,1}
Control outcome mean	3.30	0.42	5.24	0.45
Control outcome std. dev.	1.22	0.49	4.57	0.50
Observations	1,212	1,212	1,186	1,133
R^2	0.562	0.518	0.717	0.390
**Panel D: Chile**				
Altruism	0.159	0.080*	0.212	0.004
	(0.124)	(0.041)	(0.246)	(0.052)
Economic recovery	−0.012	0.041	0.100	0.035
	(0.104)	(0.038)	(0.255)	(0.053)
Social approval	0.187*	0.148***	0.836**	0.079
	(0.113)	(0.048)	(0.342)	(0.051)
Outcome range	[1,5]	{0,1}	[1,12]	{0,1}
Control outcome mean	2.97	0.30	4.81	0.49
Control outcome std. dev.	1.15	0.46	4.37	0.50
Observations	1,109	1,109	1,101	1,076
R^2	0.479	0.490	0.746	0.379
**Panel E: Colombia**				
Altruism	−0.067	−0.019	0.562**	0.025
	(0.111)	(0.041)	(0.225)	(0.047)
Economic recovery	0.019	−0.016	−0.080	−0.003
	(0.106)	(0.045)	(0.202)	(0.049)
Social approval	0.210*	0.052	0.399	0.073
	(0.115)	(0.047)	(0.277)	(0.052)
Outcome range	[1,5]	{0,1}	[1,12]	{0,1}
Control outcome mean	3.18	0.42	6.15	0.55
Control outcome std. dev.	1.25	0.49	4.62	0.50
Observations	1,130	1,130	1,119	1,084
R^2	0.461	0.465	0.780	0.360
**Panel F: México**				
Altruism	−0.032	−0.013	0.099	0.011
	(0.123)	(0.061)	(0.254)	(0.070)
Economic recovery	−0.125	−0.034	−0.109	0.047
	(0.120)	(0.054)	(0.356)	(0.058)
Social approval	0.018	−0.029	0.123	0.037
	(0.117)	(0.057)	(0.301)	(0.060)
Outcome range	[1,5]	{0,1}	[1,12]	{0,1}
Control outcome mean	3.64	0.59	7.51	0.66
Control outcome std. dev.	1.07	0.49	3.70	0.48
Observations	1,098	1,098	1,094	1,071
R^2	0.373	0.415	0.651	0.275
**Panel G: Perú**				
Altruism	−0.002	0.016	−0.961***	−0.095
	(0.124)	(0.052)	(0.362)	(0.062)
Economic recovery	0.071	0.022	−0.910***	−0.010
	(0.113)	(0.049)	(0.290)	(0.056)
Social approval	0.131	0.071	−0.456	0.025
	(0.123)	(0.056)	(0.373)	(0.057)
Outcome range	[1,5]	{0,1}	[1,12]	{0,1}
Control outcome mean	3.08	0.37	6.73	0.59
Control outcome std. dev.	1.10	0.48	4.25	0.49
Observations	1,217	1,217	1,201	1,162
R^2	0.368	0.399	0.685	0.308

Table S32: Effect of different types of motivational message on vaccine willingness, using population cell weights. All specifications include country $\times$ block fixed effects and (standardized) pre-treatment wait until vaccination as covariates (omitted to save space), weight observations by population weights, and are estimated using OLS. Robust standard errors are in parentheses. * denotes $p < 0.1$, ** denotes $p < 0.05$, *** denotes $p < 0.01$ from two-sided t tests.

	Outcome variable:			
	Vaccine willingness scale	Willing to take a vaccine	Months would wait to get vaccinated (reversed)	Encourage others to get vaccinated
	(1)	(2)	(3)	(4)
**Panel A: All countries pooled**				
Any vaccine information	0.168***	0.051***	0.347***	0.020
	(0.039)	(0.016)	(0.099)	(0.020)
Outcome range	[1,5]	{0,1}	[1,12]	{0,1}
Control outcome mean	3.17	0.41	6.02	0.54
Control outcome std. dev.	1.19	0.49	4.33	0.50
Observations	6,803	6,803	6,732	6,519
R^2	0.496	0.510	0.768	0.377
**Panel B: Argentina**				
Any vaccine information	0.253**	0.058	0.405**	0.089**
	(0.103)	(0.039)	(0.197)	(0.041)
Outcome range	[1,5]	{0,1}	[1,12]	{0,1}
Control outcome mean	2.92	0.35	5.06	0.43
Control outcome std. dev.	1.22	0.48	4.46	0.49
Observations	1,130	1,130	1,120	1,081
R^2	0.473	0.498	0.834	0.440
**Panel C: Brazil**				
Any vaccine information	0.233***	0.092***	0.392*	0.003
	(0.072)	(0.033)	(0.214)	(0.036)
Outcome range	[1,5]	{0,1}	[1,12]	{0,1}
Control outcome mean	3.24	0.40	5.93	0.49
Control outcome std. dev.	1.17	0.49	4.39	0.50
Observations	1,195	1,195	1,172	1,119
R^2	0.560	0.515	0.728	0.403
**Panel D: Chile**				
Any vaccine information	0.134	0.064*	0.473**	0.051
	(0.084)	(0.035)	(0.201)	(0.041)
Outcome range	[1,5]	{0,1}	[1,12]	{0,1}
Control outcome mean	2.91	0.31	4.83	0.46
Control outcome std. dev.	1.21	0.46	4.42	0.50
Observations	1,085	1,085	1,077	1,052
R^2	0.500	0.472	0.785	0.337
**Panel E: Colombia**				
Any vaccine information	0.138*	0.060**	0.360***	0.054
	(0.073)	(0.029)	(0.131)	(0.039)
Outcome range	[1,5]	{0,1}	[1,12]	{0,1}
Control outcome mean	3.17	0.39	6.19	0.54
Control outcome std. dev.	1.24	0.49	4.23	0.50
Observations	1,109	1,109	1,098	1,063
R^2	0.509	0.543	0.839	0.417
**Panel F: México**				
Any vaccine information	0.160	0.032	0.183	−0.058
	(0.112)	(0.042)	(0.288)	(0.057)
Outcome range	[1,5]	{0,1}	[1,12]	{0,1}
Control outcome mean	3.51	0.54	7.30	0.70
Control outcome std. dev.	1.15	0.50	3.89	0.46
Observations	1,072	1,072	1,069	1,046
R^2	0.467	0.542	0.715	0.347
**Panel G: Perú**				
Any vaccine information	0.096	0.017	0.371	0.039
	(0.078)	(0.036)	(0.237)	(0.042)
Outcome range	[1,5]	{0,1}	[1,12]	{0,1}
Control outcome mean	3.11	0.40	6.08	0.55
Control outcome std. dev.	1.04	0.49	4.27	0.50
Observations	1,212	1,212	1,196	1,158
R^2	0.421	0.432	0.695	0.310

Table S33: Effect of any vaccine information on vaccine willingness, using population rake weights. All specifications include country × block fixed effects and (standardized) pre-treatment wait until vaccination as covariates (omitted to save space), weight observations by the inverse probability of treatment assignment and population rake weights, and are estimated using OLS. Robust standard errors are in parentheses. * denotes $p < 0.1$, ** denotes $p < 0.05$, *** denotes $p < 0.01$ from two-sided t tests.

	Outcome variable:			
	Vaccine willingness scale	Willing to take a vaccine	Months would wait to get vaccinated (reversed)	Encourage others to get vaccinated
	(1)	(2)	(3)	(4)
Vaccine	0.113^{*}	0.042^{*}	0.388^{***}	0.051^{**}
	(0.058)	(0.023)	(0.143)	(0.025)
Vaccine + Biden	0.179^{***}	0.048^{*}	0.165	−0.008
	(0.063)	(0.026)	(0.179)	(0.035)
Vaccine + Herd 60%	0.121^{*}	0.043	0.115	0.012
	(0.070)	(0.037)	(0.173)	(0.040)
Vaccine + Herd 70%	0.177^{**}	0.063^{*}	0.560^{***}	0.042
	(0.070)	(0.033)	(0.208)	(0.034)
Vaccine + Herd 80%	0.182^{***}	0.053^{*}	0.166	−0.028
	(0.068)	(0.029)	(0.157)	(0.037)
Vaccine + Herd 60% + Current	0.184^{***}	0.068^{**}	0.330	0.083^{***}
	(0.065)	(0.030)	(0.208)	(0.032)
Vaccine + Herd 70% + Current	0.175^{**}	0.062^{**}	0.403^{**}	0.057
	(0.070)	(0.031)	(0.174)	(0.036)
Vaccine + Herd 80% + Current	0.182^{**}	0.038	0.592^{***}	−0.004
	(0.079)	(0.029)	(0.221)	(0.034)
Outcome range	[1,5]	{0,1}	[1,12]	{0,1}
Control outcome mean	3.17	0.41	6.02	0.54
Control outcome std. dev.	1.19	0.49	4.33	0.50
Observations	6,803	6,803	6,732	6,519
R^2	0.455	0.457	0.725	0.357

Table S34: Effect of different types of vaccine information on vaccine willingness, using population rake weights. All specifications include country × block fixed effects and (standardized) pre-treatment wait until vaccination as covariates (omitted to save space), weight observations by the inverse probability of treatment assignment and population rake weights, and are estimated using OLS. Robust standard errors are in parentheses. * denotes $p < 0.1$, ** denotes $p < 0.05$, *** denotes $p < 0.01$ from two-sided t tests.

	Outcome variable:			
	Vaccine willingness scale	Willing to take a vaccine	Months would wait to get vaccinated (reversed)	Encourage others to get vaccinated
	(1)	(2)	(3)	(4)
Current	0.076	0.066	0.295	0.066
	(0.081)	(0.042)	(0.220)	(0.046)
Current rate below herd opinion	0.054	0.043	0.165	−0.006
	(0.074)	(0.039)	(0.177)	(0.048)
Current × Current rate below herd opinion	−0.084	−0.093*	−0.243	−0.066
	(0.103)	(0.052)	(0.267)	(0.059)
Outcome range	[1,5]	{0,1}	[0,12]	{0,1}
Control outcome mean	3.37	0.48	6.32	0.53
Control outcome std. dev.	1.14	0.50	4.3	0.50
Observations	2,899	2,899	2,865	2,770
R^2	0.508	0.483	0.724	0.403

Table S35: The effect of being informed that the current rate of vaccination willingness in the population is above/below the rate required for herd immunity, using population rake weights. All specifications include country × block fixed effects and (standardized) pre-treatment wait until vaccination as covariates (omitted to save space), weight observations by the inverse probability of treatment assignment and population rake weights, and are estimated using OLS. Robust standard errors are in parentheses. * denotes $p < 0.1$, ** denotes $p < 0.05$, *** denotes $p < 0.01$ from two-sided t tests.

	Outcome variable:			
	Vaccine willingness scale	Willing to take a vaccine	Months would wait to get vaccinated (reversed)	Encourage others to get vaccinated
	(1)	(2)	(3)	(4)
**Panel A: All countries pooled**				
Altruism	0.016	−0.002	0.087	−0.011
	(0.049)	(0.021)	(0.117)	(0.024)
Economic recovery	0.061	0.010	−0.017	0.051**
	(0.047)	(0.020)	(0.123)	(0.023)
Social approval	0.172***	0.043**	0.297**	0.020
	(0.052)	(0.022)	(0.140)	(0.024)
Outcome range	[1,5]	{0,1}	[1,12]	{0,1}
Control outcome mean	3.24	0.43	6.28	0.56
Control outcome std. dev.	1.17	0.50	4.39	0.50
Observations	6,803	6,803	6,732	6,519
R^2	0.452	0.466	0.737	0.348
**Panel B: Argentina**				
Altruism	−0.146	−0.073	0.080	−0.045
	(0.117)	(0.055)	(0.252)	(0.061)
Economic recovery	0.239*	0.084	0.041	0.083
	(0.130)	(0.058)	(0.274)	(0.058)
Social approval	0.142	0.022	0.163	−0.006
	(0.123)	(0.058)	(0.282)	(0.061)
Outcome range	[1,5]	{0,1}	[1,12]	{0,1}
Control outcome mean	3.07	0.37	5.56	0.48
Control outcome std. dev.	1.08	0.48	4.42	0.50
Observations	1,130	1,130	1,120	1,081
R^2	0.417	0.437	0.805	0.371
**Panel C: Brazil**				
Altruism	−0.115	−0.034	0.364	−0.021
	(0.085)	(0.038)	(0.261)	(0.042)
Economic recovery	0.087	0.024	0.782***	0.035
	(0.075)	(0.034)	(0.237)	(0.042)
Social approval	0.188**	0.085**	1.111***	0.043
	(0.080)	(0.037)	(0.286)	(0.045)
Outcome range	[1,5]	{0,1}	[1,12]	{0,1}
Control outcome mean	3.31	0.41	5.45	0.46
Control outcome std. dev.	1.22	0.49	4.58	0.50
Observations	1,195	1,195	1,172	1,119
R^2	0.559	0.523	0.693	0.377
**Panel D: Chile**				
Altruism	0.148	0.077*	0.089	0.025
	(0.110)	(0.043)	(0.248)	(0.052)
Economic recovery	0.094	0.068	0.210	0.080
	(0.112)	(0.044)	(0.300)	(0.052)
Social approval	0.156	0.114**	0.628**	0.088*
	(0.103)	(0.045)	(0.312)	(0.051)
Outcome range	[1,5]	{0,1}	[1,12]	{0,1}
Control outcome mean	3.01	0.32	5.11	0.49
Control outcome std. dev.	1.16	0.47	4.49	0.50
Observations	1,085	1,085	1,077	1,052
R^2	0.456	0.447	0.733	0.313
**Panel E: Colombia**				
Altruism	−0.003	0.028	0.676***	0.050
	(0.108)	(0.042)	(0.210)	(0.047)
Economic recovery	0.037	0.015	0.034	0.039
	(0.094)	(0.038)	(0.185)	(0.046)
Social approval	0.133	0.027	0.259	0.059
	(0.094)	(0.039)	(0.247)	(0.049)
Outcome range	1-5	0-1	0-12	0-1
Control outcome mean	3.26	0.42	6.36	0.56
Control outcome std. dev.	1.20	0.49	4.49	0.50
Observations	1,109	1,109	1,098	1,063
R^2	0.458	0.480	0.791	0.360
**Panel F: México**				
Altruism	0.172	0.010	0.249	0.003
	(0.139)	(0.061)	(0.283)	(0.071)
Economic recovery	−0.065	−0.069	−0.137	0.038
	(0.130)	(0.053)	(0.346)	(0.063)
Social approval	0.285*	0.020	0.387	−0.049
	(0.150)	(0.058)	(0.350)	(0.063)
Outcome range	[1,5]	{0,1}	[1,12]	{0,1}
Control outcome mean	3.49	0.59	7.55	0.70
Control outcome std. dev.	1.20	0.49	3.81	0.46
Observations	1,072	1,072	1,069	1,046
R^2	0.433	0.499	0.721	0.364
**Panel G: Perú**				
Altruism	−0.057	−0.024	−1.044***	−0.075
	(0.107)	(0.051)	(0.364)	(0.057)
Economic recovery	0.034	−0.020	−1.014***	0.043
	(0.095)	(0.049)	(0.326)	(0.054)
Social approval	0.041	0.006	−0.818**	0.050
	(0.109)	(0.052)	(0.390)	(0.056)
Outcome range	[1,5]	{0,1}	[1,12]	{0,1}
Control outcome mean	3.14	0.40	6.95	0.57
Control outcome std. dev.	1.09	0.49	4.20	0.50
Observations	1,212	1,212	1,196	1,158
R^2	0.355	0.384	0.681	0.297

Table S36: Effect of different types of motivational message on vaccine willingness, using population rake weights. All specifications include country × block fixed effects and (standardized) pre-treatment wait until vaccination as covariates (omitted to save space), weight observations by population rake weights, and are estimated using OLS. Robust standard errors are in parentheses. * denotes $p < 0.1$, ** denotes $p < 0.05$, *** denotes $p < 0.01$ from two-sided t tests.
