# Supplementary material for: Messages that increase COVID-19 vaccine acceptance: Evidence from online experiments in six Latin American countries
Source: PLoS One. 2021 Oct 28;16(10):e0259059. doi: 10.1371/journal.pone.0259059 (PMC8553119; doi:10.1371/journal.pone.0259059)
Supplement: S18 Appendix — (PDF) [file pone.0259059.s018.pdf]

COLUMBIA UNIVERSITY
Institute for Social and Economic Research and Policy

Introduction

¡Hola!

Nos gustaría invitarlo a **participar en una encuesta para entender qué piensa la gente sobre la pandemia COVID-19**. Este estudio está siendo liderado por un grupo de investigadores de la Universidad de Columbia, Estados Unidos. Si usted desea participar, la encuesta le tomará aproximadamente **20 minutos**.

Su participación en el estudio es voluntaria. Además, una vez que termine la encuesta, la empresa Netquest lo recompensará. Sus respuestas se mantendrán estrictamente confidenciales. Usted puede terminar la encuesta en cualquier momento.

En caso de que tenga cualquier pregunta, duda, queja o comentario sobre este estudio, por favor contacte a John Marshall de la Universidad de Columbia, cuyo correo electrónico es jm4401@columbia.edu. Si tiene preguntas sobre sus derechos como sujeto de investigación, puede contactar al Comité de Ética Institucional de la Universidad de Columbia en el teléfono número +1 212 305 5883 o por correo electrónico askirboffice@columbia.edu.

Si desea participar en este estudio, haga click en el botón a continuación.

These page timer metrics will not be displayed to the recipient.
First Click: *0 seconds*
Last Click: *0 seconds*
Page Submit: *0 seconds*
Click Count: *0 clicks*

Screening/willingness questions

¿Hasta qué punto está usted de acuerdo o en desacuerdo?

Si una vacuna contra el COVID-19 estuviera disponible, yo me vacunaría.

- Muy en desacuerdo
- En desacuerdo
- Ni de acuerdo ni en desacuerdo
- De acuerdo
- Muy de acuerdo
- No estoy seguro

These page timer metrics will not be displayed to the recipient.
First Click: *0 seconds*
Last Click: *0 seconds*
Page Submit: *0 seconds*
Click Count: *0 clicks*

Si una vacuna contra el COVID-19 estuviera disponible para usted ahora, ¿cuántos meses esperaría antes de vacunarse?

- [ ] Numero de meses:
- Nunca tomaría una vacuna

These page timer metrics will not be displayed to the recipient.
First Click: *0 seconds*
Last Click: *0 seconds*
Page Submit: *0 seconds*
Click Count: *0 clicks*

Si una vacuna contra el COVID-19 estuviera disponible para todos ahora, ¿aproximadamente qué porcentaje de personas de su municipio piensa que se vacunarían?

0 25 50 75 100

These page timer metrics will not be displayed to the recipient.
First Click: *0 seconds*
Last Click: *0 seconds*
Page Submit: *0 seconds*
Click Count: *0 clicks*

Si una vacuna contra el COVID-19 estuviera disponible para todos ahora, ¿aproximadamente qué porcentaje de personas de su municipio piensa que se vacunarían **durante los primeros dos meses de su disponibilidad**?

0 25 50 75 100

These page timer metrics will not be displayed to the recipient.
First Click: *0 seconds*
Last Click: *0 seconds*
Page Submit: *0 seconds*
Click Count: *0 clicks*

Quota questions

¿Cuál es su edad? (años cumplidos)

¿En qué municipio vive usted?

Estado

Municipio

¿Cuál fue el último año de enseñanza que usted completó o aprobó?

Nivel de educación

Años completados o aprobados en este nivel

¿Cuál es su género?

- Femenino
- Masculino
- Otro

Background and attention questions

¿Su hogar tiene electricidad, agua corriente, o drenaje? Seleccione todas las que correspondan.

- Drenaje
- Electricidad
- Agua Corriente
- Ninguna

¿Cuál es su religión?

- Católico
- Protestante, Protestante Tradicional, o Protestante no Evangélico
- Evangélico o Pentecostal
- Islam
- Hinduista
- Budista
- Religiones Tradicionales o Nativas
- Ninguna
- Agnóstico o ateo
- Otra:

¿Cuál es la ciudad capital de ${e://Field/country}?

- Brasília
- Santiago
- Bogotá
- Buenos Aires
- Lima
- Ciudad de México

Pre-treatment questions

¿Con qué frecuencia consume **noticias sobre COVID-19** de las siguientes fuentes?

	Nunca	Una vez cada dos meses	Una vez al mes	Una vez cada dos semanas	Una vez por semana	Varias veces por semana	Diariamente
Periódicos	○	○	○	○	○	○	○
Televisión	○	○	○	○	○	○	○
Radio	○	○	○	○	○	○	○
Conversaciones con otros	○	○	○	○	○	○	○
WhatsApp	○	○	○	○	○	○	○
Redes sociales (e.j. Facebook, Twitter)	○	○	○	○	○	○	○
Sitios web de noticias	○	○	○	○	○	○	○

En su opinión, ¿qué tan serio es el tema del COVID-19 en ${e://Field/country}?

- Nada serio
- Poco serio
- Algo serio
- Muy serio
- No sé

Pensando en COVID-19, ¿qué tema le preocupa más?

- No poder educar a los jóvenes
- Salud mental
- Impacto económico
- Salud física
- Impacto político
- No estoy preocupado por el COVID-19
- No sé

Para que el COVID-19 pare de propagarse, ¿qué porcentaje de personas piensa que necesita vacunarse?

0 25 50 75 100

These page timer metrics will not be displayed to the recipient.

First Click: *0 seconds*

Last Click: *0 seconds*

Page Submit: *0 seconds*

Click Count: *0 clicks*

¿Hasta qué punto está usted de acuerdo o en desacuerdo con las siguientes declaraciones?

	Muy en desacuerdo	En desacuerdo	Ni de acuerdo ni en desacuerdo	De acuerdo	Muy de acuerdo
Confío en que expertos médicos internacionales desarrollen vacunas seguras y eficaces.	○	○	○	○	○
Vacunarme es una buena manera para protegerme de enfermedades.	○	○	○	○	○
Confío que el gobierno determine si las vacunas son seguras y eficaces.	○	○	○	○	○
Vacunarme contra enfermedades que pueden ser graves es importante para la salud de los demás en mi comunidad.	○	○	○	○	○
Generalmente, sigo las indicaciones de mi médico sobre vacunaciones.	○	○	○	○	○

¿Alguna vez ha rechazado una vacuna recomendada para usted o sus hijos?

- ○ No
- ○ Sí
- ○ No sé
- ○ Prefiero no decir
- ○ No aplica

¿Cuáles de las siguentes opciones describe por qué duda en tomar una vacuna en contra del COVID-19? Seleccione todas las que correspondan.

- ☐ Ya tuve COVID-19
- ☐ Temo que las vacunas están siendo desarolladas demasiado rápido
- ☐ Mi riesgo de contraer el COVID-19 es tan bajo que no necesito la vacuna
- ☐ Estoy preocupado por los efectos secundarios
- ☐ No creo que las vacunas sean efectivas contra el COVID-19
- ☐ No confió en el gobierno
- ☐ Temo que la vacuna me dará COVID-19
- ☐ Prefiero adquirir inmunidad tras contraer COVID-19, sin necesidad de una vacuna
- ☐ Temo que no podré pagar una vacuna para el COVID-19
- ☐ Estoy en contra de las vacunas
- ☐ __________ Otra:

¿Sufre de algunas de las siguientes enfermedades crónicas? Seleccione todas las que correspondan.

- ☐ Ninguna
- ☐ Enfermedades cardiovasculares
- ☐ Enfermedades autoinmunes
- ☐ Diabetes
- ☐ Enfermedad pulmonar obstructiva crónica
- ☐ Obesidad
- ☐ Prefiero no decir

¿Ha sido diagnosticado con COVID-19?

- ○ No, nunca he sido diagnosticado con COVID-19
- ○ Sí, actualmente tengo COVID-19
- ○ Sí, he tenido COVID-19 en el pasado
- ○ Prefiero no decir

¿Conoce a alguien que se enfermó gravemente o falleció debido a COVID-19?

- No
- Sí
- No sé

¿Considera usted que su situación económica personal es peor, igual, o mejor que antes de la pandemia?

- Mucho peor
- Peor
- Igual
- Mejor
- Mucho mejor
- No sé

En su opinión, ¿cuán prioritario es para el gobierno distribuir una vacuna en su municipio?

- No es una prioridad
- Una prioridad baja
- Una prioridad media
- Una prioridad alta
- Una máxima prioridad
- No sé

Hoy en día cuando se habla de **tendencias políticas**, mucha gente habla de aquellos que simpatizan más con la izquierda o con la derecha. Según el sentido que tengan para usted los términos "izquierda" y "derecha" cuando piensa sobre su punto de vista político, ¿dónde se encontraría usted en esta escala?

0 (extrema izquierda)	1	2	3	4	5 (centro)	6	7	8	9	10 (extrema derecha)
○	○	○	○	○	○	○	○	○	○	○

Con respecto al manejo de la pandemia, ¿qué tan satisfecho está usted con las siguientes autoridades?

	Nada satisfecho	No satisfecho	Ni satisfecho ni insatisfecho	Satisfecho	Muy satisfecho
Presidente ${e://Field/president}	○	○	○	○	○
${e://Field/health_ministry}	○	○	○	○	○
${e://Field/mayor_gender} de su municipalidad	○	○	○	○	○

Si hubiese una **elección presidencial** mañana, ¿votaría usted a favor del partido o alguien de la coalición del Presidente ${e://Field/president}?

- ○ No, votaría por un candidato de la oposición
- ○ Sí
- ○ No votaría
- ○ No sé

¿Por cuál partido de la oposición votaría en una elección presidencial?

- PAN
- PRI
- PRD
- PT
- PVEM
- MC
- PES
- RSP
- FSP
- ____________ Otro:
- No sé

Si la **elección para ${e://Field/mayor} en su municipio** fuese mañana, ¿votaría usted a favor del partido o alguien de la coalición del actual ${e://Field/mayor}?

- » Sí
- » No, votaría por un candidato de la oposición
- » No votaría
- » No sé

¿Por cuál partido de la oposición votaría en las elecciones locales (o en la elección para ${e://Field/mayor})?

- » PAN
- » PRI
- » PRD
- » PT
- » PVEM
- » MC
- » PES
- » RSP
- » FSP
- ________ » Otro:
- » No sé

¿Cuánta confianza tiene en las siguientes personas e instituciones?

	Nada de confianza	Poca confianza	Algo de confianza	Mucha confianza	No sé
Presidente ${e://Field/president}	○	○	○	○	○
${e://Field/mayor_gender_2} de mi municipio	○	○	○	○	○
${e://Field/health_ministry}	○	○	○	○	○
${e://Field/medical_association}	○	○	○	○	○
${e://Field/left_newspaper}	○	○	○	○	○
${e://Field/right_newspaper}	○	○	○	○	○
${e://Field/religious_leader}	○	○	○	○	○

¿Cuánta confianza tiene en las siguientes instituciones y organizaciones?

	Nada de confianza	Poca confianza	Algo de confianza	Mucha confianza	No sé
Sus profesionales locales de salud	○	○	○	○	○
Organizaciones de la sociedad civil	○	○	○	○	○
Las fuerzas armadas de ${e://Field/country}	○	○	○	○	○

¿Cuánta confianza tiene en los gobiernos actuales de los siguientes países?

	Nada de confianza	Poca confianza	Algo de confianza	Mucha confianza	No sé
China	○	○	○	○	○
Estados Unidos bajo Donald Trump	○	○	○	○	○
Estados Unidos bajo Joe Biden	○	○	○	○	○
Reino Unido	○	○	○	○	○
Rusia	○	○	○	○	○

¿Hasta qué punto cree usted que reunirse con personas fuera de su familia, en lugares cerrados, contribuye a propagar el COVID-19?

- ○ Nada
- ○ Un poco
- ○ Algo
- ○ Mucho
- ○ No sé

Haga de cuenta que usted tiene la posibilidad de lanzar una moneda justa. Si la moneda cae en cara, obtendrá ${e://Field/risk_currency}. Si no, obtendrá 0 ${e://Field/currency}. Eso significa que tiene una probabilidad del 50% de obtener los ${e://Field/risk_currency} y una probabilidad del 50% de obtener nada. **¿Qué prefiere: tomar el riesgo, o recibir una cantidad segura?** Seleccione una respuesta para cada una de las opciones.

	Tomar el riesgo	Tomar el dinero seguro
Tomar el riesgo de recibir ${e://Field/risk_currency} con una probabilidad del 50% **o recibir ${e://Field/sure_1_currency} asegurados**	○	○
Tomar el riesgo de recibir ${e://Field/risk_currency} con una probabilidad del 50% **o ${e://Field/sure_2_currency} asegurados**	○	○
Tomar el riesgo de recibir ${e://Field/risk_currency} con una probabilidad del 50% **o ${e://Field/sure_3_currency} asegurados**	○	○
Tomar el riesgo de recibir ${e://Field/risk_currency} con una probabilidad del 50% **o ${e://Field/sure_4_currency} asegurados**	○	○
Tomar el riesgo de recibir ${e://Field/risk_currency} con una probabilidad del 50% **o ${e://Field/sure_5_currency} asegurados**	○	○

Haga de cuenta que usted tiene la posibilidad de obtener ${e://Field/sure_1_currency} en este momento, o una cantidad superior dentro de un año. **¿Qué prefiere recibir: los ${e://Field/sure_1_currency} en este momento o la cantidad superior en un año?** Seleccione una respuesta para cada una de las opciones.

	De Acuerdo	En Desacuerdo
Prefiero ${e://Field/sure_1_currency} en este momento a ${e://Field/discount_1_currency} dentro de un año	○	○
Prefiero ${e://Field/sure_1_currency} en este momento a ${e://Field/discount_2_currency} dentro de un año	○	○
Prefiero ${e://Field/sure_1_currency} en este momento a ${e://Field/discount_3_currency} dentro de un año	○	○
Prefiero ${e://Field/sure_1_currency} en este momento a ${e://Field/discount_4_currency} dentro de un año	○	○

Suponga que a usted le dan ${e://Field/sure_1_currency} y tiene que decidir cuanta

plata donar a una familia con necesidad en su comunidad. ¿Cuánto de esos ${e://Field/sure_1_currency} donaría a esta familia?

¿Qué tan importante para usted es recibir el respeto y el reconocimiento de otros en su comunidad?

- Nada importante
- Poco importante
- Algo importante
- Muy importante

¿Cuánta influencia cree usted que tiene con otras personas de su comunidad?

- Nada de influencia
- Poca influencia
- Algo de influencia
- Mucha influencia

Information treatment T0 - Control

Los países de Latinoamérica están comenzando a distribuir sus primeras dosis de vacunas.

These page timer metrics will not be displayed to the recipient.
First Click: *0 seconds*
Last Click: *0 seconds*
Page Submit: *0 seconds*
Click Count: *0 clicks*

Information treatment T1 - Health only

Los países de Latinoamerica están comenzando a distribuir sus primeras dosis de vacunas.

La siguiente pantalla proporcionará **información importante sobre estas vacunas** contra el COVID-19.

These page timer metrics will not be displayed to the recipient.
First Click: *0 seconds*
Last Click: *0 seconds*
Page Submit: *0 seconds*
Click Count: *0 clicks*

Las vacunas están diseñadas para **prevenir enfermedades**.

Después de **pruebas exhaustivas realizadas por expertos médicos**, se ha aprobado el uso de varias vacunas contra el COVID-19 en diferentes países.

Las pruebas clínicas han demonstrado que las vacunas son **seguras y altamente eficaces** en prevenir infecciones leves y graves de COVID-19. Los **efectos secundarios son generalmente menores** y no se puede contraer COVID-19 de una vacuna.

¿Cuáles de las siguientes afirmaciones son **ciertas** sobre las vacunas nuevas contra el COVID-19? Seleccione todas las que correspondan.

- ☐ Ningún país ha aprobado el uso de vacunas contra el COVID-19
- ☐ No se sabe si hay efectos secundarios graves de tomar una vacuna contra el COVID-19
- ☐ No es posible contraer COVID-19 de una vacuna
- ☐ Las pruebas muestran que las vacunas son altamente eficaces en prevenir infecciones de COVID-19
- ☐ Las vacunas se han sometido a exhaustivas pruebas clínicas
- ☐ Ninguno de los anteriores

These page timer metrics will not be displayed to the recipient.
First Click: *0 seconds*
Last Click: *0 seconds*
Page Submit: *0 seconds*
Click Count: *0 clicks*

Information treatment T2 - Health and herd 60%

Los países de Latinoamerica están comenzando a distribuir sus primeras dosis de vacunas.

La siguiente pantalla proporcionará **información importante sobre estas vacunas** contra el COVID-19.

These page timer metrics will not be displayed to the recipient.
First Click: *0 seconds*
Last Click: *0 seconds*
Page Submit: *0 seconds*
Click Count: *0 clicks*

Las vacunas están diseñadas para **prevenir enfermedades**.

Después de **pruebas exhaustivas realizadas por expertos médicos**, se ha aprobado el uso de varias vacunas contra el COVID-19 en diferentes países.

Las pruebas clínicas han demonstrado que las vacunas son **seguras y altamente eficaces** en prevenir infecciones leves y graves de COVID-19. Los **efectos secundarios son generalmente menores** y no se puede contraer COVID-19 de una vacuna.

¿Cuáles de las siguientes afirmaciones son **ciertas** sobre las vacunas nuevas contra el COVID-19? Seleccione todas las que correspondan.

- ☐ Ningún país ha aprobado el uso de vacunas contra el COVID-19
- ☐ Las pruebas muestran que las vacunas son altamente eficaces en prevenir infecciones de COVID-19
- ☐ No es posible contraer COVID-19 de una vacuna
- ☐ Las vacunas se han sometido a exhaustivas pruebas clínicas
- ☐ No se sabe si hay efectos secundarios graves de tomar una vacuna contra el COVID-19
- ☐ Ninguno de los anteriores

These page timer metrics will not be displayed to the recipient.
First Click: *0 seconds*
Last Click: *0 seconds*
Page Submit: *0 seconds*
Click Count: *0 clicks*

Si suficientes personas se vacunan contra el COVID-19, el Coronavirus dejará de propagararse.

Algunos expertos dicen que **al menos 60% de las personas necesitan vacunarse para evitar la propagación del Coronavirus**.

¿Es cierto que algunos expertos dicen que al menos 60% de las personas necesitarán vacunarse para evitar la propagación del COVID-19?

- ○ Es cierto
- ○ Es falso
- ○ No sé

These page timer metrics will not be displayed to the recipient.
First Click: *0 seconds*
Last Click: *0 seconds*
Page Submit: *0 seconds*
Click Count: *0 clicks*

Information treatment T3 - Health and herd 70%

Los países de Latinoamerica están comenzando a distribuir sus primeras dosis de vacunas.

La siguiente pantalla proporcionará **información importante sobre estas vacunas** contra el COVID-19.

These page timer metrics will not be displayed to the recipient.
First Click: *0 seconds*
Last Click: *0 seconds*
Page Submit: *0 seconds*
Click Count: *0 clicks*

Las vacunas están diseñadas para **prevenir enfermedades**.

Después de **pruebas exhaustivas realizadas por expertos médicos**, se ha aprobado el uso de varias vacunas contra el COVID-19 en diferentes países.

Las pruebas clínicas han demonstrado que las vacunas son **seguras y altamente eficaces** en prevenir infecciones leves y graves de COVID-19. Los **efectos secundarios son generalmente menores** y no se puede contraer COVID-19 de una vacuna.

¿Cuáles de las siguientes afirmaciones son **ciertas** sobre las vacunas nuevas contra el COVID-19? Seleccione todas las que correspondan.

- ☐ Las pruebas muestran que las vacunas son altamente eficaces en prevenir infecciones de COVID-19
- ☐ No se sabe si hay efectos secundarios graves de tomar una vacuna contra el COVID-19
- ☐ No es posible contraer COVID-19 de una vacuna
- ☐ Ningún país ha aprobado el uso de vacunas contra el COVID-19
- ☐ Las vacunas se han sometido a exhaustivas pruebas clínicas
- ☐ Ninguno de los anteriores

These page timer metrics will not be displayed to the recipient.
First Click: *0 seconds*
Last Click: *0 seconds*
Page Submit: *0 seconds*
Click Count: *0 clicks*

Si suficientes personas se vacunan contra el COVID-19, el Coronavirus dejará de propagararse.

Algunos expertos dicen que **al menos 70% de las personas necesitan vacunarse para evitar la propagación del Coronavirus**.

¿Es cierto que algunos expertos dicen que al menos 70% de las personas necesitarán vacunarse para evitar la propagación del COVID-19?

- ○ Es cierto
- ○ Es falso
- ○ No sé

These page timer metrics will not be displayed to the recipient.
First Click: *0 seconds*
Last Click: *0 seconds*
Page Submit: *0 seconds*
Click Count: *0 clicks*

Information treatment T4 - Health and herd 80%

Los países de Latinoamerica están comenzando a distribuir sus primeras dosis de vacunas.

La siguiente pantalla proporcionará **información importante sobre estas vacunas** contra el COVID-19.

These page timer metrics will not be displayed to the recipient.
First Click: *0 seconds*
Last Click: *0 seconds*
Page Submit: *0 seconds*
Click Count: *0 clicks*

Las vacunas están diseñadas para **prevenir enfermedades**.

Después de **pruebas exhaustivas realizadas por expertos médicos**, se ha aprobado el uso de varias vacunas contra el COVID-19 en diferentes países.

Las pruebas clínicas han demonstrado que las vacunas son **seguras y altamente eficaces** en prevenir infecciones leves y graves de COVID-19. Los **efectos secundarios son generalmente menores** y no se puede contraer COVID-19 de una vacuna.

¿Cuáles de las siguientes afirmaciones son **ciertas** sobre las vacunas nuevas contra el COVID-19? Seleccione todas las que correspondan.

- ☐ Ningún país ha aprobado el uso de vacunas contra el COVID-19
- ☐ No es posible contraer COVID-19 de una vacuna
- ☐ No se sabe si hay efectos secundarios graves de tomar una vacuna contra el COVID-19
- ☐ Las vacunas se han sometido a exhaustivas pruebas clínicas
- ☐ Las pruebas muestran que las vacunas son altamente eficaces en prevenir infecciones de COVID-19
- ☐ Ninguno de los anteriores

These page timer metrics will not be displayed to the recipient.
First Click: *0 seconds*
Last Click: *0 seconds*
Page Submit: *0 seconds*
Click Count: *0 clicks*

Si suficientes personas se vacunan contra el COVID-19, el Coronavirus dejará de propagararse.

Algunos expertos dicen que **al menos 80% de las personas necesitan vacunarse para evitar la propagación del Coronavirus**.

¿Es cierto que algunos expertos dicen que al menos 80% de las personas necesitarán vacunarse para evitar la propagación del COVID-19?

- ○ Es cierto
- ○ Es falso
- ○ No sé

These page timer metrics will not be displayed to the recipient.
First Click: *0 seconds*
Last Click: *0 seconds*
Page Submit: *0 seconds*
Click Count: *0 clicks*

Information treatment T5 - Health and herd 60% and current level

Los países de Latinoamerica están comenzando a distribuir sus primeras dosis de vacunas.

La siguiente pantalla proporcionará **información importante sobre estas vacunas** contra el COVID-19.

These page timer metrics will not be displayed to the recipient.
First Click: *0 seconds*
Last Click: *0 seconds*
Page Submit: *0 seconds*
Click Count: *0 clicks*

Las vacunas están diseñadas para **prevenir enfermedades**.

Después de **pruebas exhaustivas realizadas por expertos médicos**, se ha aprobado el uso de varias vacunas contra el COVID-19 en diferentes países.

Las pruebas clínicas han demonstrado que las vacunas son **seguras y altamente eficaces** en prevenir infecciones leves y graves de COVID-19. Los **efectos secundarios son generalmente menores** y no se puede contraer COVID-19 de una vacuna.

¿Cuáles de las siguientes afirmaciones son **ciertas** sobre las vacunas nuevas contra el COVID-19? Seleccione todas las que correspondan.

- ☐ Las vacunas se han sometido a exhaustivas pruebas clínicas
- ☐ No se sabe si hay efectos secundarios graves de tomar una vacuna contra el COVID-19
- ☐ Las pruebas muestran que las vacunas son altamente eficaces en prevenir infecciones de COVID-19
- ☐ No es posible contraer COVID-19 de una vacuna
- ☐ Ningún país ha aprobado el uso de vacunas contra el COVID-19
- ☐ Ninguno de los anteriores

These page timer metrics will not be displayed to the recipient.
First Click: *0 seconds*
Last Click: *0 seconds*
Page Submit: *0 seconds*
Click Count: *0 clicks*

Si suficientes personas se vacunan contra el COVID-19, el Coronavirus dejará de propagarse.

Algunos expertos dicen que **al menos 60% de las personas necesitan vacunarse para evitar la propagación del Coronavirus**.

Datos recientes indican que **${e://Field/current_willingness} de las personas en ${e://Field/country} actualmente dicen que se vacunarían** contra el COVID-19.

En ${e://Field/country}, ¿están más o menos personas dispuestas a tomar una vacuna que el 60% de las personas que algunos expertos dicen que necesitarán tomar la vacuna para evitar la propagación del COVID-19?

- **Más del 60%** están dispuestos a tomar una vacuna
- **Menos del 60%** están dispuestos a tomar una vacuna
- No sé

These page timer metrics will not be displayed to the recipient.
First Click: *0 seconds*
Last Click: *0 seconds*
Page Submit: *0 seconds*
Click Count: *0 clicks*

Information treatment T6 - Health and herd 70% and current level

Los países de Latinoamerica están comenzando a distribuir sus primeras dosis de vacunas.

La siguiente pantalla proporcionará **información importante sobre estas vacunas** contra el COVID-19.

These page timer metrics will not be displayed to the recipient.
First Click: *0 seconds*
Last Click: *0 seconds*
Page Submit: *0 seconds*
Click Count: *0 clicks*

Las vacunas están diseñadas para **prevenir enfermedades**.

Después de **pruebas exhaustivas realizadas por expertos médicos**, se ha aprobado el uso de varias vacunas contra el COVID-19 en diferentes países.

Las pruebas clínicas han demonstrado que las vacunas son **seguras y altamente**

eficaces en prevenir infecciones leves y graves de COVID-19. Los **efectos secundarios son generalmente menores** y no se puede contraer COVID-19 de una vacuna.

¿Cuáles de las siguientes afirmaciones son **ciertas** sobre las vacunas nuevas contra el COVID-19? Seleccione todas las que correspondan.

- ☐ Ningún país ha aprobado el uso de vacunas contra el COVID-19
- ☐ No es posible contraer COVID-19 de una vacuna
- ☐ Las pruebas muestran que las vacunas son altamente eficaces en prevenir infecciones de COVID-19
- ☐ No se sabe si hay efectos secundarios graves de tomar una vacuna contra el COVID-19
- ☐ Las vacunas se han sometido a exhaustivas pruebas clínicas
- ☐ Ninguno de los anteriores

These page timer metrics will not be displayed to the recipient.
First Click: *0 seconds*
Last Click: *0 seconds*
Page Submit: *0 seconds*
Click Count: *0 clicks*

Si suficientes personas se vacunan contra el COVID-19, el Coronavirus dejará de propagarse.

Algunos expertos dicen que **al menos 70% de las personas necesitan vacunarse para evitar la propagación del Coronavirus**.

Datos recientes indican que **${e://Field/current_willingness} de las personas en ${e://Field/country} actualmente dicen que se vacunarían** contra el COVID-19.

En ${e://Field/country}, ¿están más o menos personas dispuestas a tomar una vacuna que el 70% de las personas que algunos expertos dicen que necesitarán tomar la vacuna para evitar la propagación del COVID-19?

- ○ **Más del 70%** están dispuestos a tomar una vacuna
- ○ **Menos del 70%** están dispuestos a tomar una vacuna
- ○ No sé

These page timer metrics will not be displayed to the recipient.
First Click: *0 seconds*
Last Click: *0 seconds*
Page Submit: *0 seconds*
Click Count: *0 clicks*

Information treatment T7 - Health and herd 80% and current level

Los países de Latinoamerica están comenzando a distribuir sus primeras dosis de vacunas.

La siguiente pantalla proporcionará **información importante sobre estas vacunas** contra el COVID-19.

These page timer metrics will not be displayed to the recipient.
First Click: *0 seconds*
Last Click: *0 seconds*
Page Submit: *0 seconds*
Click Count: *0 clicks*

Las vacunas están diseñadas para **prevenir enfermedades**.

Después de **pruebas exhaustivas realizadas por expertos médicos**, se ha aprobado el uso de varias vacunas contra el COVID-19 en diferentes países.

Las pruebas clínicas han demonstrado que las vacunas son **seguras y altamente**

eficaces en prevenir infecciones leves y graves de COVID-19. Los **efectos secundarios son generalmente menores** y no se puede contraer COVID-19 de una vacuna.

¿Cuáles de las siguientes afirmaciones son **ciertas** sobre las vacunas nuevas contra el COVID-19? Seleccione todas las que correspondan.

- ☐ Ningún país ha aprobado el uso de vacunas contra el COVID-19
- ☐ No es posible contraer COVID-19 de una vacuna
- ☐ Las pruebas muestran que las vacunas son altamente eficaces en prevenir infecciones de COVID-19
- ☐ No se sabe si hay efectos secundarios graves de tomar una vacuna contra el COVID-19
- ☐ Las vacunas se han sometido a exhaustivas pruebas clínicas
- ☐ Ninguno de los anteriores

These page timer metrics will not be displayed to the recipient.
First Click: *0 seconds*
Last Click: *0 seconds*
Page Submit: *0 seconds*
Click Count: *0 clicks*

Si suficientes personas se vacunan contra el COVID-19, el Coronavirus dejará de propagarse.

Algunos expertos dicen que **al menos 80% de las personas necesitan vacunarse para evitar la propagación del Coronavirus**.

Datos recientes indican que **${e://Field/current_willingness} de las personas en ${e://Field/country} actualmente dicen que se vacunarían** contra el COVID-19.

En ${e://Field/country}, ¿están más o menos personas dispuestas a tomar una vacuna que el 80% de las personas que algunos expertos dicen que necesitarán tomar la vacuna para evitar la propagación del COVID-19?

- **Menos del 80%** están dispuestos a tomar una vacuna
- **Más del 80%** están dispuestos a tomar una vacuna
- No sé

These page timer metrics will not be displayed to the recipient.
First Click: *0 seconds*
Last Click: *0 seconds*
Page Submit: *0 seconds*
Click Count: *0 clicks*

Information treatment T8 - Health and Biden vaccinated

Los países de Latinoamerica están comenzando a distribuir sus primeras dosis de vacunas.

La siguiente pantalla proporcionará **información importante sobre estas vacunas** contra el COVID-19.

These page timer metrics will not be displayed to the recipient.
First Click: *0 seconds*
Last Click: *0 seconds*
Page Submit: *0 seconds*
Click Count: *0 clicks*

Las vacunas están diseñadas para **prevenir enfermedades**.

Después de **pruebas exhaustivas realizadas por expertos médicos**, se ha aprobado el uso de varias vacunas contra el COVID-19 en diferentes países.

Las pruebas clínicas han demonstrado que las vacunas son **seguras y altamente**

eficaces en prevenir infecciones leves y graves de COVID-19. Los **efectos secundarios son generalmente menores** y no se puede contraer COVID-19 de una vacuna.

¿Cuáles de las siguientes afirmaciones son **ciertas** sobre las vacunas nuevas contra el COVID-19? Seleccione todas las que correspondan.

- ☐ Las vacunas se han sometido a exhaustivas pruebas clínicas
- ☐ No es posible contraer COVID-19 de una vacuna
- ☐ Ningún país ha aprobado el uso de vacunas contra el COVID-19
- ☐ Las pruebas muestran que las vacunas son altamente eficaces en prevenir infecciones de COVID-19
- ☐ No se sabe si hay efectos secundarios graves de tomar una vacuna contra el COVID-19
- ☐ Ninguno de los anteriores

These page timer metrics will not be displayed to the recipient.
First Click: *0 seconds*
Last Click: *0 seconds*
Page Submit: *0 seconds*
Click Count: *0 clicks*

Hace algunas semanas, **el presidente Joe Biden recibió, de manera segura, una vacuna** contra el COVID-19 en los Estados Unidos.

These page timer metrics will not be displayed to the recipient.
First Click: *0 seconds*
Last Click: *0 seconds*
Page Submit: *0 seconds*
Click Count: *0 clicks*

No motivation control M0

¿Se ha aprobado el uso de una vacuna contra el COVID-19 en algún país?

- Sí
- No
- No sé

These page timer metrics will not be displayed to the recipient.
First Click: *0 seconds*
Last Click: *0 seconds*
Page Submit: *0 seconds*
Click Count: *0 clicks*

Economic motivation M1

Cuanto más rápido ${e://Field/country} pueda detener la propagación de COVID-19, más rápido las personas volverán a trabajar.

Si usted se vacuna contra el COVID-19, **ayudará a que la economía se recupere**.

These page timer metrics will not be displayed to the recipient.
First Click: *0 seconds*
Last Click: *0 seconds*
Page Submit: *0 seconds*
Click Count: *0 clicks*

¿Se ha aprobado el uso de una vacuna contra el COVID-19 en algún país?

- Sí
- No
- No sé

These page timer metrics will not be displayed to the recipient.
First Click: *0 seconds*
Last Click: *0 seconds*
Page Submit: *0 seconds*
Click Count: *0 clicks*

Social incentive motivation M2

Vacunarse contra el COVID-19 demuestra que usted se preocupa por los demás en su comunidad.

Si usted se vacuna contra el COVID-19, **será respetado por las personas en su comunidad**.

These page timer metrics will not be displayed to the recipient.
First Click: *0 seconds*
Last Click: *0 seconds*
Page Submit: *0 seconds*
Click Count: *0 clicks*

¿Se ha aprobado el uso de una vacuna contra el COVID-19 en algún país?

- No
- Sí
- No sé

These page timer metrics will not be displayed to the recipient.
First Click: *0 seconds*
Last Click: *0 seconds*
Page Submit: *0 seconds*
Click Count: *0 clicks*

Altruism motivation M3

Vacunarse contra el COVID-19 ayuda a detener la propagación del COVID-19 y así evita que los más vulnerables se enfermen.

Si usted se vacuna contra el COVID-19, **ayudará a mantener saludables a otros en su comunidad**.

These page timer metrics will not be displayed to the recipient.
First Click: *0 seconds*
Last Click: *0 seconds*
Page Submit: *0 seconds*
Click Count: *0 clicks*

¿Se ha aprobado el uso de una vacuna contra el COVID-19 en algún país?

- No
- Sí
- No sé

These page timer metrics will not be displayed to the recipient.
First Click: *0 seconds*
Last Click: *0 seconds*
Page Submit: *0 seconds*
Click Count: *0 clicks*

Treatment comprehension

En las pruebas clínicas de las vacunas contra el COVID-19, ¿qué tipo de efectos secundarios han tenido los participantes generalmente?

- No han tenido efectos secundarios
- Efectos secundarios menores
- Efectos secundarios graves
- No sé

These page timer metrics will not be displayed to the recipient.
First Click: *0 seconds*
Last Click: *0 seconds*
Page Submit: *0 seconds*
Click Count: *0 clicks*

Ahora nos gustaría reevaluar sus puntos de vista sobre tomar una vacuna contra el COVID-19.

Response to treatment

En base a la información que acaba de recibir, ¿se han contestado algunas de las dudas que tenía sobre las vacunas contra el COVID-19? Seleccione todas las que correspondan.

- ☐ Ya no pienso que mi riesgo de contraer el COVID-19 es tan bajo que no necesito la vacuna
- ☐ Aunque ya tuve COVID-19, ahora prefiero tomar una vacuna contra el COVID-19
- ☐ Ya no me preocupan los efectos secundarios
- ☐ Ya no prefiero adquirir inmunidad tras contraer COVID-19, sin necesidad de una vacuna
- ☐ Ahora confío en el gobierno
- ☐ Ya no temo que las vacunas se están siendo desarolladas demasiado rápido
- ☐ Ya no temo que la vacuna me dará COVID-19
- ☐ Ahora pienso que las vacunas son efectivas contra el COVID-19
- ☐ Ya no temo que no podré pagar una vacuna para el COVID-19
- ☐ ________ Otra:

These page timer metrics will not be displayed to the recipient.
First Click: *0 seconds*
Last Click: *0 seconds*
Page Submit: *0 seconds*
Click Count: *0 clicks*

Post-treatment questions

¿Hasta qué punto está usted de acuerdo o en desacuerdo?

Si una vacuna contra el COVID-19 estuviera disponible, yo me vacunaría.

- Muy en desacuerdo
- En desacuerdo
- Ni de acuerdo ni en desacuerdo
- De acuerdo
- Muy de acuerdo
- No estoy seguro

These page timer metrics will not be displayed to the recipient.
First Click: *0 seconds*
Last Click: *0 seconds*
Page Submit: *0 seconds*
Click Count: *0 clicks*

Si una vacuna contra el COVID-19 estuviera disponible para usted ahora, ¿cuántos meses esperaría antes de vacunarse?

- [ ] Numero de meses:
- Nunca tomaría una vacuna

These page timer metrics will not be displayed to the recipient.
First Click: *0 seconds*
Last Click: *0 seconds*
Page Submit: *0 seconds*
Click Count: *0 clicks*

Si una vacuna contra el COVID-19 estuviera disponible para todos ahora, aproximadamente ¿qué porcentaje de personas de su municipio piensa que se vacunarían?

0	25	50	75	100

These page timer metrics will not be displayed to the recipient.
First Click: *0 seconds*
Last Click: *0 seconds*
Page Submit: *0 seconds*
Click Count: *0 clicks*

Si una vacuna contra el COVID-19 estuviera disponible para todos ahora, aproximadamente ¿qué porcentaje de personas de su municipio piensa que se vacunarían **durante los primeros dos meses de su disponibilidad**?

0	25	50	75	100

These page timer metrics will not be displayed to the recipient.
First Click: *0 seconds*
Last Click: *0 seconds*
Page Submit: *0 seconds*
Click Count: *0 clicks*

¿Qué tan probable es que motive a familiares o amigos a que se vacunen?

- Nada probable
- Poco probable
- Algo probable
- Muy probable

These page timer metrics will not be displayed to the recipient.
First Click: *0 seconds*
Last Click: *0 seconds*
Page Submit: *0 seconds*
Click Count: *0 clicks*

Para que el COVID-19 pare de propagarse, ¿qué porcentaje de personas piensa que necesitan vacunarse?

0 25 50 75 100

These page timer metrics will not be displayed to the recipient.
First Click: *0 seconds*
Last Click: *0 seconds*
Page Submit: *0 seconds*
Click Count: *0 clicks*

¿Dentro de cuántos meses cree que las cosas regresarán a la normalidad en ${e://Field/country}?

- Número de meses:
- Nunca regresarán a la normalidad

Si recibe la vacuna contra el COVID-19, ¿qué tan probable es que le diga a otros que usted se vacunó?

- Nada probable
- Poco probable
- Algo probable
- Muy probable

¿Qué tan importante es para usted vacunarse para detener la propagación del virus en su comunidad?

- Nada importante
- Poco importante
- Algo importante
- Muy importante

¿Qué tan importante es para usted vacunarse para ayudar a que todos puedan regresar a trabajar normalmente?

- Nada importante
- Poco importante
- Algo importante
- Muy importante

Conjoint experiment 1

A pesar que gente en varios países está empezando a recibir una vacuna contra el COVID-19, no habrá vacunación diponsible para todos hasta dentro de varios meses.

Además, todavía no se sabe qué vacunas estarán disponibles para la mayoría de la población.

These page timer metrics will not be displayed to the recipient.
First Click: *0 seconds*
Last Click: *0 seconds*
Page Submit: *0 seconds*
Click Count: *0 clicks*

Por tanto, nos gustaría saber qué tan probable es que decida vacunarse en base a diferentes escenarios.

Lea atentamente toda la información sobre cada escenario antes de responder a las preguntas sobre ese escenario.

These page timer metrics will not be displayed to the recipient.
First Click: *0 seconds*
Last Click: *0 seconds*
Page Submit: *0 seconds*
Click Count: *0 clicks*

*Suponga que ${e://Field/country} ha obtenido **${e://Field/vaccine_1}${e://Field/efficacy_1}***

*Esta vacuna es gratis para todos y **${e://Field/endorser_1}** recomienda que todos se vacunen lo más pronto posible.*

*La vacuna será administrada por **${e://Field/distribution_1} ${e://Field/uptake_1}***

Si esta vacuna estuviese disponible, me vacunaría.

- No
- Sí
- No sé

Si esta vacuna estuviese disponible, ¿cuántos meses esperaría para vacunarse?

- [ ] Número de meses:
- Nunca tomaría esta vacuna

Si esta vacuna estuviese disponible, ¿cuán de acuerdo está con las siguientes declaraciones?

	Muy en desacuerdo	En desacuerdo	Ni de acuerdo ni en desacuerdo	De acuerdo	Muy de acuerdo
La propagación de COVID-19 terminará rapidamente.	○	○	○	○	○
Sería muy poco probable que me dé COVID-19 si recibo esta vacuna.	○	○	○	○	○
Sería muy poco probable que sufra algún daño si recibo esta vacuna.	○	○	○	○	○
Esta campaña de vacunación del gobierno es para ayudar a los ciudadanos.	○	○	○	○	○

These page timer metrics will not be displayed to the recipient.

First Click: *0 seconds*

Last Click: *0 seconds*

Page Submit: *0 seconds*

Click Count: *0 clicks*

Conjoint experiment 2

Ahora, le mostraremos un **escenario diferente**.

*Suponga que ${e://Field/country} ha obtenido **${e://Field/vaccine_2}${e://Field/efficacy_2}***

*Esta vacuna es gratis para todos y **${e://Field/endorser_2}** recomienda que todos se vacunen lo más pronto posible.*

*La vacuna será administrada por **${e://Field/distribution_2} ${e://Field/uptake_2}***

Si esta vacuna estuviese disponible, me vacunaría.

- No
- Sí
- No sé

Si esta vacuna estuviese disponible, ¿cuántos meses esperaría para vacunarse?

- Número de meses:
- Nunca tomaría esta vacuna

Si esta vacuna estuviese disponible, ¿cuán de acuerdo está con las siguientes declaraciones?

	Muy en desacuerdo	En desacuerdo	Ni de acuerdo ni en desacuerdo	De acuerdo	Muy de acuerdo
» La propagación de COVID-19 terminará rapidamente.	○	○	○	○	○
» Sería muy poco probable que me dé COVID-19 si recibo esta vacuna.	○	○	○	○	○
» Sería muy poco probable que sufra algún daño si recibo esta vacuna.	○	○	○	○	○
» Esta campaña de vacunación del gobierno es para ayudar a los ciudadanos.	○	○	○	○	○

These page timer metrics will not be displayed to the recipient.
First Click: *0 seconds*
Last Click: *0 seconds*
Page Submit: *0 seconds*
Click Count: *0 clicks*

Conjoint experiment 3

Ahora, le mostraremos un **escenario diferente**.

*Suponga que ${e://Field/country} ha obtenido **${e://Field/vaccine_3}${e://Field/efficacy_3}***

*Esta vacuna es gratis para todos y **${e://Field/endorser_3}** recomienda que todos se vacunen lo más pronto posible.*

*La vacuna será administrada por **${e://Field/distribution_3} ${e://Field/uptake_3}***

Si esta vacuna estuviese disponible, me vacunaría.

- No
- Sí
- No sé

Si esta vacuna estuviese disponible, ¿cuántos meses esperaría para vacunarse?

- [ ] Número de meses:
- Nunca tomaría esta vacuna

Si esta vacuna estuviese disponible, ¿cuán de acuerdo está con las siguientes declaraciones?

	Muy en desacuerdo	En desacuerdo	Ni de acuerdo ni en desacuerdo	De acuerdo	Muy de acuerdo
» La propagación de COVID-19 terminará rapidamente.	○	○	○	○	○
» Sería muy poco probable que me dé COVID-19 si recibo esta vacuna.	○	○	○	○	○
» Sería muy poco probable que sufra algún daño si recibo esta vacuna.	○	○	○	○	○
» Esta campaña de vacunación del gobierno es para ayudar a los ciudadanos.	○	○	○	○	○

These page timer metrics will not be displayed to the recipient.

First Click: *0 seconds*

Last Click: *0 seconds*

Page Submit: *0 seconds*

Click Count: *0 clicks*

Conjoint experiment 4

Ahora, le mostraremos un **escenario diferente**.

*Suponga que ${e://Field/country} ha obtenido **${e://Field/vaccine_4}${e://Field/efficacy_4}***

*Esta vacuna es gratis para todos y **${e://Field/endorser_4}** recomienda que todos se vacunen lo más pronto posible.*

*La vacuna será administrada por **${e://Field/distribution_4} ${e://Field/uptake_4}***

Si esta vacuna estuviese disponible, me vacunaría.

- No
- Sí
- No sé

Si esta vacuna estuviese disponible, ¿cuántos meses esperaría para vacunarse?

- ________ Número de meses:
- Nunca tomaría esta vacuna

Si esta vacuna estuviese disponible, ¿cuán de acuerdo está con las siguientes declaraciones?

	Muy en desacuerdo	En desacuerdo	Ni de acuerdo ni en desacuerdo	De acuerdo	Muy de acuerdo
» La propagación de COVID-19 terminará rapidamente.	○	○	○	○	○
» Sería muy poco probable que me dé COVID-19 si recibo esta vacuna.	○	○	○	○	○
» Sería muy poco probable que sufra algún daño si recibo esta vacuna.	○	○	○	○	○
» Esta campaña de vacunación del gobierno es para ayudar a los ciudadanos.	○	○	○	○	○

These page timer metrics will not be displayed to the recipient.
First Click: *0 seconds*
Last Click: *0 seconds*
Page Submit: *0 seconds*
Click Count: *0 clicks*

Conjoint experiment 5

Ahora, le mostraremos un **escenario diferente**. Este es el último escenario.

*Suponga que ${e://Field/country} ha obtenido **${e://Field/vaccine_5}${e://Field/efficacy_5}***

*Esta vacuna es gratis para todos y **${e://Field/endorser_5}** recomienda que todos se vacunen lo más pronto posible.*

*La vacuna será administrada por **${e://Field/distribution_5} ${e://Field/uptake_5}***

Si esta vacuna estuviese disponible, me vacunaría.

- No
- Sí
- No sé

Si esta vacuna estuviese disponible, ¿cuántos meses esperaría para vacunarse?

- [ ] Número de meses:
- Nunca tomaría esta vacuna

Si esta vacuna estuviese disponible, ¿cuán de acuerdo está con las siguientes declaraciones?

	Muy en desacuerdo	En desacuerdo	Ni de acuerdo ni en desacuerdo	De acuerdo	Muy de acuerdo
» La propagación de COVID-19 terminará rapidamente.	○	○	○	○	○
» Sería muy poco probable que me dé COVID-19 si recibo esta vacuna.	○	○	○	○	○
» Sería muy poco probable que sufra algún daño si recibo esta vacuna.	○	○	○	○	○
» Esta campaña de vacunación del gobierno es para ayudar a los ciudadanos.	○	○	○	○	○

These page timer metrics will not be displayed to the recipient.

First Click: *0 seconds*

Last Click: *0 seconds*

Page Submit: *0 seconds*

Click Count: *0 clicks*

Open-ended question and nationality

Pensando en los diferentes escenarios que usted ha visto, ¿qué factores lo harían más dispuesto a tomar una vacuna sobre otra? Por favor, sea breve en su respuesta.

____

¿Cuál es su nacionalidad?

Behavioral question

¿Quisiera recibir un link de la Organización Panamericana de la Salud con más información sobre las vacunas del COVID-19?

Si usted selecciona sí, lo verá en la siguiente pantalla.

- No
- Sí

These page timer metrics will not be displayed to the recipient.
First Click: *0 seconds*
Last Click: *0 seconds*
Page Submit: *0 seconds*
Click Count: *0 clicks*

Este es el enlace: haga click aquí.

Este enlace abrirá en una nueva pestaña; por favor recuerde completar la encuesta.

These page timer metrics will not be displayed to the recipient.
First Click: *0 seconds*
Last Click: *0 seconds*
Page Submit: *0 seconds*
Click Count: *0 clicks*

Powered by Qualtrics
